# Neoadjuvant nivolumab or nivolumab plus LAG-3 inhibitor relatlimab in resectable esophageal/gastroesophageal junction cancer: a phase Ib trial and ctDNA analyses

**DOI:** 10.1038/s41591-024-02877-z

**Published:** 2024-03-19

**Authors:** Ronan J. Kelly, Blair V. Landon, Ali H. Zaidi, Dipika Singh, Jenna V. Canzoniero, Archana Balan, Russell K. Hales, K. Ranh Voong, Richard J. Battafarano, Blair A. Jobe, Stephen C. Yang, Stephen Broderick, Jinny Ha, Kristen A. Marrone, Gavin Pereira, Nisha Rao, Aryan Borole, Katerina Karaindrou, Zineb Belcaid, James R. White, Suqi Ke, Ali I. Amjad, Benny Weksler, Eun Ji Shin, Elizabeth Thompson, Kellie N. Smith, Drew M. Pardoll, Chen Hu, Josephine L. Feliciano, Valsamo Anagnostou, Vincent K. Lam

**Affiliations:** 1 The Charles A. Sammons Cancer Center, Baylor University Medical Center, Dallas, TX USA; 2 The Sidney Kimmel Comprehensive Cancer Center, Johns Hopkins University School of Medicine, Baltimore, MD USA; 3 Allegheny Health Network Cancer Institute, Allegheny Health Network, Pittsburgh, PA USA; 4 The Bloomberg-Kimmel Institute of Cancer Immunotherapy, Johns Hopkins University School of Medicine, Baltimore, MD USA; 5 Department of Radiation Oncology, Johns Hopkins University School of Medicine, Baltimore, MD USA; 6 Department of Surgery, Johns Hopkins University School of Medicine, Baltimore, MD USA; 7 Department of Biostatistics, Bloomberg School of Public Health, Johns Hopkins University, Baltimore, MD USA; 8 Department of Gastroenterology & Hepatology, Johns Hopkins University School of Medicine, Baltimore, MD USA; 9 Department of Pathology, Johns Hopkins University School of Medicine, Baltimore, MD USA; 10 Lung Cancer Precision Medicine Center of Excellence, Johns Hopkins University School of Medicine, Baltimore, MD USA

**Keywords:** Phase II trials, Translational research, Cancer genomics

## Abstract

Gastroesophageal cancer dynamics and drivers of clinical responses with immune checkpoint inhibitors (ICI) remain poorly understood. Potential synergistic activity of dual programmed cell death protein 1 (PD-1) and lymphocyte-activation gene 3 (LAG-3) inhibition may help improve immunotherapy responses for these tumors. We report a phase Ib trial that evaluated neoadjuvant nivolumab (Arm A, *n* = 16) or nivolumab–relatlimab (Arm B, *n* = 16) in combination with chemoradiotherapy in 32 patients with resectable stage II/stage III gastroesophageal cancer together with an in-depth evaluation of pathological, molecular and functional immune responses. Primary endpoint was safety; the secondary endpoint was feasibility; exploratory endpoints included pathological complete (pCR) and major pathological response (MPR), recurrence-free survival (RFS) and overall survival (OS). The study met its primary safety endpoint in Arm A, although Arm B required modification to mitigate toxicity. pCR and MPR rates were 40% and 53.5% for Arm A and 21.4% and 57.1% for Arm B. Most common adverse events were fatigue, nausea, thrombocytopenia and dermatitis. Overall, 2-year RFS and OS rates were 72.5% and 82.6%, respectively. Higher baseline programmed cell death ligand 1 (PD-L1) and LAG-3 expression were associated with deeper pathological responses. Exploratory analyses of circulating tumor DNA (ctDNA) showed that patients with undetectable ctDNA post-ICI induction, preoperatively and postoperatively had a significantly longer RFS and OS; ctDNA clearance was reflective of neoantigen-specific T cell responses. Our findings provide insights into the safety profile of combined PD-1 and LAG-3 blockade in gastroesophageal cancer and highlight the potential of ctDNA analysis to dynamically assess systemic tumor burden during neoadjuvant ICI that may open a therapeutic window for future intervention. ClinicalTrials.gov registration: NCT03044613.

## Abstract (web summary)

In a phase Ib trial, neoadjuvant nivolumab or nivolumab/relatlimab prior to chemoradiotherapy were well tolerated and liquid biopsy analyses show that undetectable ctDNA was associated with longer survival.

## Main

Over the past few decades, there have been limited therapeutic advances in the clinical management of resectable gastroesophageal cancers, with the majority of patients experiencing disease progression and death within 5 years from diagnosis^1^. Recently, the phase III CheckMate 577 study resulted in Food and Drug Administration (FDA) approval of adjuvant nivolumab in patients with completely resected esophageal/gastroesophageal junction (E/GEJ) cancer with residual pathologic disease after neoadjuvant chemoradiotherapy (CRT)^2^, which represents a paradigm shift in the management of operable stage II/stage III disease. Early stage gastroesophageal tumors may express high levels of programmed cell death ligand 1 (PD-L1), indicating the presence of an adaptive immune resistance mechanism that may be reversed by anti-PD-1 antibodies^3^. Preclinical and human studies have indicated that neoadjuvant CRT may have a PD-L1 priming effect in operable E/GEJ cancer^4,5^, supporting the rational combination of CRT with immune checkpoint inhibitors (ICI). Lymphocyte-activation gene 3 (LAG-3) is a co-inhibitory receptor that is highly expressed in gastroesophageal cancers; therefore, combined anti-LAG-3 and anti-PD-1 therapy has the potential to modulate immune checkpoint pathways, re-invigorate exhausted T cells and thus enhance antitumor immune responses^6,7^. Limited data exist for the use of neoadjuvant PD-1 pathway inhibitors in combination with CRT in operable E/GEJ cancer with conflicting results to date^8–10^.

While the integration of immunotherapy in the therapeutic paradigm of gastroesophageal cancer is of paramount importance, the broad efficacy of immunotherapy in this disease remains elusive^11^. There has been an expanding application of neoadjuvant immunotherapy for operable cancer based on the notion and evidence that immunotherapy capitalizes on the primary tumor as a source of tumor antigens that could ‘prime’ tumor-specific T cells to seek out micrometastatic disease that ultimately drives recurrence after curative-intent surgery^12,13^. To this end, assessments of systemic tumor burden by analyses of circulating tumor DNA (ctDNA) may accurately and rapidly determine tumor regression with neoadjuvant ICI^14,15^ in conjunction with pathologic and functional T cell responses^12^. Preoperative ctDNA assessments may identify individuals more likely to attain pathologic complete responses with neoadjuvant ICI and postoperative ctDNA detection may identify patients with minimal residual disease (MRD) and an increased risk of disease relapse that may benefit from sequential therapy^16^. Nevertheless, the predictive versus prognostic role of ctDNA remains unclear, with no studies to date in patients with gastroesophageal cancer treated in the neoadjuvant immuno-CRT setting. Here we present safety, feasibility and efficacy, alongside pathological response, circulating tumor burden contraction and systemic neoantigen-specific T cell responses during neoadjuvant nivolumab or nivolumab plus relatlimab combined with CRT in patients with operable stage II/stage III E/GEJ cancer (ClinicalTrials.gov registration: NCT03044613).

## Results

### Study design and endpoints

**Fig. 1:**
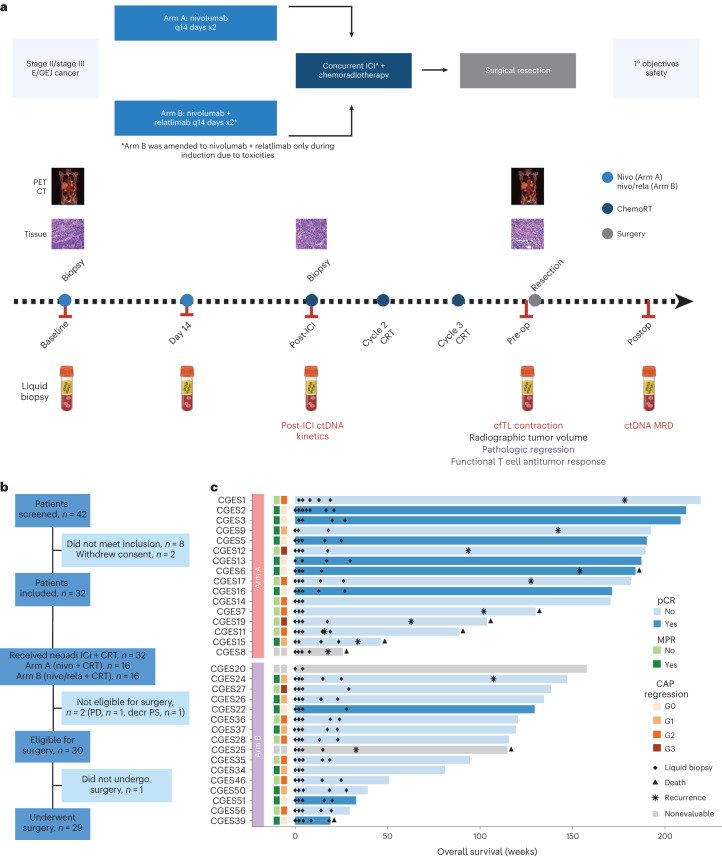
Clinical trial schema, CONSORT flow diagram and patient characteristics. **a**, Patients with resectable clinical stage II/stage III distal E/GEJ adenocarcinoma or SCC were consecutively enrolled in the following two treatment cohorts: nivolumab every 2 weeks for two induction cycles then three additional doses given concurrently with chemoradiation (Arm A) or nivolumab and relatlimab every 2 weeks according to the same schedule (Arm B). Patients were enrolled in Arm B after safety and feasibility objectives were met in Arm A. The primary endpoint of the trial was safety; the secondary endpoint was feasibility; exploratory endpoints included OS, RFS, MPR and pCR rates and biomarker analyses. Baseline CT and PET/CT scans were obtained before the first dose of neoadjuvant treatment, and PET/CT was obtained after completion of neoadjuvant treatment (presurgery). Tumor samples were collected at baseline, after two cycles of induction immunotherapy, and at the time of surgery. Serial blood samples were collected at baseline, start of cycle 2, start of cycle 3, before surgery and within 3–12 weeks after surgery. **b**, CONSORT flow diagram depicting patient disposition as follows: of the 42 patients screened, 8 did not meet inclusion criteria and 2 withdrew consent. The remaining 32 patients were enrolled in the study; 2 patients were not eligible for surgery (1 patient because of disease progression—PD—and 1 patient because of declining performance status related to CRT). Of the 30 patients eligible for surgery, 1 patient elected not to undergo surgery and the remaining 29 patients underwent Ivor Lewis esophagectomy. **c**, Swimmer’s plot depicting pCR, MPR, CAP tumor regression, recurrence, death and OS, together with blood collection for liquid biopsy analyses for each patient. Patients are grouped by trial arm and ordered by OS within each arm. The bar color indicates pCR. CONSORT, Consolidated Standards of Reporting Trials; ICI, immune checkpoint inhibitors; neoadj, neoadjuvant; PD, progressive disease; cfTL, cell-free tumor load.

Patients aged 18 years and older, with clinical stage II/stage III distal E/GEJ adenocarcinoma or squamous cell carcinoma (SCC) were eligible to enroll in the study. All patients had to have surgically resectable disease and be a candidate for standard of care CRT followed by surgery. The following two treatment cohorts were consecutively enrolled: (1) nivolumab every 2 weeks for two induction cycles then three additional doses given concurrently with CRT (Arm A) or (2) nivolumab and relatlimab every 2 weeks according to the same schedule (Arm B; Fig. 1a,b). The primary endpoint of the trial was safety; the secondary endpoint was feasibility; exploratory endpoints included overall survival (OS), recurrence-free survival (RFS), major pathological response (MPR) and pathological complete response (pCR) rates. Exploratory analyses of serial ctDNA and assessment of neoantigen-specific T cells in peripheral blood together with gene expression analyses were also performed on serial biospecimens in each arm (Fig. 1a).

### Patient characteristics and treatment

From August 2017 to July 2021, 42 patients were screened and 32 patients were enrolled (Fig. 1b). Their clinical and pathological characteristics are summarized in Table 1. Patients predominantly had adenocarcinoma histology (87.5%), primary esophageal tumors (81.3%) and nodal involvement (75.0%). Sixteen patients received nivolumab as induction for two cycles then in combination with CRT for a total of five doses (Arm A). In Arm B, the first 9 of 16 patients received nivolumab and relatlimab following the same schedule as in Arm A, while the remaining 7 patients only received nivolumab and relatlimab as induction due to a protocol amendment for toxicity (Supplementary Table 1). Overall, 28 patients completed the full course of neoadjuvant therapy and four patients (one patient in Arm A and three patients in Arm B) required ICI discontinuation due to immunotherapy-related adverse events (irAEs; Supplementary Table 2). Nivolumab and relatlimab combined with CRT demonstrated unacceptable toxicity, requiring a protocol amendment per predefined early stopping rules (Supplementary Table 1). Six of the first nine (66%) patients treated with dual ICI plus CRT developed grade 3 or higher irAEs including pericarditis (2 of 9, 22%) and adrenal insufficiency (2 of 9, 22%; Supplementary Table 2). The nivolumab/relatlimab arm was thus amended to include two cycles of nivolumab and relatlimab only as induction before chemoradiation and was subsequently well tolerated. All patients received both cycles of induction ICI and 92.1% of planned systemic therapy cycles were administered. After completion of neoadjuvant therapy, two patients were ineligible for surgery (due to disease progression, *n* = 1 and CRT-related decreased performance status, *n* = 1) and one patient declined surgery. Twenty-nine surgical candidates underwent Ivor Lewis esophagectomy within a median of 8 weeks from completion of CRT (range: 3.6–11.4 weeks). Eight (28%) patients received adjuvant therapy as per standard of care—three patients in Arm A received adjuvant FOLFOX (folinic acid, 5-fluouracil and oxaliplatin; median duration 12 weeks) and four patients in Arm B received adjuvant nivolumab (all ongoing at the time of data lock).

**Table 1:** Patient and tumor baseline characteristics

Characteristics	Whole cohort (*N* = 32)	Arm A (*n* = 16)	Arm B (*n* = 16)
Age—mean (s.d.)	63 (7.95)	60.25 (9.5)	65.75 (4.91)
Age—median (range)	65 (39, 73)	61 (39, 73)	66 (57, 72)
Sex—no. (%)			
Male	26 (81.25)	13 (81.25)	13 (81.25)
Female	6 (18.75)	3 (18.75)	3 (18.75)
Smoking—no. (%)			
Never	17 (53.12)	9 (56.25)	8 (50)
Former	15 (46.88)	7 (43.75)	8 (50)
Clinical stage—no. (%)			
Stage II	20 (62.5)	9 (56.25)	11 (68.75)
Stage III	12 (37.5)	7 (43.75)	5 (31.25)
Clinical N stage—no (%)			
Node positive	24 (75.0)	12 (75.0)	12 (75.0)
Anatomic location—no. (%)			
Esophagus	26 (81.25)	13 (81.25)	13 (81.25)
GEJ	6 (18.75)	3 (18.75)	3 (18.75)
Histology—no. (%)			
Adenocarcinoma	28 (87.5)	14 (87.5)	14 (87.5)
SCC	4 (12.5)	2 (12.5)	2 (12.5)
PD-L1 expression—no (%)			
CPS <1	11 (34.4)	4 (25)	7 (43.8)
CPS ≥1 < 5	5 (15.6)	2 (12.5)	3 (18.8)
CPS ≥5 < 10	4 (12.5)	2 (12.5)	2(12.5)
CPS ≥ 10	9 (28.1)	5 (31.2)	4 (25)
Nonevaluable	3 (9.4)	3 (18.8)	0 (0)

### Safety and feasibility

**Extended Data Fig. 1:**
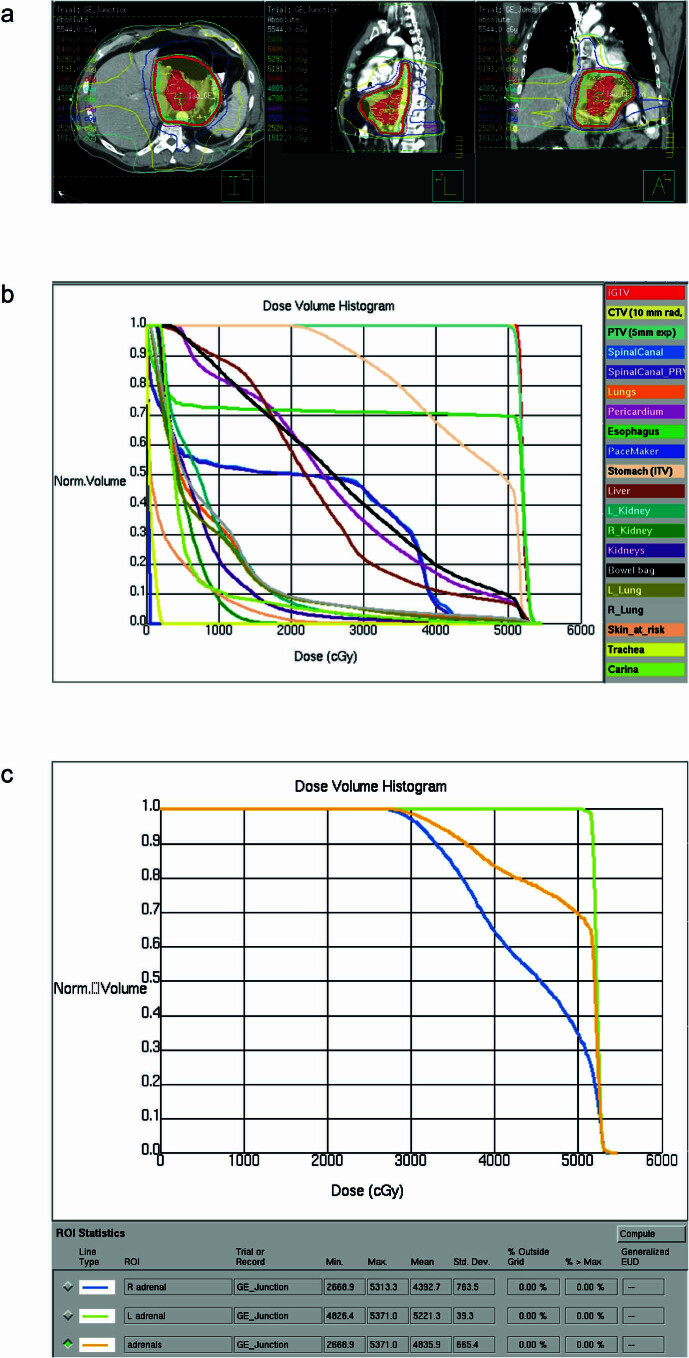
Radiotherapy plan for patient CGES22. **(a)** Representative radiotherapy plan of patient CGES22 who developed adrenal insufficiency toxicity. Radiotherapy DICOMs are shown in representative axial, sagittal and coronal slices. **(b)** Dose volume histogram (DVH) of target and normal structures; all target and normal structure dose objectives met protocol-specified markers of plan quality, which mirror national guidelines. **(c)** DVH of right, left and combined adrenal glands; listed below the DVH are dose statistics (max, mean) for adrenal glands. As there are no metrics for goal dose in adrenal glands in the literature, these are contoured and reported after therapeutic radiation administration.

**Extended Data Fig. 2:**
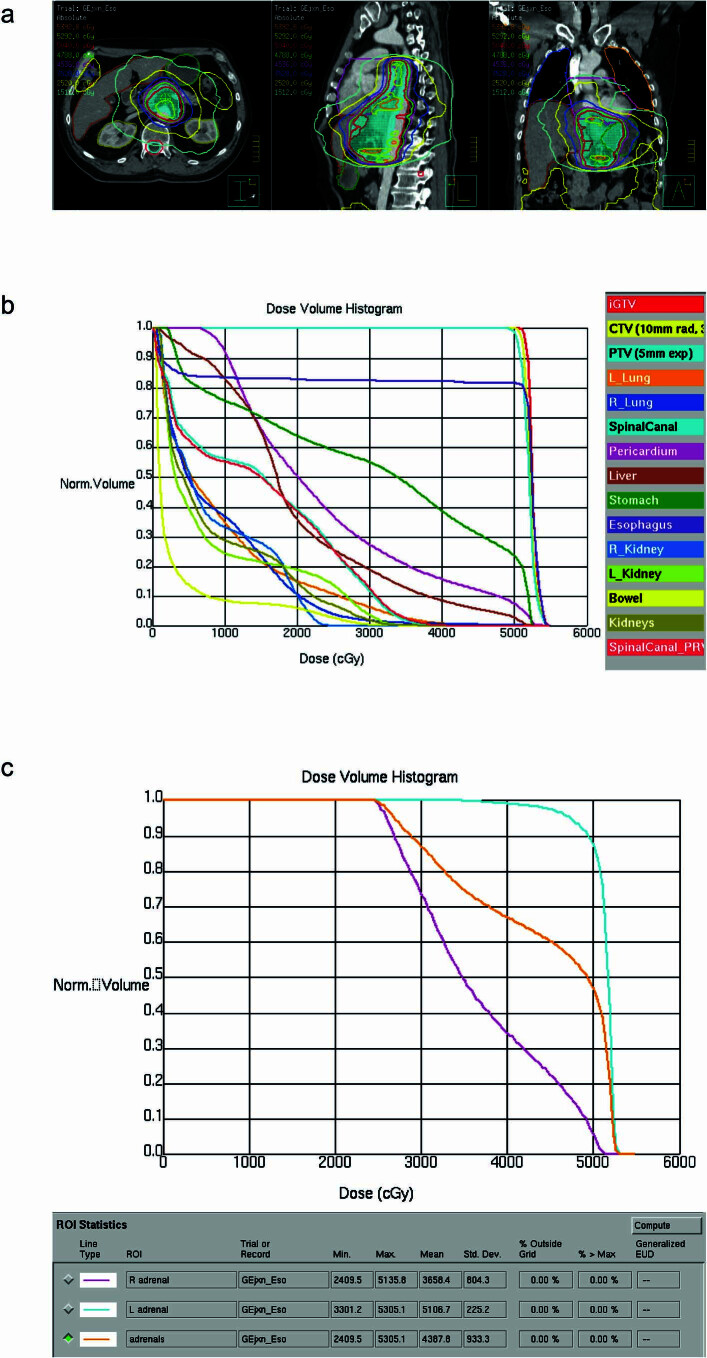
Radiotherapy plan for patient CGES26. **(a)** Representative radiotherapy plan of patient CGES26 who experienced adrenal insufficiency toxicity. Radiotherapy DICOMs are shown in representative axial, sagittal and coronal slices. **(b)** Dose volume histogram (DVH) of target and normal structures; all target and normal structure dose objectives met protocol-specified markers of plan quality, which mirror national guidelines. **(c)** DVH of right, left and combined adrenal glands; listed below the DVH are dose statistics (max, mean) for adrenal glands. As there are no metrics for goal dose in adrenal glands in the literature, these are contoured and reported after therapeutic radiation administration.

The clinical trial met its primary endpoint of safety for Arm A, which evaluated nivolumab plus chemoradiation, but required an amendment in Arm B to mitigate toxicity. Treatment-related adverse events (TRAEs) of any grade occurred in all patients, most often related to chemotherapy or radiation (Supplementary Table 2). Grade 3 or higher TRAEs occurred in 31.3% (95% confidence interval (CI): 16.1–50.0%) of patients (18.8%, 95% CI: 4.0–45.6% in Arm A and 43.8%, 95% CI: 19.8–70.1 in Arm B). Overall, 17 (53.1%, 95% CI: 34.7–70.9%) patients experienced irAEs (31.3%, 95% CI: 11.0–58.7% in Arm A and 75.0%, 95% CI: 47.6–92.7% in Arm B), although generally low grade, with dermatitis (31.3%), elevated AST (aspartate aminotransferase)/ALT (alanine transaminase; 12.5%) and hypothyroidism (12.5%) observed most frequently. There was no grade 3 or higher treatment-related pneumonitis or acute respiratory failure. One patient in Arm B experienced grade 2 pneumonitis, most consistent with a radiation-induced process that resolved with a short course of prednisone already prescribed for concomitant irAE dermatitis. Grade 3 or higher irAEs occurred in 8 (25.0%, 95% CI: 11.5–43.4%) of all treated patients, more prevalent with the dual ICI regimen (2 (12.5% (95% CI: 0.016–38.3%)) in Arm A and 6 (37.5%, 95% CI: 15.2–64.6%) in Arm B), and predominantly presenting as dermatitis (4 (12.5%, 95% CI: 3.5–29.0%)), although pericarditis (2 (6.3%)) and adrenal insufficiency (2 (6.3%)) were also notable. Both cases of pericarditis were from Arm B and required hospitalization—one patient presented after the first week of concurrent ICI and CRT with acute chest pain and EKG changes suggestive of pericarditis, which was resolved with NSAIDs (nonsteroidal anti-inflammatory drugs) and colchicine; the second patient had already completed their neoadjuvant course and presented with acute pericardial effusion in the setting of cardiogenic shock on postoperative day 11, which was resolved with pericardial window, NSAIDs and colchicine. Neoadjuvant immunotherapy was discontinued in four patients due to grade 3 irAEs—two with dermatitis, one with elevated liver enzymes and one with pericarditis. With respect to primary adrenal insufficiency, both patients received significant radiation to the adrenal glands; however, adrenal insufficiency was likely related to dual ICI rather than radiation (Extended Data Figs. 1 and 2). Overall, irAEs necessitated immunotherapy hold in 18.8% of patients (95% CI: 7.2–36.4%), 2 (12.5%, 95% CI: 1.6–38.3%) in Arm A and 4 (25.0%, 95% CI: 7.3–52.4%) in Arm B); or discontinuation in 12.5% of patients (95% CI: 3.5–29.0%), 1 (6.3%, 95% CI: 0–30.2%) in Arm A and 3 (18.8%, 95% CI: 4.0–45.6%) in Arm B, respectively. None of the seven patients in the amended Arm B that received immunotherapy only as induction experienced an irAE-related treatment hold or discontinuation. There was one death in the immediate postoperative period due to septic shock unrelated to systemic therapy.

Feasibility was assessed through the proportion of eligible patients who proceeded to surgery without substantial treatment-related delay; the latter was defined as more than 11 weeks from completion of chemoradiation. A Bayesian continuous monitoring plan was used to monitor if the proportion was evidently greater than 75% (for example, at most 25% of cases with substantial delays). Of the patients eligible for surgery after completion of neoadjuvant therapy, one patient declined surgery (Arm B) and another patient underwent resection at 11.4 weeks after chemoradiation due to travel logistics (Arm B). All other operative candidates proceeded to planned surgery without substantial delay (100% feasibility).

### Efficacy and pathological response

**Fig. 2:**
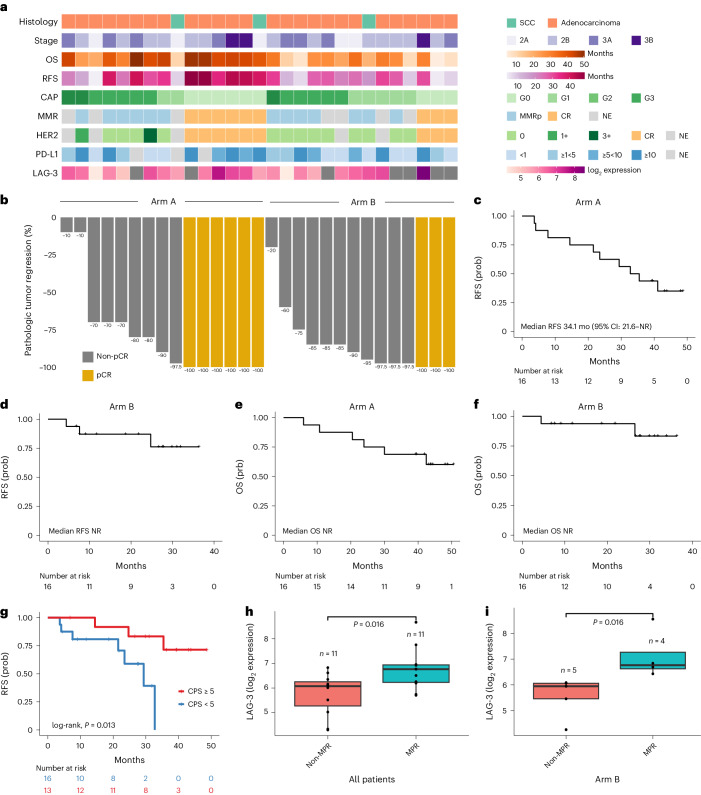
Clinical outcomes, pathological response and biomarker expression. **a**, Select clinical and pathological features for each patient that underwent resection after neoadjuvant nivolumab + CRT (Arm A; *n* = 15) and nivolumab + relatlimab + CRT (Arm B; *n* = 14). **b**, Waterfall plot of pathological tumor regression (computed as % viable tumor − 100%) for each patient. **c**, Kaplan–Meier curve of probability of RFS in all patients treated in Arm A (*n* = 16). **d**, Kaplan–Meier curve of probability of RFS in all patients treated in Arm B (*n* = 16). **e**, Kaplan–Meier curve of the probability of OS in all patients treated in Arm A (*n* = 16). **f**, Kaplan–Meier curve of the probability of OS in all patients treated in Arm B (*n* = 16). **g**, Kaplan–Meier curve of probability of RFS in patients based on baseline tumor PD-L1 CPS; patients with a CPS ≥5 at baseline (*n* = 16) had a longer RFS compared to patients with a CPS <5 (*n* = 13; median RFS not reached versus 29.34 months for CPS ≥5 and <5, respectively; log-rank, *P* = 0.013). **h**, Patients who attained an MPR (*n* = 11) had a higher LAG-3 expression at baseline compared to patients with a non-MPR (*n* = 11; median LAG-3 expression 6.68 versus 6.01, respectively, two-sided Wilcoxon rank-sum test, *P* = 0.016). **i**, These findings were driven by Arm B, as patients who attained an MPR (*n* = 4) had a higher baseline LAG-3 expression (median LAG-3 expression 6.77 versus 5.95, respectively, two-sided Wilcoxon rank-sum test, *P* = 0.016). All box plots depict the median value, with the lower and upper hinge corresponding to the first and third quartiles, respectively. The upper whisker extends from the upper hinge to at most 1.5× interquartile range and the lower whisker extends from the lower hinge to at most 1.5× interquartile range. MMRp, mismatch repair proficiency. Prob, probability.

In the cohort of 29 patients that underwent surgery, the pCR rate was 40.0% (95% CI: 16.3–67.7%) in Arm A and 21.4% (95% CI: 4.7–50.8%) in Arm B (Fig. 1c, Fig. 2a,b and Supplementary Table 3). Nine (31.03%) patients had a College of American Pathologists (CAP) tumor regression score of grade 0 (G0), seven G1 (24.14%), ten G2 (34.48%) and three G3 (10.34%), corresponding to zero, 1–10%, 11–50% and 51–100% residual viable tumor, respectively (Fig. 1c, Fig. 2a and Supplementary Table 3). MPR, defined as ≤10% residual tumor cells after neoadjuvant therapy, occurred in 53.5% of patients (95% CI: 26.6–78.7%) in Arm A and 57.1% (95% CI: 28.9–82.3%) in Arm B (Fig. 1c and Supplementary Table 3). By histology, pCR and MPR rates were 30.8% (95% CI: 14.3–51.8%) and 50.0% (95% CI: 30.0–70.0%), respectively, for adenocarcinoma and 33.33% (95% CI: 0–90.6%) and 100.0% (95% CI: 29.2–100%), respectively, for SCC. The R0 resection rate was 100%, with a median follow-up time of 36.4 months (95% CI: 29.9–43.9) in the overall cohort, 43.9 months (95% CI: 43.2–not reached (NR)) in Arm A and 27.5 months (95% CI: 18.7–33.9) in Arm B. Median RFS was 41.1 months (95% CI: 29.4–NR); 34.1 months (95% CI: 21.6–NR) in Arm A and not reached in Arm B (Fig. 2c,d). Overall 2-year RFS rate was 72.5% (95% CI: 57.8–90.1%); 62.5% (95% CI: 42.8–91.4%) in Arm A and 87.1% (95% CI: 71.8–100%) in Arm B. Overall 2-year OS rate was 82.6% (95% CI: 69.7–97.8%); 75.0% (95% CI: 56.5–99.5%) in Arm A and 93.8% (95% CI: 82.6–100%) in Arm B. Median OS was not reached in either arm (Fig. 2e,f).

### Biomarker expression

In evaluating baseline tumor specimens with adequate tissue for immunohistochemistry (Methods), PD-L1 combined positive score (CPS) was evaluable for 29 of 32 (90.6%) patients. Eleven tumors (11 of 29, 37.9%) were negative for PD-L1 (CPS <1), while 18 (18 of 29, 62.1%) were positive for PD-L1 (Fig. 2a, Table 1 and Supplementary Tables 4 and 5). There was no significant difference in baseline PD-L1 CPS between patients in Arm A and Arm B (Fisher’s exact, *P* = 0.95). In resected specimens, PD-L1 expression was evaluable for 15 of 20 (75%) patients with residual tumor. Three tumors (3 of 15, 20%) were negative for PD-L1 (CPS <1), while 12 (12 of 15, 80%) were positive for PD-L1 (Supplementary Table 4). Human epidermal growth factor receptor 2 (HER2) expression was evaluable in 15 of 20 (75%) resected specimens, with the majority being HER2 negative (*n* = 13, 86.67%; Supplementary Table 4). Thirteen (13 of 13, 100%) evaluable resected tumors were mismatch repair (MMR) proficient by immunohistochemistry (Supplementary Table 4).

**Extended Data Fig. 3:**
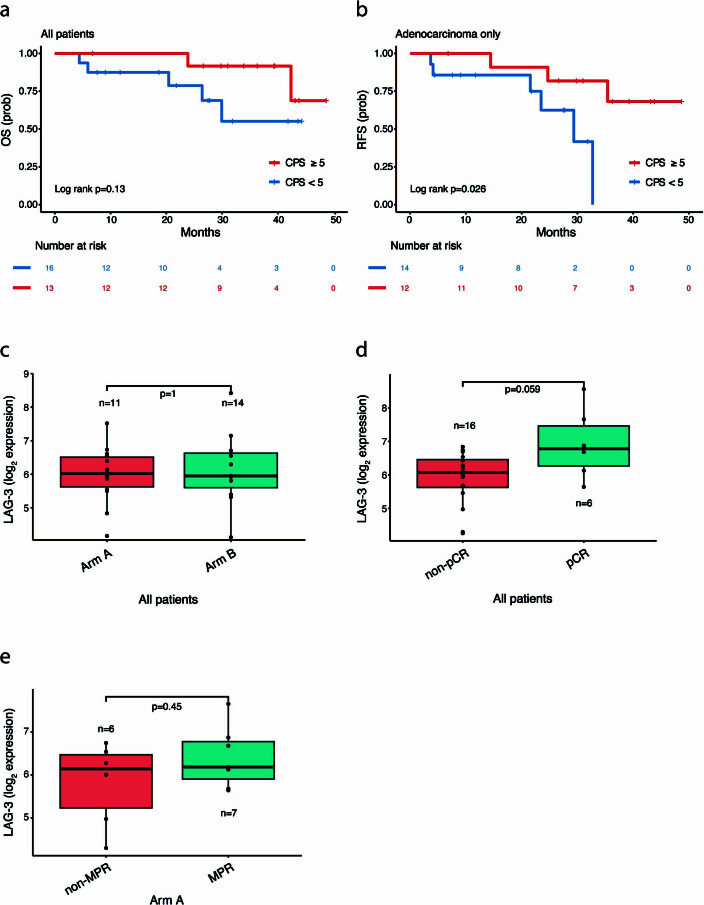
Clinical outcomes of patients with differential baseline PD-L1 and LAG-3 expression. **(a)** A trend toward a longer OS was noted for patients with a baseline PD-L1 CPS ≥5 compared to patients with a CPS <5 (log-rank p = 0.13). **(b)** When considering patients with adenocarcinoma (n = 26), patients with a baseline PD-L1 CPS ≥5 had a longer RFS compared to patients with a CPS <5 (median RFS not reached vs 29.34 months, respectively, log-rank p = 0.026). **(c)** There was no difference in normalized LAG-3 expression in baseline tumors by treatment arm (median LAG-3 normalized log_2_ expression of 6.12 vs 6.09 for arms A and B, respectively, two-sided Wilcoxon rank-sum test, p = 1). **(d)** Patients who attained a pCR showed a trend toward a higher baseline LAG-3 expression compared to patients who did not attain a pCR (median LAG-3 expression 6.78 vs 6.08, respectively, two-sided Wilcoxon rank-sum test, p = 0.059). **(e)** When considering Arm A patients (n = 13), there was no difference in baseline LAG-3 expression based on MPR status (median LAG-3 expression 6.14 vs 6.19 for non-MPR and MPR, respectively, two-sided Wilcoxon rank-sum test, p = 0.45). All box plots depict the median value, with the lower and upper hinge corresponding to the first and third quartiles, respectively. The upper whisker extends from the upper hinge to at most 1.5× the interquartile range and the lower whisker extends from the lower hinge to at most 1.5× the interquartile range.

Overall, baseline PD-L1 expression was not associated with pCR or MPR (Supplementary Table 6), with a trend toward enrichment for baseline PD-L1 CPS ≥5 in tumors with pCR (Fisher’s exact *P* = 0.089). Patients with tumors with baseline PD-L1 CPS ≥5 had a longer RFS (median RFS not reached versus 29.34 months for CPS ≥5 and <5, respectively; log-rank, *P* = 0.013; Fig. 2g), with a trend toward longer OS (median OS not reached for both CPS ≥5 and <5; log-rank, *P* = 0.13; Extended Data Fig. 3). Similarly, patients with adenocarcinomas harboring a baseline PD-L1 CPS score ≥5 had a longer RFS (median RFS not reached versus 29.34 months; log-rank, *P* = 0.026; Extended Data Fig. 3). We then evaluated changes in PD-L1 expression in resected tumors after immuno-CRT compared to baseline, but did not observe significant differences (*n* = 14; Fisher’s exact, *P* = 0.26 for all patients and *n* = 13; Fisher’s exact, *P* = 0.24 for the adenocarcinoma subset). There were no significant differences in OS or RFS for patients with PD-L1 increase after immuno-CRT (*n* = 5) compared to those without PD-L1 expression changes (*n* = 9; log-rank, *P* = 0.82 and *P* = 0.42 for OS and RFS, respectively).

As part of the study’s exploratory analyses, we evaluated the association between baseline LAG-3 expression and pathologic response (*n* = 25). There was no significant difference in baseline LAG-3 expression between Arms A and B (median LAG-3 normalized log_2_ expression of 6.12 versus 6.09, respectively; Wilcoxon rank-sum test, *P* = 1; Extended Data Fig. 3 and Supplementary Table 7). Patients who attained a pCR had a trend toward higher baseline LAG-3 expression (median LAG-3 normalized log_2_ expression 6.78 versus 6.08, respectively; Wilcoxon rank-sum test, *P* = 0.059; Extended Data Fig. 3 and Supplementary Table 7). Similarly, patients who attained an MPR had higher baseline LAG-3 expression (median LAG-3 normalized log_2_ expression 6.68 versus 6.01, respectively; Wilcoxon rank-sum test, *P* = 0.016; Fig. 2h). These findings were driven by patients in the relatlimab arm, where patients who attained an MPR had a higher baseline LAG-3 expression (median LAG-3 normalized log_2_ expression 6.77 versus 5.95, respectively; Wilcoxon rank-sum test, *P* = 0.016; Fig. 2i). In contrast, we did not find a correlation between LAG-3 expression and MPR (Wilcoxon rank-sum test, *P* = 0.45; Extended Data Fig. 3) or pCR (Wilcoxon rank-sum test, *P* = 0.22) in Arm A. Taken together, these findings suggest that LAG-3 expression may be predictive of response to combined anti-LAG-3 and anti-PD-1 therapy.

### ctDNA status correlates with tumor regression and outcomes

**Fig. 3:**
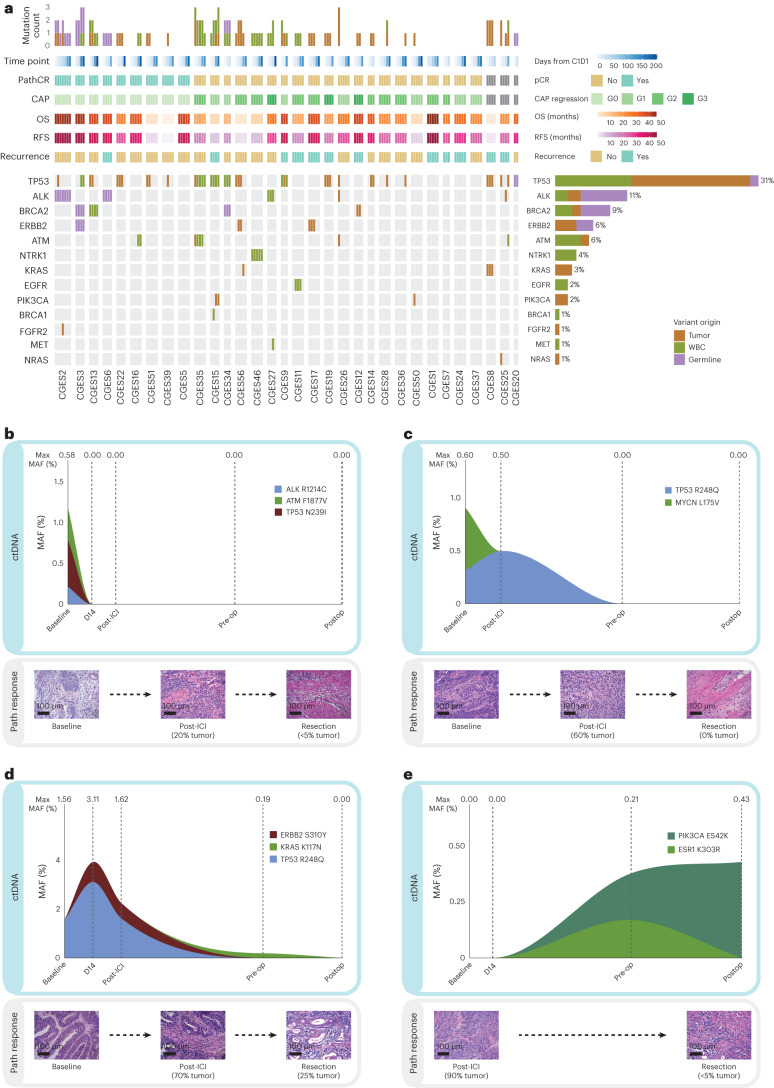
Landscape of ctDNA genomic alterations and ctDNA dynamics in patients with differential tumor regression and long-term outcomes with neoadjuvant immune checkpoint inhibition. **a**, The origin of each variant is shown along with its detection across time points. Genes displayed on the left are ones that fall within the overlapping regions of interest of the two targeted NGS gene panels used (Methods). Alteration prevalence for each gene is listed on the right. The mutation count per sample is displayed at the top followed by rows indicating sample time point, pCR, CAP regression grading, OS, RFS and recurrence. Liquid biopsy analyses revealed 74 alterations in the 141 evaluable serial plasma samples obtained from the 32 patients. The variant repertoire consisted of 12 germline-derived variants, 27 CH-derived variants and 35 tumor-derived variants. **b**, Patient CGES26, with a PD-L1 CPS of 35, cleared ctDNA on day 14 after one dose of neoadjuvant ICI. ctDNA levels remained undetectable throughout the treatment course, before surgery and postoperatively, which accurately reflected tumor regression on day 28 as well as <5% residual tumor at the time of resection; without evidence of clinical progression within 30.9 months. **c**, For patient CGES13, with a PD-L1 CPS of 5, ctDNA persistence was noted post-ICI, which was reflective of 60% residual tumor upon rebiopsy. Nevertheless, ctDNA clearance at the time of resection captured the complete tumor regression at that time point and undetectable ctDNA at the postoperative assessment was consistent with a RFS and OS of 43 months. **d**, Similarly, detectable ctDNA at the preoperative time point was reflective of residual tumor of 30% for patient CGES56, with a PD-L1 CPS of 25, who however cleared ctDNA postoperatively and this was reflected in the absence of disease recurrence. **e**, ctDNA status more accurately captured the clinical course of patient CGES15 (PD-L1 not evaluable), which showed persistence of ctDNA in the preoperative and postoperative time points despite a tumor regression of 95% at the time of resection based on pathological assessment and had disease recurrence at 7.8 months on trial. The original magnification of microscopic images is 20×; scale bar: 100 µm.

Next, we sought to evaluate the depth of tumor regression with neoadjuvant immuno-CRT at a molecular level, using ctDNA analyses, and address whether ctDNA clearance predicted RFS. As part of the study’s exploratory analyses, ultra-sensitive targeted next-generation sequencing (NGS) of 173 serial plasma and matched white blood cell (WBC) DNA samples (Supplementary Tables 8–10) allowed for the detection and longitudinal tracking of tumor-specific sequence alterations. ctDNA was assessed at baseline (before treatment initiation on trial), D14 (14 days after the start of neoadjuvant ICI), post-ICI induction (after two cycles of ICI, that is, 28 days), preoperative (before surgery) and postoperative (within 3–12 weeks after surgery). Using a tumor-agnostic, matched WBC DNA-informed deep sequencing approach coupled with a branched logic to assign variant cellular origin (Methods), we found that the plasma variant repertoire comprised 36% (27 of 74 variants) clonal hematopoiesis (CH), 16% germline (12 of 74 variants) and 47% (35 of 74 variants) tumor-derived mutations (Fig. 3a and Supplementary Table 10). At least one CH-derived variant was detected in 43.8% (14 of 32) of patients in the study cohort. Tumor-derived variants were identified in 62.5% (20 of 32) of patients, while 37.5% (12 of 32) of patients had only CH-derived or germline variants detected. Most tumor-derived variants were patient-specific; there were no recurrent mutations associated with therapeutic efficacy. Twelve patients without any tumor-derived variants detected at any time point were classified in the ctDNA undetectable group (ctDNA UD) and considered nonevaluable for subsequent circulating tumor burden assessments (Methods).

**Extended Data Fig. 4:**
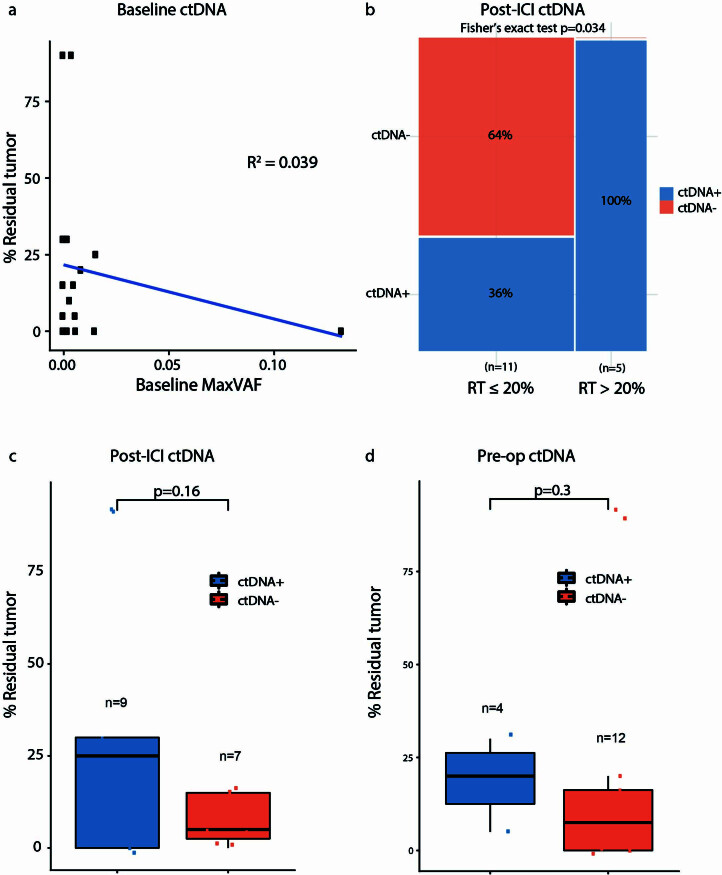
Correlation between ctDNA status at different time points and residual tumor volume at the time of surgery. **(a)** The level of ctDNA at baseline, represented by the maximal mutant allele frequency of tumor-derived variants, did not correlate with the percent of residual tumors at the time of resection (Pearson’s correlation coefficient R^2^ = 0.039). **(b)** Detectable ctDNA post-ICI induction was associated with >20% residual tumor (two-sided Fisher’s exact, p = 0.034). **(c)** Patients with undetectable ctDNA post-ICI (orange) showed a trend toward a lower residual tumor volume at the time of resection compared to patients with detectable ctDNA at that time point (blue; median residual tumor 5% vs 25%, respectively, two-sided Wilcoxon rank-sum test, p = 0.16). **(d)** Patients with undetectable ctDNA at the preop time point had a numerically lower residual tumor volume at the time of resection when compared to patients with detectable ctDNA at the same time point; however, this did not reach statistical significance (median residual tumor 7.5% vs 20%, respectively, two-sided Wilcoxon rank-sum test, p = 0.3). All box plots depict the median value, with the lower and upper hinge corresponding to the first and third quartiles, respectively. The upper whisker extends from the upper hinge to at most 1.5× the interquartile range and the lower whisker extends from the lower hinge to at most 1.5× the interquartile range.

We defined ctDNA as detected (ctDNA+) if ≥1 tumor-derived variants were detected at any mutant allele frequency (MAF), whereas ctDNA was deemed undetectable (ctDNA−) if no tumor-derived variants were detected at the specified time point assessed (Methods; Supplementary Table 11). Overall, ctDNA dynamics captured pathological tumor regression, while baseline ctDNA levels did not correlate with pathological response (Fig. 3b–e and Extended Data Fig. 4). Representative examples of ctDNA kinetics are depicted in Fig. 3b–e. Of the 20 patients with detectable ctDNA at baseline, we identified 7 (35%) patients with undetectable ctDNA at the post-ICI time point (as shown for patient CGES26 in Fig. 3b), 12 (60%) patients with undetectable ctDNA preoperatively (as shown for patient CGES13 in Fig. 3c) and 10 (50%) patients with undetectable ctDNA at the postop time point (as shown for patient CGES56 in Fig. 3d). Five patients showed sustained clearance of ctDNA at all time points, while 2 patients had ctDNA persistence throughout preoperative and postoperative sampling (as shown for patient CGES15 in Fig. 3e). There were no statistically significant differences in ctDNA status (including undetectable ctDNA) between Arms A and B of the trial; of note, a trend toward enrichment in ctDNA status post-ICI induction was observed in Arm B (Fisher’s exact, *P* = 0.065). Detectable ctDNA at the post-ICI time point correlated with residual tumor >20% at the time of resection (Fisher’s exact, *P* = 0.034; Extended Data Fig. 4), and patients with detectable ctDNA post-ICI or preoperatively had a numerically higher residual tumor content at the time of resection (Extended Data Fig. 4). ctDNA detectable status post-ICI or preoperatively was not concordant with pCR or MPR (Fisher’s exact *P* > 0.5).

**Fig. 4:**
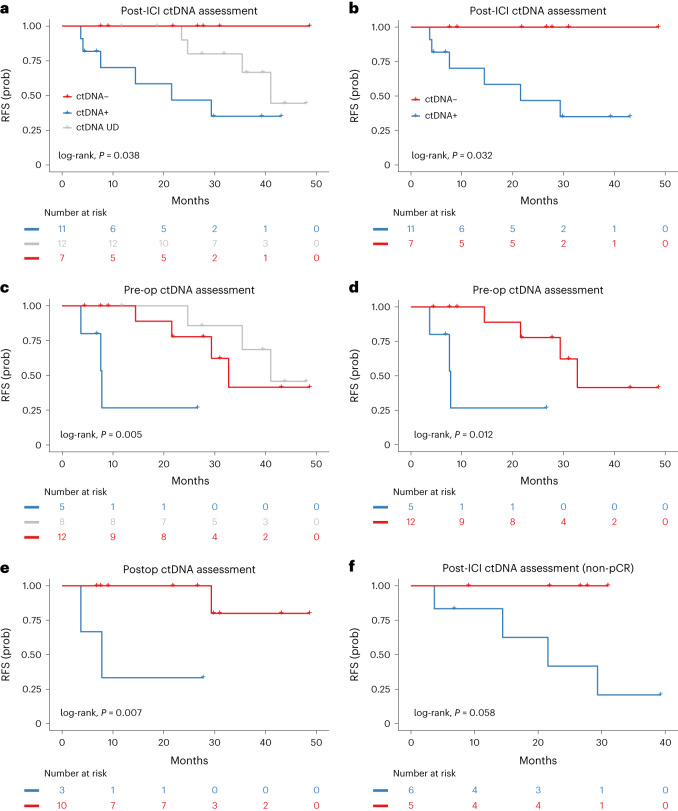
Association of ctDNA assessment and RFS. ctDNA detection was assessed at the post-ICI, preoperative and postoperative timepoints. **a**, Patients with undetectable DNA throughout the study (gray) or undetectable ctDNA post-ICI (red) had a longer RFS compared to patients with detectable ctDNA (blue) post-ICI (median RFS 41.02 months versus not reached versus 21.54 months, respectively; log-rank, *P* = 0.038). **b**, Patients with undetectable ctDNA at the post-ICI time point had a longer RFS compared to patients with detectable ctDNA post-ICI (median RFS not reached versus 21.54 months, respectively; log-rank, *P* = 0.032). **c**,**d**, Patients with undetectable ctDNA throughout the study or at the preoperative time point had a longer RFS compared to patients that had detectable ctDNA at the preoperative time point (median RFS 41.02 versus 32.72 versus 7.80 months, respectively; log-rank, *P* = 0.005 (**c**), and median RFS 32.72 versus 7.80 months, respectively; log-rank, *P* = 0.012 (**d**)). **e**, Patients with undetectable ctDNA at the postoperative time point had a longer RFS compared to patients with detectable ctDNA (median RFS not reached versus 7.80 months, respectively; log-rank, *P* = 0.007). **f**, When ctDNA was assessed among patients who did not attain a pCR, non-pCR patients with undetectable ctDNA post-ICI had a longer RFS compared to non-pCR patients with detectable ctDNA post-ICI (median RFS not reached versus 21.54, respectively; log-rank, *P* = 0.058).

**Extended Data Fig. 5:**
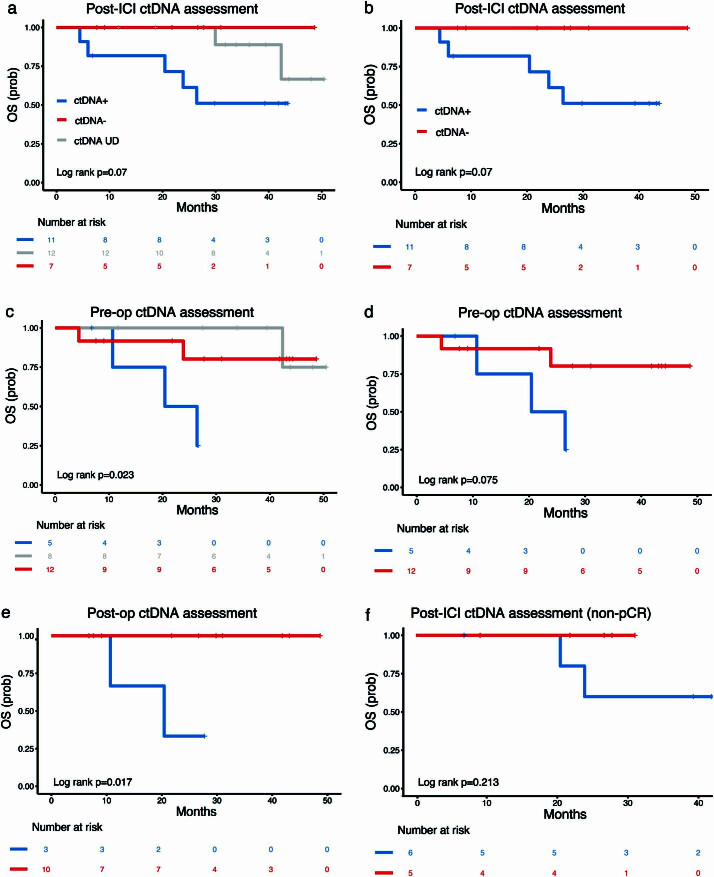
Association of ctDNA status and overall survival. **(a)** We observed a trend toward a longer overall survival for patients with undetectable ctDNA throughout the study or patients with undetectable ctDNA post-ICI compared to patients with detectable ctDNA post-ICI induction (median OS not reached for all groups, log-rank p = 0.07). **(b)** Patients with undetectable ctDNA post-ICI had a trend toward a longer OS compared to patients with detectable ctDNA at post-ICI (median OS not reached for both groups, log-rank p = 0.07). **(c)** Patients with undetectable ctDNA throughout the study or undetectable ctDNA at the preop timepoint had a longer OS compared to patients with detectable ctDNA (median OS not reached vs not reached vs 23.43 months, respectively, log-rank p = 0.023). **(d)** When patients with undetectable ctDNA throughout the study were excluded, we observed a trend toward longer OS for patients with undetectable ctDNA preop compared to patients with detectable ctDNA at that time point (median OS not reached vs 23.43 months, respectively, log-rank p = 0.075). **(e)** Patients with undetectable ctDNA postop had a longer OS compared to patients with detectable ctDNA postop (median OS not reached vs 20.43, respectively, log-rank p = 0.017). **(f)** In evaluating non-pCR patients, overall survival was not statistically significantly different with respect to post-ICI ctDNA status, likely due to the small number of cases (log-rank p = 0.213).

**Extended Data Fig. 6:**
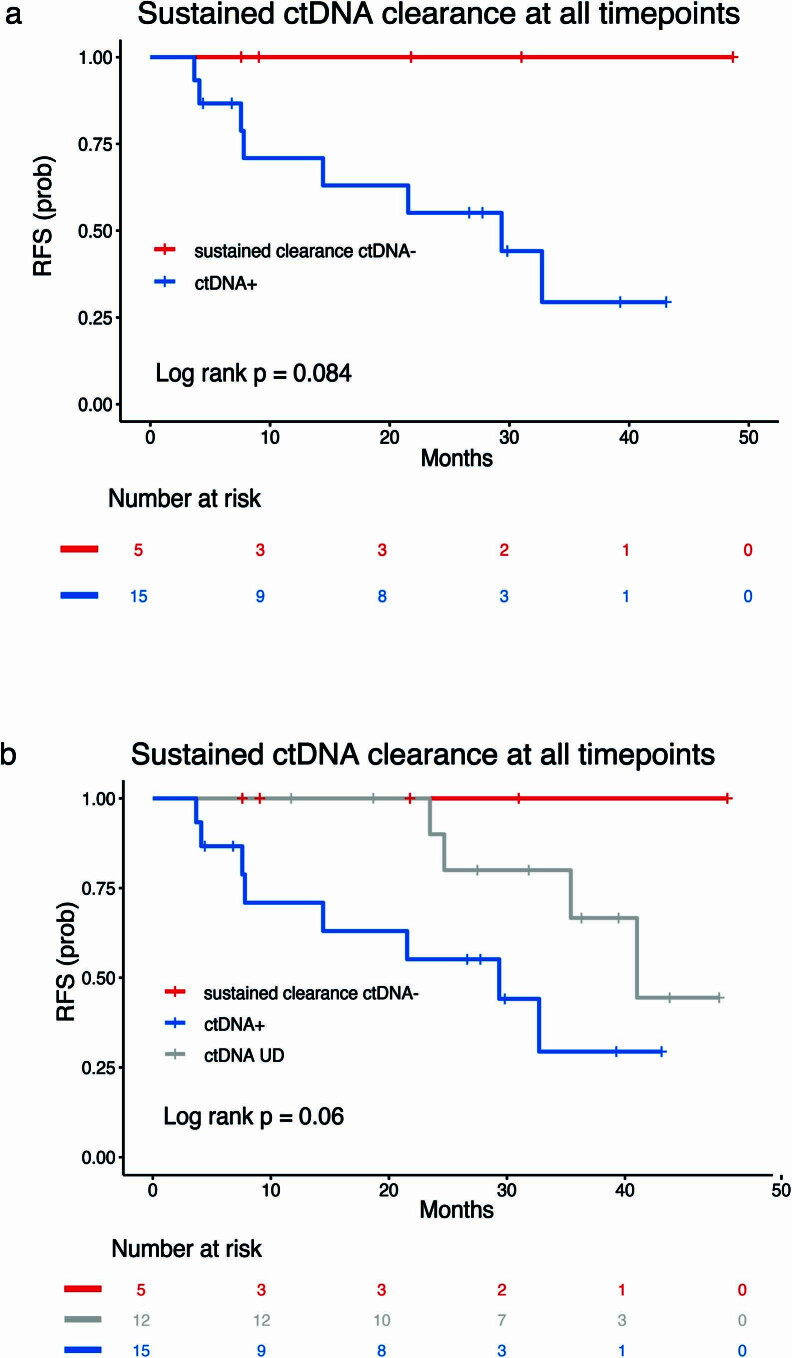
Association between clinical outcomes and sustained ctDNA clearance. ctDNA sustained clearance was defined as ctDNA presence at baseline/D14 followed by sustained clearance for the duration of the study (in all post-ICI, preop and postop time points). **(a)** Patients with sustained ctDNA clearance had a trend toward longer RFS (median RFS not reached vs 29.34 months, log-rank p = 0.084) **(b)** Patients with sustained ctDNA clearance or undetectable ctDNA throughout the study attained a numerically longer RFS; however, this finding did not reach statistical significance (median RFS 41.02 for undetectable ctDNA vs not reached for sustained ctDNA clearance vs 29.34 months for ctDNA positive, log-rank p = 0.06).

We then assessed the association of ctDNA kinetics with RFS and OS. Patients with undetectable ctDNA post-ICI had a longer RFS compared to patients with detectable ctDNA post-ICI (median RFS 41.02 months versus not reached versus 21.54 months for ctDNA UD, ctDNA− and ctDNA+, respectively; log-rank, *P* = 0.038 in Fig. 4a; log-rank, *P* = 0.032 for detectable versus undetectable post-ICI in Fig. 4b). Similarly, patients with undetectable ctDNA throughout the study or at the preoperative time point had a longer RFS compared to patients with detectable preoperative ctDNA (median RFS 41.02 versus 32.72 versus 7.80 months, respectively; log-rank, *P* = 0.005 and median RFS 32.72 versus 7.80, respectively; log-rank, *P* = 0.012 in Fig. 4c,d). In assessing ctDNA MRD, patients with undetectable ctDNA at the postoperative time point had a longer RFS compared to patients with detectable ctDNA (median RFS not reached versus 7.80, respectively; log-rank, *P* = 0.007 (Fig. 4e)). Similar trends were observed with OS (Extended Data Fig. 5). Five patients with sustained ctDNA clearance at all time points attained longer RFS (median RFS not reached versus 29.34 months; log-rank, *P* = 0.08 in Extended Data Fig. 6).

**Extended Data Fig. 7:**
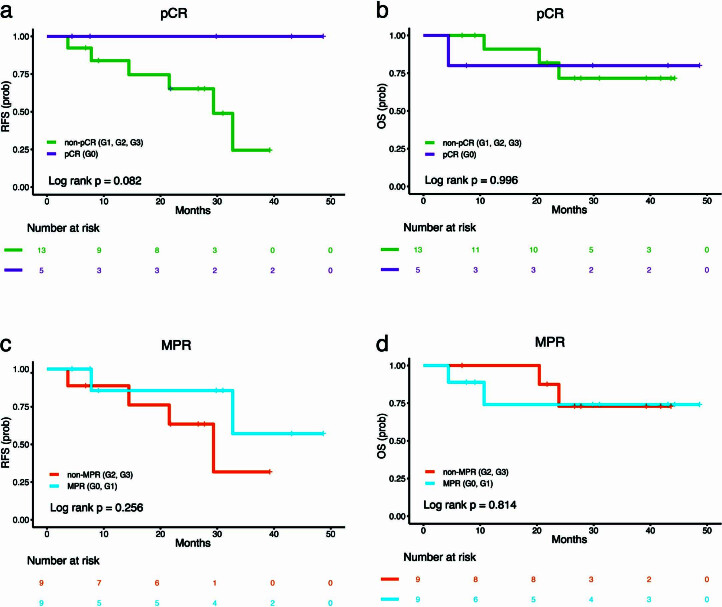
Prediction of recurrence-free and overall survival based on pathological complete or major pathological response for the subset of patients with evaluable ctDNA. **(a)** In a subset analysis that included only patients with evaluable ctDNA and pathologic responses (n = 18), we observed a trend toward a longer RFS for patients that attained a complete pathological response compared to patients that did not attain a complete pathological response (median RFS not reached vs 29.34 months, respectively, log-rank p = 0.082). **(b)** In the same subset of patients (n = 18), there were no differences noted in overall survival by pathological complete response status (median OS not reached for both groups, log-rank p = 0.996). **(c**,**d)** Similarly, for the 18 patients with evaluable ctDNA, patients with a major pathological response did not have a longer RFS or OS compared to patients that did not attain a major pathological response (median RFS not reached vs 29.34 months, respectively, log-rank p = 0.256, and median OS not reached for both groups, log-rank p = 0.814).

Notably, pCR or MPR less optimally predicted RFS and OS for the 20 patients with evaluable ctDNA (Extended Data Fig. 7). While patients with a pCR (*n* = 9) had either undetectable ctDNA throughout the study or showed ctDNA clearance, a higher heterogeneity was observed in the subset of patients with non-pCR. Among patients with non-pCR (*n* = 11), individuals with undetectable ctDNA post-ICI had a longer RFS compared to patients with detectable ctDNA (median RFS not reached versus 21.54; log-rank, *P* = 0.058 in Fig. 4f). These findings highlight the challenges with pathological response in predicting long-term clinical outcomes that may be alleviated by molecular assessments of tumor burden regression via ctDNA analyses.

**Extended Data Fig. 8:**
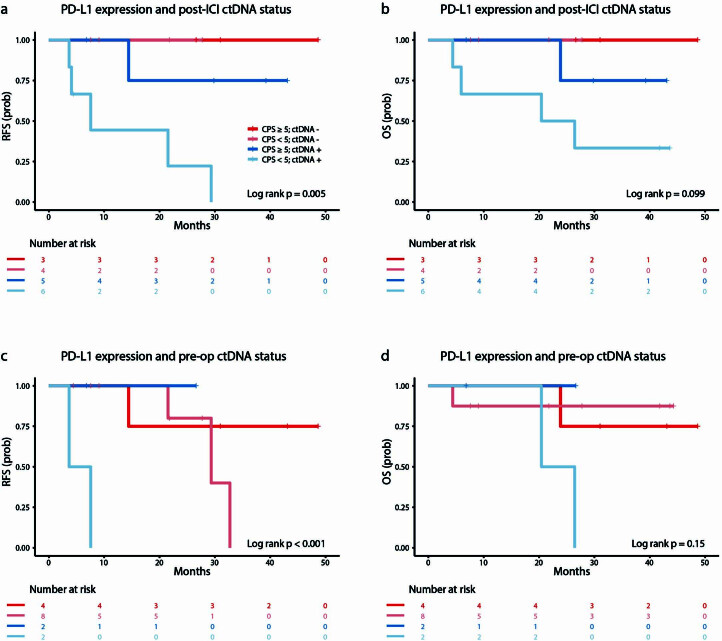
Survival analyses based on combined baseline PD-L1 expression and ctDNA status. Baseline PD-L1 CPS binarized using a CPS threshold of 5 and combined with ctDNA status post-ICI and preop. **(a**,**b)** Patients with undetectable ctDNA post-ICI had a statistically significantly longer RFS and numerically longer OS compared to patients with detectable ctDNA independent of PD-L1 CPS (log-rank p = 0.005 and p = 0.099, respectively). **(c**,**d)** Patients with detectable ctDNA preop and a CPS < 5 had a significantly shorter RFS and numerically shorter OS (log-rank p = 0.0003 and p = 0.15, respectively).

As part of the trial’s exploratory analyses, we next sought to understand the association between baseline PD-L1 expression and ctDNA kinetics by evaluating PD-L1 CPS in combination with ctDNA status. PD-L1 expression was not correlated with ctDNA status (Supplementary Table 6), suggesting that these features are largely independent. Interestingly, in evaluating ctDNA post-ICI induction together with PD-L1 expression, patients with undetectable ctDNA had a statistically significant longer RFS and numerically longer OS compared to patients with detectable ctDNA independent of PD-L1 expression (log-rank *P* = 0.0052 and long-rank *P* = 0.099; Extended Data Fig. 8); patients with positive ctDNA and PD-L1 CPS <5 had the most unfavorable outcomes. Similarly, patients with positive ctDNA preoperatively and PD-L1 CPS <5 had a significantly shorter RFS and numerically shorter OS (log-rank, *P* < 0.001 and *P* = 0.15, respectively; Extended Data Fig. 8). These findings, while limited by the small number of patients evaluated, indicate that ctDNA kinetics may more optimally capture clinical outcomes compared to PD-L1 expression.

### Neoantigen-specific T cell responses

**Fig. 5:**
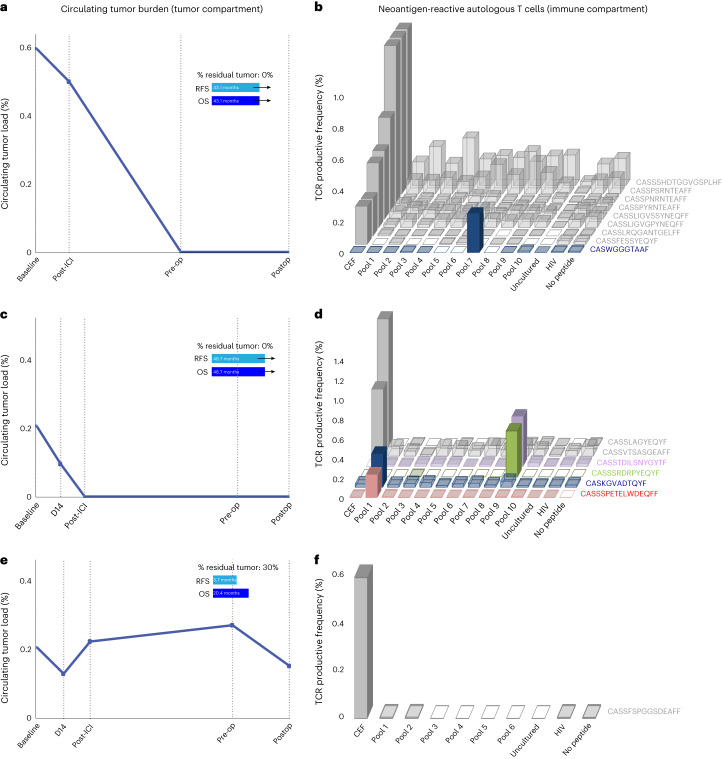
Neoantigen-specific T cell reactivity and ctDNA dynamics for patients with differential outcomes with neoadjuvant immune checkpoint inhibition. Overall, neoantigen-specific T cell responses were observed in all patients with pCR and TCR expansions mirrored systemic tumor burden regression. **a**,**b**, Patient CGES13 attained a complete pathological response, which was consistent with ctDNA clearance at the preoperative time point (**a**). In tandem, the neoantigen-specific T cell clone CASWGGGTAAF (CDR3 region) was detected expanding after pulsing with mutation-associated neoantigens contained in pool 7 (**b**). **c**,**d**, Similarly, patient CGES2 showed ctDNA clearance after two cycles of ICI (**c**), which was reflected in complete pathological response at the time of resection and neoantigen-specific clone expansions for CASSSPETELWDEQFF, CASKGVADTQYF, CASSSRDRPYEQYF and CASSTDILSNYGYTF (**d**). **e**,**f**, In contrast, patient CGES11 showed sustained ctDNA throughout the course of the study (**e**), which was reflective of a residual tumor of 30% residual tumor at the time of resection. For this patient, there were no neoantigen-specific T cell expansions noted (**f**).

**Extended Data Fig. 9:**
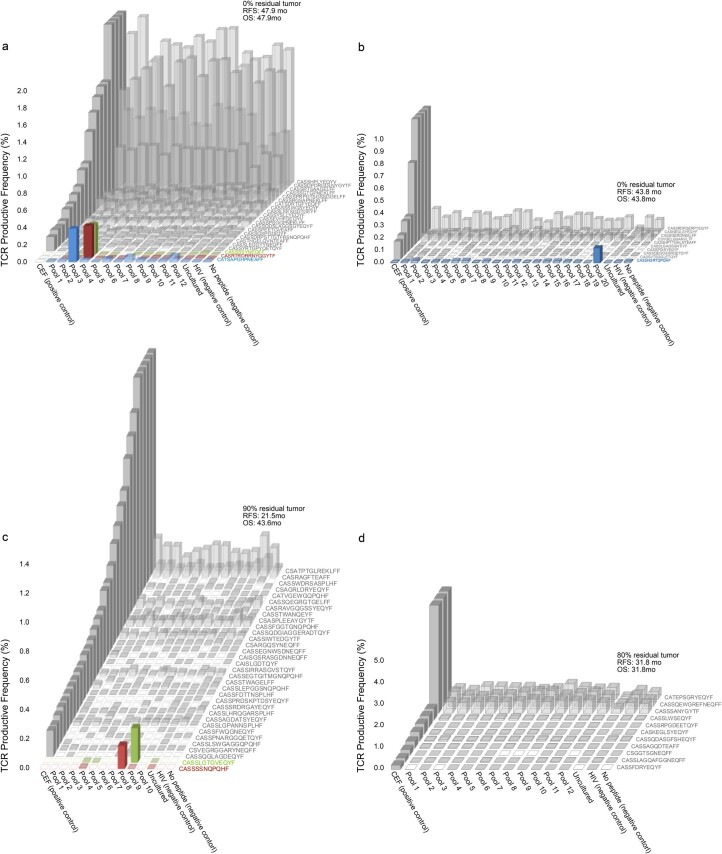
Neoantigen-specific T cell responses for patients with differential ctDNA and pathological responses. Patients CGES3 **(a)** and CGES5 **(b)** both had undetectable ctDNA throughout the study, attained a complete pathological response and did not have disease recurrence within the study interval. For patient CGES3 **(a)**, 3 neoantigen-specific TCR clone expansions were noted (CATSAPGHPNEAFF, CASRTRDRRNYGGYTF and CASSSSYNEQFF); similarly, for patient CGES5 **(b)**, one neoantigen-specific TCR clone expansion (CASSHGRTQPQHF) was noted. **(c)** Two neoantigen-specific TCR clone expansions (CASSSSNQPQHF and CASSLGTGVEQYF) were noted for patient CGES12, who had detectable ctDNA until the time of surgery reflective of 90% residual tumor at the time of resection, and later experienced disease recurrence. **(d)** No neoantigen-specific TCR clonal expansions were noted for patient CGES27, with undetectable ctDNA throughout the study and 80% residual tumor at the time of resection.

An important premise of neoadjuvant ICI is the potential to use the primary tumor as a source of tumor-specific neoantigens, thus enhancing the priming of neoantigen-specific T cells that in turn can drive systemic antitumor immune responses. To this end, following the determination of tumor regression at a pathologic level and by ctDNA clearance, we tested peripheral blood T cells for neoantigen recognition in selected patients as part of the trial’s exploratory analyses (Methods)^17,18^. Among tested patients that attained a pCR (*n* = 4), circulating neoantigen-specific T cells were detected in all cases, while among tested patients that did not attain a pCR (*n* = 3), only one patient had evidence of circulating neoantigen-specific T cells (Fig. 5, Extended Data Fig. 9 and Supplementary Tables 12–18). Notably, neoantigen-specific T cell responses were a mirror image of ctDNA kinetics, such that systemic tumor burden contraction was associated with the detection of neoantigen-specific T cells in two patients with long RFS and OS (Fig. 5a–d). Conversely, no neoantigen-specific T cell responses were detected in a patient with an RFS of 3.7 months, which was supported by ctDNA persistence throughout the treatment course (Fig. 5e,f).

## Discussion

While the therapeutic and immunologic effects of neoadjuvant ICI have been demonstrated in patients with melanoma^19^ and nonsmall cell lung cancer^12,14^, the safety, feasibility and efficacy of neoadjuvant ICI—especially in combination with CRT—together with biomarkers of clinical response have not been consistently demonstrated in patients with E/GEJ cancer^8–10^. Here we report the first clinical trial incorporating PD-1 and LAG-3 inhibition plus CRT in the neoadjuvant setting for patients with resectable E/GEJ cancers. Our findings demonstrate the safety and feasibility of this approach and suggest that ctDNA clearance is associated with systemic expansion of neoantigen-specific T cells, thus capturing systemic tumor burden and outcome-linked residual disease after neoadjuvant immunotherapy.

Safety and feasibility endpoints of the study were met, although the nivolumab and relatlimab combination in Arm B required modification to mitigate serious immune-related adverse events, in particular, pericarditis and adrenal insufficiency. The cardiac irAEs observed in Arm B may be related to the use of LAG-3 ICI with concurrent thoracic radiation and require evaluation in future studies. Two patients on the relatlimab arm developed grade 3 primary adrenal insufficiency, consistent with our understanding that immune-mediated adverse events occur more frequently with dual ICI compared to PD-1 inhibition alone. In the phase II/III RELATIVITY-047 trial, which evaluated relatlimab and nivolumab versus nivolumab alone in untreated advanced melanoma, the incidence of any grade irAE—including adrenal insufficiency—was higher in the dual ICI group^6^. It is unlikely that the adrenal insufficiency toxicities we observed in our study were related to radiation dosing, as both of the patients with adrenal insufficiency had radiation plans that met study protocol and national guideline-adherent targets and normal dose objectives.

Arm A, which evaluated nivolumab plus chemoradiation, produced higher MPR, pCR and 2-year OS rates compared to historical chemoradiation controls with 53%, 40% and 75%, respectively, compared to 32%, 29% and 67%^20^. Notably, higher pathological response rates were observed in Arm A despite having a lower proportion of patients with SCC (12.5% versus 23% in the CROSS trial), a histologic subtype shown to be most sensitive to chemoradiation in the CROSS trial^20^. The addition of relatlimab to nivolumab and chemoradiation was not associated with a higher pCR rate compared to nivolumab plus chemoradiation but appeared to numerically improve 2-year RFS and OS rates. The improved survival in the nivolumab/relatlimab arm despite increased toxicity and worse pCR rate may be consistent with the increasing body of data across multiple tumor types showing an association between irAEs and improved ICI efficacy^21^. Furthermore, pCR may not be a reliable surrogate endpoint for survival in operable gastroesophageal cancer, as multiple prospective trials have not shown improved survival despite achieving higher pCR rates^22–24^, suggesting the importance of micrometastatic disease control. Our potential signal of improved efficacy in Arm B should be interpreted with caution, especially given the higher toxicity of combined anti-PD-1/anti-LAG-3/CRT, and illustrates the need for longer-term follow-up data and larger studies to evaluate the optimal sequencing of these therapies.

High PD-L1 expression using a PD-L1 expression cutoff of CPS ≥5 has been shown to identify an enriched population of patients with gastroesophageal cancer who have favorable outcomes with ICI^25^. In line with this notion, in our study, baseline PD-L1 CPS ≥5 resulted in improved RFS and a trend toward improved OS compared with tumors with PD-L1 CPS <5. Chemoradiation has been shown to induce PD-L1 upregulation^4^, and this phenomenon may result in favorable clinical outcomes, as seen in the CheckMate 577 study, where patients with an increase of PD-L1 expression after neoadjuvant CRT who subsequently received adjuvant anti-PD-1 therapy attained a greater disease-free survival^5^. Our limited analysis of PD-L1 dynamics did not reveal any association between PD-L1 induction and outcomes; however, the small number of matched baseline and resected tumors precludes firm conclusions. LAG-3 represents a distinct immune checkpoint that mediates T cell exhaustion and as such has emerged as an immunotherapeutic target^26^. A higher LAG-3 expression has been associated with longer progression-free survival in patients with melanoma in the RELATIVITY-047 trial independent of the treatment arm (anti-PD-1 or combination anti-PD-1 and anti-LAG-3)^6^. In line with these findings, we found a higher baseline expression of LAG-3 in patients attaining a pathological response; these findings were pronounced in the anti-LAG-3 arm.

In addition to baseline PD-L1 and LAG-3 expression, we sought to measure tumor burden kinetics longitudinally in the preoperative and postoperative setting as a more accurate indicator of therapeutic efficacy. There are several studies using ctDNA to evaluate MRD in the adjuvant/neoadjuvant immunotherapy space^16,27^, but to the best of our knowledge, our study is one of the first to assess MRD in esophageal cancer patients treated with neoadjuvant immunotherapy combined with CRT. Notably, when evaluating ctDNA after ICI induction, ctDNA detection was indicative of inferior clinical outcomes, as patients with undetectable ctDNA post two cycles of induction ICI had a significantly longer RFS. These findings suggest that a subset of patients may benefit from neoadjuvant immunotherapy alone, and clearance of ctDNA during the neoadjuvant window may be an early predictor of favorable outcomes, which should be evaluated in future studies. Similarly, in the perioperative period, patients with undetectable ctDNA preoperatively had a longer RFS compared to those with detectable ctDNA. Postoperatively, patients with ctDNA MRD positive attained shorter RFS compared to patients that were MRD negative. These findings are consistent with previous studies, where ctDNA MRD positive 4 weeks after surgery confers a significantly higher risk of recurrence and can guide adjuvant chemotherapy treatment decision-making^28,29^. Taken together, our findings suggest that ctDNA status as early as after ICI induction may be critical in enriching for patients most likely to benefit from neoadjuvant ICI and inform treatment escalation or de-escalation, ultimately maximizing therapeutic benefit.

While pCR and MPR have been used as an early indicator of therapeutic efficacy in the neoadjuvant ICI setting, we are increasingly recognizing the clinical and biological heterogeneity of tumors with non-pCR/MPR^16^. Notably, ctDNA status distinguished patients with non-pCR with differential clinical outcomes that were accurately captured by ctDNA kinetics. Strikingly, none of the patients within the non-pCR group that were ctDNA negative after ICI induction recurred compared to patients within the same non-pCR group that were ctDNA positive after ICI. These findings, while requiring further validation, suggest that clinical decision-making for perioperative management may be best informed by ctDNA approaches rather than pathological responses. Linking circulating tumor burden with functional antitumor immune responses, neoantigen-specific T cell responses were a mirror image of ctDNA kinetics, such that ctDNA clearance was associated with the detection and expansion of neoantigen-specific T cells in patients attaining longer RFS and OS. These findings further support the value of ctDNA analyses in capturing functional antitumor immune responses and may allow us to optimally design future neoadjuvant studies using combined blood-based assays to escalate or de-escalate treatment in an attempt to maximize response.

Our study has a number of limitations, mostly related to the small number of patients. As such, the cohort size should be considered when interpreting our work, and larger studies are required to further validate our proof-of-concept findings. In parallel, a sizable fraction of the individuals on this trial had undetectable ctDNA, likely related to the earlier stage of these tumors and the sensitivity of the tumor-agnostic NGS assay employed; it is, therefore, plausible that a bespoke liquid biopsy approach may increase the sensitivity of detection. Tumor-agnostic hybrid capture ctDNA NGS, while readily applicable in the metastatic setting^16,30,31^, may be limited by an assay sensitivity typically in the order of 0.1–0.2% in the early stage setting, resulting in a moderate clinical sensitivity^32^. Tumor-informed NGS approaches that track patient-specific variants have higher analytical sensitivity, however, may be limited by the feasibility of bespoke approaches that require genomic analyses of the tumor followed by patient-specific liquid biopsy assays. The challenge of clinical sensitivity for ctDNA MRD detection, as well as the issues with feasibility of tumor-informed bespoke assays, may be alleviated by genome-wide cell-free DNA (cfDNA) feature integration. Conceptually moving away from single/oligo-mutation capture and toward analyses of millions of cfDNA features from plasma methylation-based approaches or whole genome sequencing (or both) may increase assay sensitivity^33^. Despite the encouraging nature of our findings, larger studies are needed to confirm the association of ctDNA status during neoadjuvant ICI and in the postoperative period with RFS and OS. As a representative example, the ongoing Eastern Cooperative Group (ECOG)/American College of Radiology Imaging Network (ACRIN) phase II/ III EA2174 study is investigating neoadjuvant nivolumab and adjuvant nivolumab with or without ipilimumab in patients with locoregional esophageal E/GEJ cancers treated with trimodality therapy. The dual primary endpoints are pathologic complete response and disease-free survival, and ctDNA assessment is also planned as part of the correlative studies.

In conclusion, neoadjuvant nivolumab plus CRT is an active regimen for patients with operable E/GEJ cancer, resulting in higher pCR and 2-year OS rates compared to historical chemoradiation controls. The addition of relatlimab to nivolumab did result in a higher rate of irAEs than nivolumab plus CRT alone, but promising long-term efficacy suggests that future studies should further evaluate the optimal sequencing of dual ICI when given concurrently with chemoradiation. Interpreting pathological responses at a molecular level, systemic tumor burden regression conferred longer clinical outcomes, bringing ctDNA approaches toward the epicenter of perioperative clinical decision-making. Collectively, our findings suggest that monitoring systemic tumor burden kinetics during neoadjuvant ICI may bring precision in the clinical management of patients with resectable E/GEJ cancer and open a therapeutic window for future intervention.

## Methods

### Study design, eligibility criteria and participants

This is a phase IB, open-label, multi-institution study enrolling patients at Johns Hopkins Sidney Kimmel Comprehensive Cancer Center in Baltimore, MD, Allegheny Health Network in Pittsburgh, PA and Baylor University Medical Center in Dallas, TX (ClinicalTrials.gov registration:NCT03044613, study preregistration date was February 2, 2017). The study protocol and all amendments were approved by the Institutional Review Board (IRB) of Johns Hopkins University (Johns Hopkins Medicine IRB 6) and local institutions (Allegheny Singer Research Institute and Baylor Scott & White Research Institute). A detailed description of all protocol amendments is shown in Supplementary Table 1. This study was conducted in accordance with the Declaration of Helsinki and the international standards of good clinical practice. Written informed consent was provided by all study participants; participants were not compensated. The first and last patients were enrolled in the study on August 23, 2017, and July 1, 2021, respectively. The data lock date was January 25, 2022. Patients aged 18 years and older and with clinical stage II/stage III E/GEJ adenocarcinoma or SCC (American Joint Commission on Cancer (AJCC) seventh edition staging system) were eligible to enroll in the study. All patients had to have surgically resectable disease, ECOG performance status 0–1, adequate organ function and cardiopulmonary status. Sex was determined by self-reporting, both females and males were enrolled in the study and sex was not a stratification criterion. Patients were excluded from the study if they were not candidates for CRT, their esophageal tumor was located in the mid esophagus or higher, they had autoimmune disease or immunodeficiency, or previously received immunotherapy for other disease, or they had active infectious disease requiring ongoing treatment. Patients were excluded from Arm B (relatlimab regimen) if they had any history of myocarditis or uncontrolled or significant cardiovascular disease such as myocardial infarction (MI) or stroke/transient ischemic attack (TIA) within the 6 months before consent, history of two or more MIs or two or more coronary revascularization procedures, or uncontrolled angina within the 3 months before consent. The full eligibility criteria are listed below.

### Inclusion criteria

- Men and women aged ≥18 years old.
- Histologically proven (squamous cell or adenocarcinoma) esophageal or gastroesophageal junction cancer (core biopsy required).
- Stage II/stage III disease as per AJCC staging 7.0—baseline imaging with standard of care fludeoxyglucose F18 (FDG)-positron emission tomography (PET) scan and endoscopic ultrasound within 28 days before registration.
- ECOG performance status 0–1.
- Adequate oral intake/nutritional status without the need for enteral or parenteral feeding during chemoradiation or preoperative period.
- Adequate organ function as follows: leukocytes ≥2,000 mm^−^^3^, absolute neutrophil count ≥1,000 mm^−3^, platelet count ≥100,000 mm^−^^3^, hemoglobin ≥9 g dl^−1^, creatinine ≤2.0 mg dl^−1^, bilirubin (total) within normal institutional limits (except participants with Gilbert syndrome who must have total bilirubin <3.0 mg dl^−1^), AST(SGOT; serum glutamic oxaloacetic transaminase), ALT(SGPT; serum glutamic-pyruvic transaminase) and alkaline phosphatase ≤2.5 times the upper limit of normal, prothrombin time (PT) such that international normalized ratio (INR) is ≤1.5 (or an in-range INR, usually between 2 and 3, if a patient is on a stable dose of therapeutic warfarin and a PTT ≤ upper limit of normal).
- Adequate cardiac function as defined by: no evidence of PR prolongation or atrioventricular (AV) block on baseline electrocardiogram.
- Radiation oncology consultation within 28 days to confirm that disease can be encompassed in the radiotherapy field and that normal tissue constraints can be met.
- Participants must have adequate lung function to permit surgical resection determined by pre-enrollment pulmonary function tests to include diffusing capacity for carbon monoxide (DLCO) as follows: DLCO ≥70% predicted or DLCO <70% but ≥55% with a VO_2_ max ≥10 L min^−1^ kg^−1^ (assessed by cardiopulmonary exercise testing) or 6-min walk test 500 m; participants with a DLCO <55% are excluded from this study. Participants must have a baseline O_2_ saturation by pulse oximetry that is ≥92% both at rest and while walking, off supplemental oxygen.
- Esophagogastrectomies will be performed via a laparotomy and a right thoracotomy with en bloc removal of perigastric, celiac, periesophageal and subcarinal lymph nodes. Esophagogastric reconstruction will be performed above the level of the azygo-caval junction using an end-to-end anastomosis (EEA) stapling device.
- Either a formalin-fixed paraffin block or a minimum of ten 5-micron tissue sections (slides) of tumor biopsy sample must be available for biomarker evaluation from baseline and repeat esophagogastroduodenoscopy (EGD).
- “The effects of nivolumab or nivolumab/relatlimab on the developing human fetus are unknown. For this reason, women of childbearing potential (WOCBP) and men must agree to use adequate contraception (hormonal or barrier method of birth control; abstinence) before study entry and for the duration of study participation and for 5 months after the last dose of nivolumab +/− relatlimab. Should a woman become pregnant or suspect she is pregnant while she or her partner is participating in this study, she should inform her treating physician immediately. Sexually active fertile men must use effective barrier birth control if their partners are WOCBP for 7 months after the last dose of nivolumab +/− relatlimab. WOCBP must have a negative serum or urine pregnancy test (minimum sensitivity 25 IU l^−1^ or equivalent units of HCG) within 2 weeks of registration.”
- Patient understands the study regimen, its requirements, risks and discomforts and is able and willing to sign the informed consent form. Voluntary signed and dated IRB approved written informed consent form in accordance with regulatory and institutional guidelines must be obtained before the performance of any protocol-related procedures that are not part of normal patient care. Participants must be competent to report AEs and understand the drug dosing schedule and use of medications to control AEs.
- (Relatlimab arm only) left ventricular ejection fraction (LVEF) assessment with documented LVEF ≥50% by either transthoracic echocardiogram (TTE) or multigated acquisition (MUGA) scan (TTE preferred test) within 6 months from the first study drug administration.

### Exclusion criteria

- Patient has active, known or suspected autoimmune disease. Participants with vitiligo, type I diabetes mellitus, residual hypothyroidism due to autoimmune thyroiditis only requiring hormone replacement or conditions not expected to recur in the absence of an external trigger are permitted to enroll.
- Esophageal tumors that are located in the mid esophagus or higher that do not involve distal esophagus or GE junction.
- Tumors whose proximal ends are higher than the level of the carina.
- Biopsy proven involvement of supraclavicular lymph nodes.
- Tumors extend 5 cm or more into the stomach.
- Patient has a condition requiring systemic treatment with either corticosteroids (>10 mg daily prednisone equivalent) or other immunosuppressive medications within 14 days of the first dose. Inhaled or topical steroids and adrenal replacement steroid doses are permitted in the absence of active autoimmune disease.
- Participants with previous malignancies (except nonmelanoma skin cancers, in situ bladder, gastric, breast, colon or cervical cancers/dysplasia) are excluded unless a complete remission was achieved at least 1 year before study entry, and no additional therapy (other than adjuvant hormonal therapy for breast cancer) is required or anticipated to be required during the study period.
- Participants with known brain metastasis are excluded from this study. Patients with suspected brain metastasis must have brain imaging (either magnetic resonance imaging or computed tomography (CT) brain with contrast) before enrollment.
- Participants with a history of interstitial lung disease.
- Active systemic infection requiring therapy, positive tests for hepatitis B surface antigen or hepatitis C RNA.
- Known positive history or positive test for human immunodeficiency virus or acquired immunodeficiency syndrome.
- History of allergy to study drug components.
- Women who are pregnant or nursing.
- WOCBP and men with female partners (WOCBP) who are not willing to use contraception.
- Prior therapy with an anti-PD-1, anti-PD-L1, anti-PD-L2 or anti-LAG-3 antibody (or any other antibody targeting T cell coregulatory pathways).
- Underlying medical conditions that, in the Investigator’s opinion, will make the administration of the study drug hazardous or obscure the interpretation of toxicity or adverse events.
- Prisoners or participants who are involuntarily incarcerated or compulsorily detained for treatment of either a psychiatric or physical (for example, infectious disease) illness.
- (Relatlimab arm only) troponin T (TnT) or I (TnI) >2× institutional upper limit of normal (ULN). Participants with TnT or TnI levels between >1 to 2× ULN will be permitted if repeat levels within 24 h are ≤1× ULN. If TnT or TnI levels are >1 to 2× ULN within 24 h, the participant may undergo a cardiac evaluation and be considered for treatment, following a discussion with the medical monitor or designee. When repeat levels within 24 h are not available, a repeat test should be conducted as soon as possible. If TnT or TnI repeat levels beyond 24 h are <2× ULN, the participant may undergo a cardiac evaluation and be considered for treatment, following a discussion with the sponsor medical monitor or designee.
- (Relatlimab arm only) participants must not have a history of myocarditis.
- (Relatlimab arm only) uncontrolled or significant cardiovascular disease including, but not limited to, any of the following: MI or stroke/TIA within the 6 months before the consent; uncontrolled angina within the 3 months before the consent; any history of clinically significant arrhythmias (such as ventricular tachycardia, poorly controlled atrial fibrillation, ventricular fibrillation or torsades de pointes); QTc prolongation >480 ms; history of other clinically significant cardiovascular diseases (that is, cardiomyopathy, congestive heart failure with New York Heart Association functional classification III–IV, pericarditis, significant pericardial effusion, significant coronary stent occlusion, poorly controlled venous thrombosis, etc.); cardiovascular disease-related requirement for daily supplemental oxygen; history of two or more MIs or two or more coronary revascularization procedures.

### Major protocol amendments

This investigator-initiated trial was initially developed to evaluate nivolumab in Arm A and ipilimumab + nivolumab in Arm B. Before the enrollment of any patients in Arm B, the protocol was amended on July 25, 2018, to replace ipilimumab with relatlimab in Arm B to reflect emerging data supporting the evaluation of relatlimab in gastroesophageal cancer (Supplementary Table 1). On December 11, 2019, the protocol was further amended to remove nivolumab and relatlimab during chemoradiation in Arm B (while keeping two cycles of nivolumab and relatlimab as induction before chemoradiation), due to unacceptable toxicity observed in the first nine patients treated in Arm B.

### Treatment and protocol amendments

The following two treatment cohorts were consecutively enrolled: nivolumab + chemoradiation (Arm A) and nivolumab + relatlimab + chemoradiation (Arm B). Patients were enrolled in Arm B after safety and feasibility objectives were met in Arm A. Patients in Arm A (*n* = 16) received nivolumab 240 mg every 2 weeks for two induction cycles, and then three additional doses were given concurrently with chemoradiation for a total of five cycles. Patients enrolled in Arm B (*n* = 16) received nivolumab 240 mg every 2 weeks and relatlimab 80 mg every 2 weeks according to the same schedule. All patients treated in the study had radiation plans prereviewed by a centralized radiation oncology review team, and a more detailed review of each protocol patient was undertaken by a peer review team of thoracic radiation oncologists in the first week of treatment. Nivolumab and relatlimab combined with chemoradiation demonstrated unacceptable toxicity in the first nine patients treated in Arm B. In December 2019, a trial protocol amendment was implemented to remove nivolumab and relatlimab during chemoradiation in Arm B (keeping two cycles of nivolumab and relatlimab as induction before chemoradiation; Supplementary Table 1). Seven patients were subsequently enrolled in Arm B. For the CRT portion of the study, patients were treated with the standard of care regimen of weekly carboplatin (AUC 2) and paclitaxel (50 mg m^−^^2^) combined with radiotherapy at a total dose of 50.4 Gy in 28 fractions. IMRT and volumetric modulated arc therapy (VMAT) technologies were allowed. An Ivor Lewis esophagectomy was planned for 6–8 weeks, but no more than 11 weeks after the last dose of radiotherapy. Dose interruptions for chemotherapy or immunotherapy-induced toxicities were allowed. Adjuvant therapy was allowed at the discretion of the treating physician (in consultation with the principal investigator).

### Assessments and endpoints

The primary endpoint of the study was safety, and the secondary endpoint was feasibility. Exploratory endpoints included OS, RFS, MPR and pCR rates and biomarker analyses. RFS and OS were measured every 3 months before and after surgical resection. Longer follow-up beyond 36 months will continue for both arms as part of the trial design. Safety was assessed by the National Cancer Institute Common Terminology Criteria for Adverse Events version 4.0 and measured through the proportion of evaluable patients whose worst adverse events of interest occurred within 100 days after the last dose of nivolumab or within 30 days after surgery, whichever was longer. The proportion of any grade 3 or 4 treatment-related pneumonitis and acute respiratory failure, as well as that of any treatment-related grade 5 AE, were monitored continuously based on prespecified Bayesian monitoring rules. We assessed the feasibility of single agent IO, and combination IO–IO neoadjuvant administration as induction was given concurrently with chemoradiation. Feasibility was assessed through the proportion of eligible patients who proceeded to surgery without substantial delay (more than 11 weeks) due to treatment-related reasons.

Radiological assessment was performed according to the Response Evaluation Criteria in Solid Tumors (version 1.1)^34^ at baseline (CT and PET/CT), before surgery (PET/CT) and per standard of care after surgery until 5 years (generally every 3 months for at least the first year, by CT). Patients enrolled in this study were required to have pretreatment primary tumor biopsy material available for diagnosis. Repeat EGD biopsies were obtained if pre-existing material was inadequate.

### Pathological response assessment and immunohistochemistry

Pathological response was assessed semi-quantitatively using a modified Ryan scheme^35^, as recommended by the CAP, using the following categories to assign tumor regression scores/grades: no viable cancer cells (complete response; grade 0); single cells or rare small groups of cancer cells (near complete response; grade 1); residual cancer with evident tumor regression, but more than single cells or rare small groups of cancer cells (partial response; grade 2); extensive residual cancer with no evident tumor regression (poor or no response; grade 3). When available, all tumor/tumor bed slides were reviewed for evaluation of pathological treatment response, but otherwise, at least two slides representing a full cross-section of the tumor bed were reviewed. Percent residual viable tumor was also evaluated in increments of five (5%) based on a percentage of tumor bed occupied by tumor cells. Tumor bed was identified by a combination of features including scar/fibrosis, inflammatory response, neovascularization, foamy macrophage aggregates, acellular mucin and/or calcifications.

A representative formalin-fixed, paraffin-embedded section of tumor from baseline biopsies (*n* = 29) was stained for PD-L1, while immunohistochemistry for PD-L1, DNA MMR proteins (MLH1, PMS2, MSH2 and MSH6) and HER2 was performed in resected specimens with adequate tumor tissue (*n* = 15, obtained at the time of surgery after immuno-chemoradiation). PD-L1 staining was performed using clone 22C3 (Agilent) and run on Roche/Ventana Benchmark Ultra with the Optiview detection kit. HER2 staining was performed using clone 4B5 (Roche) and run on Roche/Ventana Benchmark Ultra with the Ultraview detection kit. MLH1 (M1; Roche), MSH2 (G219-1129; Roche) and MSH6 (SP93; Roche) staining were performed on Roche/Ventana Benchmark Ultra with the Ultraview detection kit. PMS2 staining (A16-4 clone; Roche) was run on Roche/Ventana Benchmark Ultra with the Ultraview detection kit and Optiv amplification kit. PD-L1 staining was scored using previously published ‘CPS’ defined as the total number of tumor cells and immune cells expressing membranous (tumor) or membranous and cytoplasmic (immune cells) PD-L1 divided by the total number of tumor cells and multiplied by 100 (ref.^36^). A minimum of 100 tumor cells were required for PD-L1 evaluation. PD-L1 was evaluated in 15 resected specimens with adequate tumor tissue for testing. HER2 staining was scored as 0, 1+, 2+ or 3+ using criteria recommended by the CAP for scoring HER2 expression by immunohistochemistry in gastric and gastroesophageal junction carcinomas and published in the ToGA trial^37^. Briefly, the criteria were as follows: 0 (no reactivity or membranous reactivity in <10% of cancer cells), 1+ (faint or barely perceptible membranous reactivity in ≥10% of cancer cells; cells are reactive only in part of their membrane), 2+ (weak to moderate complete, basolateral or lateral membranous reactivity in ≥10% of tumor cells), 3+ (strong complete, basolateral or lateral membranous reactivity in ≥10% of cancer cells). HER2 expression was evaluated in 15 resected specimens with adequate tumor tissue for testing. Specimens were considered MMR proficient if nuclear expression of all four MMR proteins was present by immunohistochemistry and loss of specific MMR proteins was recorded when loss of nuclear expression was seen in the tumor cells with intact internal control labeling in normal tissues. MMR proficiency was evaluated in 13 resected specimens with adequate tumor tissue. Two resected tumors (patients CGES37 and CGES19) showed MLH1 and PMS2 expression loss. To confirm MMR status in these cases, we repeated MLH1 and PMS2 immunohistochemistry in both baseline and resected tumors (serial sections from the resected specimens previously stained) and performed orthogonal validation by assessment of microsatellite instability derived from whole-exome sequencing (WES) of the baseline tumors. These analyses showed cleanly intact MLH1 and PMS2 labeling in baseline biopsies for both patients, while repeat staining of the resected specimens showed <10% tumor cells with MLH1 loss for case CGES37 and failure of both stains (no labeling in internal control normal tissue or tumor) for case CGES19.

### WES analyses

Matched tumor/normal WES was performed in baseline tumors for patients CGES19 and CGES37, to assess microsatellite instability. Briefly, DNA was macrodissected from formalin-fixed paraffin-embedded (FFPE) tumor tissue using the Qiagen DNA FFPE tissue kit, and DNA was isolated from matched WBC using the Qiagen DNA Blood Mini kit (Qiagen). Both tumor and matched WBC DNA were sheared to a target DNA fragment size of 200 bp using Covaris-focused ultrasonication (Covaris). Genomic libraries were prepared, and sequentially hybrid captures of exonic regions using SureSelect XT Human All Exon V4 probes (Agilent Technologies) were prepared as previously described^38^. Captured libraries were then sequenced on Illumina HiSeq 2500 (Illumina). Somatic mutations were identified using Strelka^39^. To evaluate microsatellite instability, we applied MANTIS^40^ to WES data. Briefly, the microsatellite loci within the reference genome (hg19) were identified, and a step-wise difference (DIF) metric was used in each locus to compare the normalized read counts supporting repeats of a given length between the tumor and matched normal sample after exclusion of reads and loci below a predetermined quality threshold. The average of the locus instability scores was calculated and used as an aggregate measure of microsatellite instability in the sample. For each tumor–normal pair, the step-wise difference metric was compared to the decision threshold recommended by MANTIS to make MSI calls. Both tumors were characterized as microsatellite stable, and together with the immunohistochemistry data, these findings support that both tumors were MMR proficient.

### Gene expression assessment

Gene expression of 770 genes was evaluated using NanoString’s fluorescence-based direct digital detection chemistry and nSolver analyses (NanoString Technologies). Briefly, RNA was isolated from pretherapy fresh frozen tissue biopsies using the Qiagen RNeasy Mini kits (Qiagen). Isolated RNA was then hybridized to probes from NanoString’s nCounter PanCancer IO 360 gene expression panel that includes 770 unique genes associated with immune responses, immune escape, tumor signatures and the tumor microenvironment, as well as housekeeping genes for normalization (NanoString Technologies). Each hybridization probe set included both positive and negative control probes. The positive control probes were designed to bind to synthetic controls spiked into the panel and used to determine assay performance. The negative control probes were probes whose target is not expected to be in biological samples and were used to set background thresholds. Each experimental set also included a panel standard for run-to-run comparisons. Using the nCounter MAX/FLEX system, hybridized RNA was first loaded onto the prep station for posthybridization purification and then onto the digital analyzer for quantification of gene expression. Gene expression analyses were performed using NanoString nSolver software. Raw gene expression counts from the digital analyzer were imported into nSolver. Normalization of counts was performed based on the geNorm algorithm, which selects the housekeeping genes in the panel that minimize the pairwise variation statistic (Supplementary Table 7).

### Circulating cell-free tumor DNA analyses

Serial blood samples were collected before therapy initiation, on day 1 of cycles 2 and 3, immediately before surgical resection, and once postoperatively between 3 and 12 weeks after surgical resection. The postoperative time point in the 3–12-week window was collected with the intent to capture MRD after curative-intent surgery as opposed to longitudinal monitoring. We used targeted error-correction sequencing (TEC-seq) to perform high-coverage NGS on 173 serial plasma samples and matched baseline WBC-derived DNA from 32 patients from Arms A and B as previously described^15,41^ (Supplementary Tables 8–10). Briefly, cfDNA was isolated using the Qiagen Circulating Nucleic Acids kit (Qiagen). Genomic libraries were prepared, followed by targeted capture using a custom set of hybridization probes (Personal Genome Diagnostics) as previously described^15,41^. The analytical performance of the hybrid capture NGS assays has been previously described^15,30,31,42^, with an overall specificity of >99% and sensitivity of >95% sensitivity for the detection of alterations with an MAF of 0.25–1.0%. Captured libraries were sequenced using 100 bp paired-end runs on the Illumina HiSeq 2500 or NextSeq 550 instruments (Illumina; Supplementary Table 9). Matched WBC DNA TEC-seq was performed to filter out plasma variants related to CH. A summary of the genomic alterations detected in cfDNA alongside their origin is shown in Supplementary Table 10.

Somatic variants were identified across the targeted regions using VariantDx, and variant origin was determined using a tumor-agnostic WBC-informed approach^30^. Germline and CH variants were filtered out using matched WBC DNA sequencing. Taking into consideration buffy coat contamination by circulating tumor cells, which would result in the classification of tumor-derived variant as CH-derived, we used information about variant occurrences in the COSMIC somatic mutation registry to determine variant origin. Variants detected in plasma were cross-referenced with COSMIC to annotate cancer hotspot alterations using OpenCRAVAT^43^. Hotspots were defined as ≥25 COSMIC occurrences. Variants that are classified as nonhotspots and have a variant allele fraction ≥ 25% in all plasma and the WBC samples from the same patient were classified as germline. All hotspot and remaining nongermline variants were further examined to determine variant origin. Plasma variants with a super mutant count of ≥3 were classified as CH-derived, and variants with a super mutant count of 0 were classified as tumor-derived. Variants with a super mutant count of ≥1 were analyzed further to eliminate CH-derived variants that are below the TEC-seq level of detection. Nonhotspot variants were classified as CH-derived, and hotspot variants were further examined using COSMIC occurrences. Variants with occurrences in hematologic and lymphoid malignancies ≥10% of all occurrences were classified as CH-derived and ones <10% were classified as tumor-derived. All variants assigned as tumor-derived were visually inspected using the Integrative Genomic Viewer and considered in our analyses.

### Functional T cell assays

Neoantigen-specific T cells were detected in peripheral blood using the Mutation-Associated Neoantigen Functional Expansion of Specific T Cells (MANAFEST) assay as described previously^17,18^, with minor modifications (Supplementary Tables 12–18). This approach combines ex vivo T cell culture and peptide stimulation with T cell receptor (TCR) sequencing to identify significant and specific T cell clonotypic expansions. From each patient, 40–60 putative neoantigens were selected based on predicted major histocompatibility complex (MHC) class I affinity and expression in the relevant tumor type and were synthesized (JPT Peptide Technologies). Peptides were combined into pools of 4–6 peptides per pool for a total of ~10 pools per patient. T cells were isolated from peripheral blood mononuclear cells (PBMC) by negative selection (EasySep; STEMCELL Technologies) on day 0 and cultured as previously reported^17,18^. TCR sequencing of extracted DNA from cultured CD8+ cells was performed by the Johns Hopkins Fest and TCR Immunogenomics Core Facility (FTIC) using the Adaptive Biotechnologies hsTCRB Kit using survey-level sequencing (Adaptive Biotechnologies). Nonproductive TCR sequences were eliminated and aligned to obtain only the complementary-determining region 3 (CDR3) region. Sequences not beginning with C or ending with F or W and having less than seven amino acids were eliminated. Processed data files were analyzed using the publicly available MANAFEST analysis web application (http://www.stat-apps.onc.jhmi.edu/FEST) to define neoantigen-specific T cell clonotypes.

### Statistical analyses

For each neoadjuvant regimen, we aimed to enroll 16 evaluable patients into Arm A and 16 patients into Arm B. Evaluable patients were those who received at least one dose of neoadjuvant nivolumab or nivolumab/relatlimab administration and completed toxicity follow-up through 100 days after the last dose of nivolumab. Patients who lost to follow-up within 100 days after the last dose of nivolumab are not considered evaluable. Based on historical data^20^, we assumed the rate of grade 3 or 4 treatment-related pneumonitis and acute respiratory failure in the regimen of chemoradiation and surgery alone is about 9%. Therefore, to minimize the risks of adding nivolumab or nivolumab/relatlimab as neoadjuvant therapy, safety was monitored by a Bayesian stopping rule for the rate of grade 3 or 4 treatment-related pneumonitis and acute respiratory failure greater than 30% (three times of baseline toxicity rate). Specifically, the Bayesian toxicity monitoring rule would suspend the accrual anytime if the posterior probability of grade 3 or 4 treatment-related pneumonitis and acute respiratory failure being larger than 30% is 70% or higher. We assumed a priori that the experimental regimens had an average risk of around 25% and there is a 34% chance that the risk will be 30% or higher. At any time if the stopping criterion was met, accrual to the trial would be temporarily suspended and the principal investigator and study team will review the toxicity data and recommend either modification or termination of the trial. The Bayesian toxicity monitoring rule also would suspend accrual anytime if the posterior probability of treatment-related grade 5 adverse events was larger than 10%. We assumed a priori that the experimental regimens had an average risk of 5% and there is about a 14% chance that the risk will be 10% or higher.

To minimize the potential risks exposed to patients, the safety and feasibility-related analyses for Arm A were conducted before initiating accruals for Arm B. Adverse events for each regimen were tabulated by type, grade and attribution of adverse event. In addition, the proportions of grade 3 or 4 treatment-related pneumonitis and acute respiratory failure, treatment-related grade 5 AE and patients with surgery without substantial delays were reported along with exact binomial 95% CIs. To preliminarily assess the efficacy of the experimental regimen, the pCR rate was estimated among all evaluable patients, and 95% exact CI was provided. RFS was defined as the time from treatment initiation to disease recurrence or death due to any cause, whichever occurred first. OS was defined as the time from treatment initiation to death due to any cause. Patients were censored if no RFS or OS event occurred by the last follow-up. Both RFS and OS were analyzed as time-to-event data, that is, the respective rates at different time points (for example, every 6 months) were estimated using the Kaplan–Meier method, and the associated point-wise CI was calculated using the Greenwood formula with log–log transformation. Given the nature of the single-arm, phase II design for each cohort, the study was not designed to differentiate between intention-to-treat and per-protocol analysis.

For PD-L1 expression and ctDNA analyses, Fisher’s exact test was used for categorical analyses, and for nonparametric comparisons, the Wilcoxon rank-sum test was used. For survival analyses, RFS and OS were analyzed as time-to-event data with median point estimates calculated using the Kaplan–Meier method, and survival curves were compared using the log-rank test. Statistical analyses were performed in R version 3.6.1.

### Reporting summary

Further information on research design is available in the Nature Portfolio Reporting Summary linked to this article.

## Online content

Any methods, additional references, Nature Portfolio reporting summaries, source data, extended data, supplementary information, acknowledgements, peer review information; details of author contributions and competing interests; and statements of data and code availability are available at 10.1038/s41591-024-02877-z.

### Supplementary information

Supplementary Information Study protocol.

Reporting Summary

Supplementary Tables Supplementary Table 1: Summary of protocol amendments. Supplementary Table 2: Treatment-related adverse events. Supplementary Table 3: Pathological characteristics and responses. Supplementary Table 4: Summary of PD-L1, HER2 and MMR immunohistochemistry. Supplementary Table 5: PD-L1 CPS per histology. Supplementary Table 6: PD-L1 CPS comparisons. Supplementary Table 7: Normalized LAG-3 expression. Supplementary Table 8: Summary of sequenced plasma samples. Supplementary Table 9: Summary of targeted error-correction sequencing characteristics. Supplementary Table 10: Sequence alterations detected in cfDNA. Supplementary Table 11: ctDNA assessment throughout the duration of the study. Supplementary Table 12: Neoantigen-specific TCR productive frequencies from the MANAFEST assay for patient CGES13*. Supplementary Table 13: Neoantigen-specific TCR productive frequencies from the MANAFEST assay for patient CGES2*. Supplementary Table 14: Neoantigen-specific TCR productive frequencies from the MANAFEST assay for patient CGES11*. Supplementary Table 15: Neoantigen-specific TCR productive frequencies from the MANAFEST assay for patient CGES3*. Supplementary Table 16: Neoantigen-specific TCR productive frequencies from the MANAFEST assay for patient CGES5*. Supplementary Table 17: Neoantigen-specific TCR productive frequencies from the MANAFEST assay for patient CGES12*. Supplementary Table 18: Neoantigen-specific TCR productive frequencies from the MANAFEST assay for patient CGES27*.

## Data Availability

NGS data from plasma and matched WBC DNA are deposited and can be retrieved from the European Genome-Phenome Archive (EGA accession EGAS00001007299). To retrieve the dataset, access can be requested through the EGA portal and upon completion of a data use agreement.
