# Supplementary material for: Neoadjuvant nivolumab or nivolumab plus LAG-3 inhibitor relatlimab in resectable esophageal/gastroesophageal junction cancer: a phase Ib trial and ctDNA analyses
Source: Nat Med. 2024 Mar 19;30(4):1023–34. doi: 10.1038/s41591-024-02877-z (PMC11031406; doi:10.1038/s41591-024-02877-z)
Supplement: Supplementary file 1 — Study protocol. [file 41591_2024_2877_MOESM1_ESM.pdf]

nature medicine

Article https://doi.org/10.1038/s41591-024-02877-z

Neoadjuvant nivolumab or nivolumab plus LAG-3 inhibitor relatlimab in resectable esophageal/gastroesophageal junction cancer: a phase Ib trial and ctDNA analyses

In the format provided by the authors and unedited

Study Title: Phase IB trial of induction nivolumab or nivolumab/ relatlimab prior to concurrent chemoradiation in patients with operable stage II/III esophageal/ gastroesophageal junction cancer

JHMI Protocol ID number/: IRB00122689/ J1714
BMS Number: CA209-906

Principal Investigator
Vincent Lam
Sidney Kimmel Comprehensive Cancer Center at Johns Hopkins
201 North Broadway
Suite #8125
Baltimore, MD 21287
Phone: 410-550-5358

Email: vklam@jhmi.edu

Participating Institution
Allegheny Health Network
Blaire Job, Blair.Jobe@ahn.org , Site Principal Investigator
Ali Zaidi, ali.zaidi@ahn.org, Co-Investigator

Charles A Sammons Cancer Center
Ronan Kelly, Ronan.Kelly@BSWHealth.org, Co-Investigator

Coordinating Center Lead Coordinator
Elisabeth Prophet
Telephone: 443-287-2021
e-mail: eprophe1@jhmi.edu

Coordinating Center:
Johns Hopkins University School of Medicine
201 N Broadway
Virgah 9th Floor
Baltimore, MD 21287
Telephone: 443-287-9377
Fax: 410-502-9933
e-mail: crocc@jhmi.edu

Supported by:
Bristol Myers Squibb

Supplied Agents:
Nivolumab (BMS-936558); (IND# 133,592) BMS Inc.
Relatlimab (BMS-986016)

SYNOPSIS

Protocol Title: Phase IB trial of neoadjuvant nivolumab or nivolumab/ relatlimab prior to concurrent chemoradiation in patients with operable stage II/III esophageal/ gastroesophageal junction cancer

Investigational Product(s), Dose and Mode of Administration, Duration of Treatment with Investigational Product(s):

- Arm A: Nivolumab 240mg administered IV over 30 minutes every 2 weeks for 2 cycles and then standard of care chemoradiation (weekly carboplatin/paclitaxel and concurrent radiation).
- Arm B: Nivolumab 240mg administered IV over 30 minutes followed by relatlimab 80mg administered IV over 60 minutes on Day 1 every 2 weeks for 2 cycles and then standard of care chemoradiation (weekly carboplatin/paclitaxel and concurrent radiation).

Study Phase: Phase IB

Research Hypothesis: Anti-PD-1 (nivolumab) or Anti-PD1/Anti LAG-3- (relaltimab) administration in the pre-operative setting and chemoradiation will be safe and feasible in patients with resectable distal esophageal/gastroesophageal junction cancer and will change cellular and molecular characteristics of the tumor microenvironment that will improve survival.

Objectives:

Primary Objective

To investigate the safety of induction nivolumab or nivolumab/ relatlimab administration prior to concurrent chemoradiation in subjects with resectable stage II/III gastro-esophageal junction cancer.

Secondary Objectives

- To investigate the feasibility of induction nivolumab or nivolumab/relatlimab prior to concurrent chemoradiation administration in subject's with stage II/III esophageal and gastro-esophageal junction cancer.
- To determine the pathological complete response rate in patients treated with induction checkpoint inhibition followed by chemo-radiation plus nivolumab +/- relatlimab prior to surgical resection.

- To explore the association between nivolumab +/- relatlimab exposure and selected pharmacodynamics markers in the peripheral blood and in the tumor microenvironment, including measurement of PD-1 receptor occupancy on tumor infiltrating lymphocytes.

- To measure changes in expression of selected immune markers including changes in the quality and quantity of tumor infiltrating lymphocytes and the T effector to T-Reg ratio compared to baseline, in the blood, primary tumor tissue and draining lymph nodes

- To assess recurrence-free survival in patients receiving neoadjuvant checkpoint inhibitors.

- To assess overall survival in patients receiving neoadjuvant checkpoint inhibitors.

Study Design

This is a Phase IB study assessing the safety of 2 cycles of induction (Arm A) nivolumab or (Arm B) 2 cycles of induction nivolumab plus relatlimab prior to concurrent chemoradiation before surgical resection in operable stage II/III esophageal/gastroesophageal junction cancer.

Approximately 32 patients will be enrolled on study with 16 enrolled on Arm A and if no unexpected toxicities then an additional 16 patients will be enrolled on Arm B.

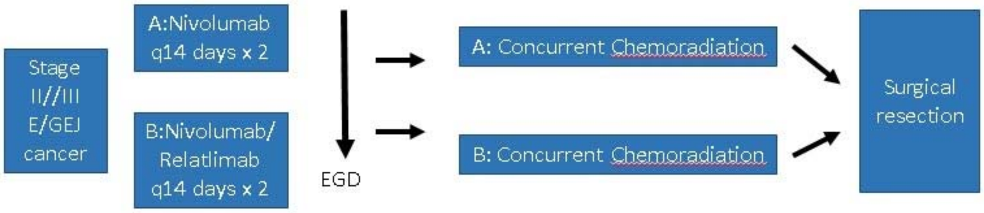

Standard of care chemoradiation – CROSS regimen of weekly carboplatin (AUC2) plus paclitaxel (50mg/m2) x 5 weeks

Primary objective
-safety and tolerability
Secondary objective:
-Pathologic complete response rate, DCR, OS, correlative studies

Study Population:

Subjects must meet all eligibility criteria. The key inclusion and exclusion criteria are as follows:

Key Inclusion Criteria:

- All subjects must have stage II/III distal esophageal or gastroesophageal junction carcinomas that are considered surgically operable
- Subjects must be previously untreated

- ECOG performance status of 0 or 1

Tumor tissue must be available for correlative studies

Key Exclusion Criteria:

- Stage IV disease
- Considered too high risk for surgery based on standard of care surgical guidelines

Study Assessments:

The primary endpoint of this study is safety. Key secondary and exploratory endpoints include assessing the pathological complete response rate and measuring changes in expression of selected immune markers

Statistical Considerations:

This study is a phase 1b trial evaluating the safety and feasibility of up to two neoadjuvant immunotherapy regimens (Nivolumab alone or Nivolumab+ Relatlimab) for operable stage II/III esophageal/gastroesphageal junction cancer. Safety and feasibility will be monitored continuously throughout the study.

The primary endpoint is the safety of neoadjuvant immunotherapy administration. Toxicity will be assessed by CTCAE version 4.0. Safety is measured through the proportion of evaluable patients whose worst adverse events of interest occurred within 100 days after the last dose of last dose of Nivolumab or within 30 days after surgery, whichever is longer. Adverse events of interest include any grade 3 or 4 treatment-related (definitely, probably or possibly) pneumonitis and acute respiratory failure. In addition, we will closely and continuously monitor any treatment-related grade 5 AE.

The secondary endpoint is the feasibility of neoadjuvant immunotherapy administration. Feasibility is assessed through the proportion of eligible patients who proceed to surgery without substantial delay (more than 11 weeks) due to treatment-related reasons.

The exploratory endpoints include:

- Pathological complete response
- Selected pharmacodynamics markers
- Selected immune markers
- Recurrence-free survival, defined as the time from treatment initiation to disease recurrence or death due to any cause, whichever occurs first.
- Overall survival, defined as the time from treatment initiation to death due to any cause.

Sample size and Monitoring Plans

For each neoadjuvant regimen, we aim to accrue 16 evaluable patients. Evaluable patients are those who receive at least one dose of neo-adjuvant nivolumab (or nivolumab+ relatlimab) administration and have complete toxicity follow-up through 100 days after the last dose of nivolumab. Patients who lost to follow up within 100 days after the last dose of nivolumab are not considered evaluable.

Early Stopping Plan for Safety

Based on the data from CROSS trial and a literature search, we assume the rate of grade 3 or 4 treatment-related pneumonitis and acute respiratory failure in the regimen of chemo-radiation and surgery alone is about 9%. Therefore, to minimize the risks of adding nivolumab (or nivolumab+ Relatlimab) as neo-adjuvant therapy, safety will be monitored by a Bayesian stopping rule for the rate of grade 3 or 4 treatment-related pneumonitis and acute respiratory failure greater than 30% (three times of baseline toxicity rate). Specifically, the Bayesian toxicity monitoring rule that suspends the accrual anytime if the posterior probability of grade 3 or 4 treatment-related pneumonitis and acute respiratory failure being larger than 30% is 70% or higher. We assume a priori that the experimental regimens has an average risk around 25% and there is about 34% chance that the risk will be 25% or higher. This corresponds to a Beta(1,3) prior distribution. Table 12.1 summarizes the continuous stopping rule for the 16 evaluable patients for each regimen. For example, if 3 patients out of the first 6 or 4 out of the first 7 evaluable patients experience grade 3 or 4 treatment-related pneumonitis and acute respiratory failure, we will stop accrual. The accrual would resume only if the following Bayesian stopping rule being used is not met. At any time if the stopping criterion is met, accrual to the trial will be temporarily suspended and the principle investigators and study team will review the toxicity data and recommend either modification or termination of the trial.

Table 12.1 Stopping rule for safety

# patients with AE	2	3	4	5	6	7
Out of total # evaluable patients	2-3	4-6	7-9	10-12	13-15	16

Table 12.2 summarizes the operating characteristics based on 5,000 simulations with 16 evaluable patients in terms of how frequent the study would stop based on the stopping rule under different hypothetical toxicity rates, as well as the average sample sizes.

Table 12.2 Operating characteristics of the stopping rule for safety

Underlying risk	0.15	0.20	0.25	0.30	0.35	0.40	0.50
% of time study stops	7%	15.2%	27.5%	42.1%	58.5%	70.9%	89.6%
Expected sample size	15.4	14.7	13.8	12.6	11.2	10.2	8.1

All Grade 3 and Grade 4 immune-related toxicities, with some exceptions (e.g., easily correctable endocrinopathies) and for Grade 3 and Grade 4 hematologic and non-hematologic toxicities, will also be monitored closely and continuously by the study team and investigators. Excessive occurrences (qualitatively comparing to what would be expected with standard of care chemoradiation) may also lead to early stopping.

Early Stopping Plan for Grade 5 AEs

We will also evaluate the rate of treatment-related grade 5 AE if it's greater than 10%. Specifically, the Bayesian toxicity monitoring rule that suspends the accrual anytime if the posterior probability of treatment-related grade 5 AE being larger than 10% is 70% or higher. We assume a priori that the experimental regimens has an average risk 5% and there is about 14% chance that the risk will be 10% or higher. This corresponds to a Beta(0.1,1.9) prior distribution. Table 12.3 summarizes the continuous stopping rule for the 16 evaluable patients for each regimen.

Table 12.3 Stopping rule for safety

# patients with grade 5 AE	1	2	3
Out of total # evaluable patients	1-3	4-10	11-16

Table 12.4 summarizes the operating characteristics based on 5,000 simulations with 16 evaluable patients in terms of how frequent the study would stop based on the stopping rule under different hypothetical grade 5 AE rates, as well as the average sample sizes.

Table 12.4 Operating characteristics of the stopping rule for grade 5 AE

Underlying risk	0.04	0.08	0.10	0.12	0.16	0.20
% of time study stops	6.4%	21.5%	32.2%	38.8%	57.5%	72.4%
Expected sample size	15.5	14.4	13.6	13.1	11.6	10.4

Early Stopping Plan for Feasibility

The feasibility of neoadjuvant nivolumab (or nivolumab+ relatlimab) will be based on the proportion of patients proceeding to surgery without substantial treatment related delays. Recovery from standard of care chemoradiation is different for each patient. For safety reasons, it is standard of care for surgeons to evaluate patients on a case by case basis. Based on our Johns Hopkins institutional experience, patients undergo surgery (Ivor-Lewis esophagectomy) in a range of 7-11 weeks following chemoradiation with no mortality recorded within 90 days after surgery.

Therefore, in this study a treatment related delay will be considered "substantial" if it is greater than 11 weeks following chemoradiation. We would consider the experimental regimen is "infeasible" if the probability of not proceeding to surgery as planned and delayed is more than .25 (with more than 80% posterior probability), i.e., the probability of proceeding to surgery as planned is less than 75%. We assume at most 10% of patients will have their surgery delayed and there is about 12% chance that the risk will be 25% or more. This corresponds to a Beta(0.5,4.5) prior distribution. Table 12.5 summarizes the continuous stopping rule for the 16 evaluable patients for each regimen. The feasibility stopping rule calls for the study to be paused for a review if the number of patients successfully proceeding to surgery is too low, starting from the 6th evaluable-patient.

Table 12.5 Stopping rule for feasibility

# patients with surgery delayed	4	5	6	7
Out of total # evaluable patients	6-7	8-10	11-13	14-16

Table 12.6 summarizes the operating characteristics based on 5,000 simulations with 16 evaluable patients in terms of how frequent the study would stop based on the stopping rule under different hypothetical feasibility rates, as well as the average sample sizes.

Table 12.6 Operating characteristics of the stopping rule for feasibility

Underlying feasibility	0.65	0.7	0.75	0.8	0.85	0.9
% of time study stops	41%	26.5%	14.1%	6.1%	2%	0.5%
Expected sample size	13.2	14.3	15	15.6	15.8	16

12.3 Statistical Analysis Plans

To minimize the potential risks exposed to patients, the safety and feasibility related analyses for Arm A will be conducted prior to initiating accruals for Arm B.

Adverse events for each regimen will be tabulated by type, grade, and attribution of adverse event. In addition, the proportions of grade 3 or 4 treatment-related pneumonitis and acute respiratory failure, treatment-related grade 5 AE, and patients with surgery without substantial delays will be reported along with exact binomial 95% confidence intervals.

For all exploratory endpoints, descriptive analysis but no formal hypothesis testing will be performed given the nature of exploratory analysis. To preliminarily assess the efficacy of the experimental regimen, pathological complete response rate will be estimated among all evaluable patients, and 95% exact confidence interval will be provided. Recurrence-free survival (RFS) is defined as the time from treatment initiation to disease recurrence or death due to any cause, whichever occurs first. Overall survival (OS) is defined as the time from treatment initiation to death due to any cause. Patients are censored if no RFS or OS event occurs by the last follow-up. Both recurrence-free survival and overall survival will be analyzed as time-to-event data, i.e., the respective rates at different time-points, e.g., every 6 months, will be estimated using Kaplan-Meier method, and the associated point-wise confidence interval will be calculated using Greenwood formula with log-log transformation. Parameters of pharmacodynamics markers and immune markers will be summarized with descriptive statistics. These summaries will be computed for each evaluable patient at multiple timepoints before and after neoadjuvant regimen is administered. Plots will be used to show the changes in immune response over time both for each individual. For each patient, comparisons in the pre and post-nivolumab responses will be compared using paired t-tests (or Wilcoxon signed rank tests if appropriate) for continuous variables and McNemar's test for dichotomous or categorical variables. Associations between immune responses will be explored graphically (e.g. scatterplots, boxplots) and numerically (e.g. correlations, $\chi 2$ tests).

TABLE OF CONTENTS

TITLE PAGE **1**

SYNOPSIS **3**

TABLE OF CONTENTS **10**

1. INTRODUCTION AND STUDY RATIONALE **13**

1.1 Study Rationale 13
1.2 Research hypothesis 22
1.3 Objectives 22
1.4 Product Development Background 23
1.5 Overall Risk/Benefit Assessment 26

2. ETHICAL CONSIDERATIONS **28**

2.1 Good Clinical Practice 28
2.2 Institutional Review Board/Independent Ethics Committee 28
2.3 Informed Consent 29

3. INVESTIGATIONAL PLAN **30**

3.1 Study Design and Duration 30
3.2 Post-operative Treatment of Participants 32

3.3 Evaluation of Peri-operative Safety 33

4. STUDY POPULATION **33**

4.1 Inclusion Criteria 33
4.2 Exclusion Criteria 34
4.3 Inclusion of Genders and Minorities 36
4.4 Concomitant Treatments 36
4.5 Discontinuation of Subjects following Treatment with Study Drug 37
4.6 Post-Study Drug Study Follow Up 38
4.7 Withdrawal of Consent 38
4.8 Lost to Follow-up 38

5. REGISTRATION PROCEDURES **39**
5.1 General Guidelines **39**
5.2 Registration Process **39**

6. STUDY DRUGS-PHARMACOLOGY SAFETY AND ADMINISTRATION OF NIVOLUMAB AND RELATLIMAB **40**

6.1 Investigational Product 40
6.2 Storage of Study Drug 40
6.3 Nivolumab and Relatlimab Dose and Schedule 41
6.4 Dose Delay Criteria for Nivolumab and/or RelatlimabTherapy 43

6.5 Dose Reductions for Nivolumab or Relatlimab 44
6.6 Criteria for Resuming Treatment for Nivolumab or Relatlimab 44
6.7 Criteria for Nivolumab and/or Relatlimab Treatment Discontinuation 45
6.8 Management Algorithms for Immuno-oncology Agents 46
6.9 Treatment of Nivolumab- or Relatlimab -Related Infusion Reactions 47
6.10 Chemotherapy Dosing - Carboplatin and Paclitaxel Dose and Schedule 48
6.11 Radiation Dosing and Schedule 48
6.12 Treatment Compliance 50
6.13 Destruction of Study Drug 50

7. STUDY ASSESSMENTS AND PROCEDURES 50

8. SAFETY ASSESSMENTS 54

8.1 Screening Safety Assessments 54
8.2 On-Treatment Safety Assessments 54
8.3 Follow-Up Safety Assessments 55
8.4 Imaging Assessment for the Study 55
8.5 Efficacy Assessments 55

9. EXPLORATORY IMMUNOLOGIC STUDIES 55

9.1 Immunologic Correlates 55
9.2 Tumor Tissue Samples 56
9.3 Blood Samples 56
9.4 Stool Samples 57

9.5 Specimen Handling, Transportation, Storage and Processing (Appendix D) 57
9.6 Methods of Analysis 57
9.7 Leftover Samples 60
9.8 Additional Information 60

10. ADVERSE EVENTS 60

10.1 GENERAL 60

10.2 Definitions 61
10.3 Serious Adverse Event Collection and Reporting 64
10.4 Non-Serious Adverse Effects 67

11. DATA AND SAFETY MONITORING 69

11.1 Data Management 69
11.2 Multicenter Guidelines 70
11.3 Meetings 71
11.4 Monitoring 71

12. ADMINISTRATIVE PROCEDURES 71

12.1 Protocol Amendments 71

12.2 Informed Consent 71
12.3 Ethics and Good Clinical Practice 72
12.4 Regulatory Authorities 72

13. STATISTICAL CONSIDERATIONS 73

13.1 Study Design and Endpoints 73
13.2 Sample Size and Monitoring Plans 73
13.3 Statistical Analysis Plans 76

APPENDIX A: TNM STAGING SYSTEM FOR ESOPHAGEAL CANCER (7TH EDITION) 78
APPENDIX B: ECOG PERFORMANCE STATUS SCALE 80
APPENDIX C: GUIDELINES FOR TISSUE BANKING PROCESS 81
APPENDIX D: MANAGEMENT ALGORITHMS 83
APPENDIX E: BASELINE SOCIODEMOGRAPHIC CHARACTERISTICS QUESTIONNAIRE 91

APPENDIX F: FOLLOWUP SOCIODEMOGRAPHIC CHARACTERISTICS QUESTIONNAIRE 99

REFERENCES 102

1. Introduction and Study Rationale

1.1 Study Rationale

1.1.1 The current treatment of stage II/III esophageal/gastroesophageal cancer

Different strategies for the management of locally advanced esophageal and gastroesophageal junction carcinomas (E/GEJ) have been investigated over the last two decades but recent data has suggested that trimodality therapy using neoadjuvant chemo-radiation followed by surgery is the best approach.

At the time of writing, the CROSS regimen remains the standard of care[1]. The CROSS trial investigated over 360 patients with esophageal squamous cell and adenocarcinoma's and reported a nearly 2 year improvement in median survival (49 vs 26 months, $p = 0.011$), and an 11% improvement in 3 year OS for preoperative chemo-radiotherapy using low doses of weekly carboplatin (AUC2) and paclitaxel (50mg/m^2) [1] compared to surgery alone. A pathologic complete response rate of 29% was achieved with an improved rate of R0 resection (67% to 92%, $p < 0.002$). Increasingly however, oncologists are concerned about the minimal doses of chemotherapy used in this regimen and the lack of data showing a benefit of additional adjuvant chemotherapy in patients failing to achieve a complete response to trimodality therapy. This suggests that there is a significant opportunity to improve on neoadjuvant strategies in an effort to achieve a higher rate of pathological complete response.

In the trials performed to date in gastroesophageal tumors pathologic measures of improved overall survival (OS) after preoperative therapy and surgery include pathologic complete response, treatment effect equaling or exceeding 90%, down staging to node negative status or earlier T stage, and achievement of a negative margin R0 resection. Preoperative chemotherapy and chemo-radiotherapy have yielded mixed results in achieving these outcomes in esophageal and GEJ cancer. Additional support for the use of preoperative chemo-radiotherapy comes from the POET trial [2], comparing preoperative chemotherapy to sequential chemotherapy followed by chemo-radiotherapy. Chemo-radiotherapy achieved significantly higher rates of pathologic complete response (16% vs 2%, $p = 0.03$) and node negative status (64% vs 29%, $p =0.01$), and there were trends toward greater median survival (31 vs 21 months), 3 year OS (48% vs 28%, $p = 0.07$), and improved 3 year local tumor control (77% vs 59%, $p = 0.06$) all favoring chemo-radiotherapy.

Despite extensive study, there are still no validated predictive or prognostic molecular markers of outcome after preoperative therapy that might potentially guide treatment decisions. Radiological investigations acting as surrogate biomarkers have shown some promise but there remains a profound need for tissue based biomarkers in gastroesophageal cancer. The MUNICON trial demonstrated that PET scan non responding patients could be referred to earlier surgery, rather than continue ineffective systemic treatment, without a detriment in survival compared to continuing such therapy[3]. Early identification of treatment failure may spare patients from exposure both pre and postoperatively to inactive therapy. Non responding patients also have the potential to cross over to alternative therapies earlier on in treatment. Based on the MUNICON

and other PET scan trial results, the CALGB has opened trial 80803. Patients with esophageal and GEJ adenocarcinoma will receive induction chemotherapy with either mFOLFOX-6, or weekly carboplatin and paclitaxel. PET responders to induction therapy will then continue the same chemotherapy during subsequent combined chemo-radiotherapy, followed by surgery. PET non responders will cross over to the other regimen during radiotherapy, with the hope to optimize pathologic response in non-responders by changing chemotherapy during radiation.

Given the limited efficacy of cytotoxic chemotherapy in gastroesophageal cancer there is a clear need for innovative studies incorporating either immunological or molecularly targeted therapies. The only ongoing study in the US utilizing a biologic agent in the neoadjuvant setting is the RTOG 1010 study were trastuzumab is added to chemoradiotherapy (paclitaxel and carboplatin) in the 10-15% of patients with HER2 positive esophageal and GEJ cancer. This study has completed accrual and results are expected in the near future but even if positive this approach will only benefit a small population of patients.

1.1.2 Rationale for investigating checkpoint inhibitors in stage II/III gastroesophageal cancer.

The link between chronic inflammation, infection and malignancy has long been recognized in both esophageal and gastric cancers. For years, it has been postulated that targeting the immune system in upper GI cancers, may lead to improved outcomes in tumors that have proved inherently resistant to novel systemic treatments as a result of histological, molecular and etiological heterogeneity.

Gastroesophageal cancers develop in part as a result of prolonged chronic gastric reflux (GERD) -induced inflammation. In response to GERD, the occurrence of Barrett's metaplasia is accompanied by a change from an acute (TH1 type) immune response associated with IFNγ expression to a TH2-type chronic inflammation with production of IL4/IL13, which has been reported to result in an immunosuppressive, tumor-promoting microenvironment[4, 5]. This suggests that immunotherapeutics may have a significant role to play in earlier stage disease and studies investigating both neoadjuvant and adjuvant strategies in stage II/III esophageal cancer have been identified as key areas of research.

Although multiple factors are involved in predicting response to PD-1 inhibitors, it is thought high somatic mutational burden may be important and only melanoma, lung and bladder cancers display a more mutated profile than gastroesophageal cancers[6]. While not as prevalent as in gastric cancer, defective mismatch repair genes (MMR) have been identified in gastroesophageal junction and esophageal cancer and are predictive of response to PD-1 inhibition due to the fact that somatic mutations have the potential to encode "non-self" immunogenic neoantigens[7]. Whole-exome sequencing has demonstrated a mean of 1782 somatic mutations in mismatch repair–deficient tumors compared with approximately 73 in mismatch repair–proficient tumors[7]. An immunological assessment of the immune microenvironment in MMR deficient tumors has demonstrated strong expression of several immune checkpoint ligands most notably, PD-1/PD-L1, LAG-3, IDO and CTLA-4, which help confer resistance to immunological attack[8]. MMR deficiency has been identified in 17-21% of gastric cancers and preliminary data indicate a higher response rate in these patients of the order of approximately 50%[9].

Host immune evasion by tumor cells is an essential feature in the development and progression of human cancer. PD-1 is a co-inhibitory receptor expressed on the surface of activated and exhausted T-cells, B-cells and certain myeloid cells[10]. PD-L1 (programmed death – ligand-1), one of two ligands for PD-1, is highly expressed in certain human tumors and expression has been associated with a poor prognosis in both esophageal and gastric cancer[11].

1.1.3 PD-L1 expression in gastroesophageal cancer

We have recently published data on thirty-four resections of primary gastroesophageal junction and gastric carcinomas that were stained by immunohistochemistry using the 5H1 clone for PD-L1 and $CD8^+$ T cell densities both within tumors and at the tumor/stromal interface using whole slide digital imaging[11]. In this study, 12% of tumors demonstrated cell membranous PD-L1 expression whereas 44% showed expression within the immune stroma (**Figure 1**)[11]. Interestingly, we were also among the first to show that increasing $CD8^+$ densities both within tumors and immune stroma was associated with increasing percentage of tumor (p=0.027) and stromal (p=0.005) PD-L1 expression indicating an adaptive immune resistance mechanism may be occurring (**Figure 1 and Figure 2**). As demonstrated by others we also showed a worse overall survival and progression free survival in tumors that were PD-L1 positive [11].

Figure 1: Expression of PD-L1 by immune stroma associated with gastroesophageal adenocarcinomas

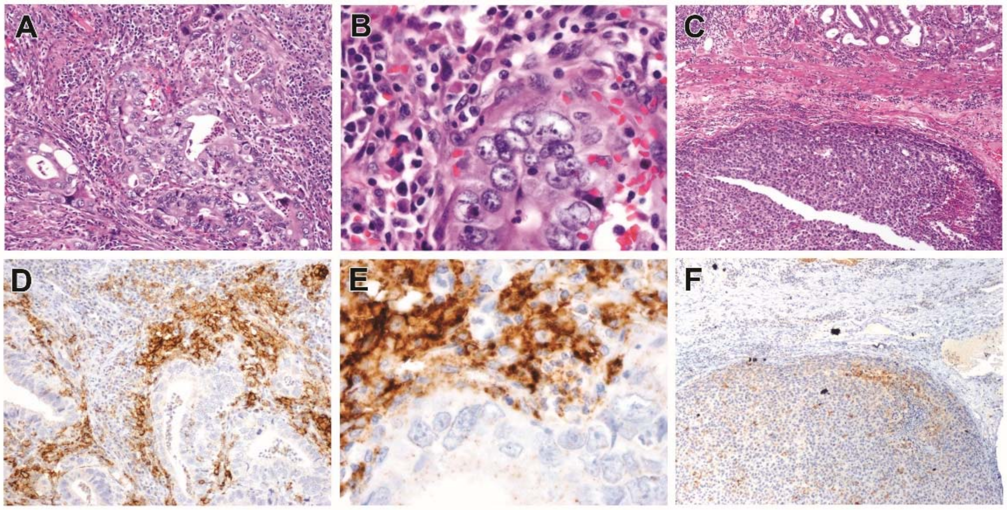

Figure 1 H & E (panels a, b, c) and PD-L1 staining (d, e, f) in two gastric adenocarcinomas with PD-$L1^{pos}$ (a, b, d, e) or PD-$L1^{neg}$ immune stroma (c, f). PD-L1 expression by immune stroma is seen in a peritumoral "interface" pattern. Shown at 100x (a, c, d, f) and 400x (b, e) original magnification. (Thompson, E.D. et al., *Patterns of PD-L1 expression and CD8 T cell infiltration in gastric adenocarcinomas and associated immune stroma.* Gut, 2016.)

Figure 2: PD-L1 expression by tumor cells and immune stroma increases with increasing CD8$^+$ density in each location.

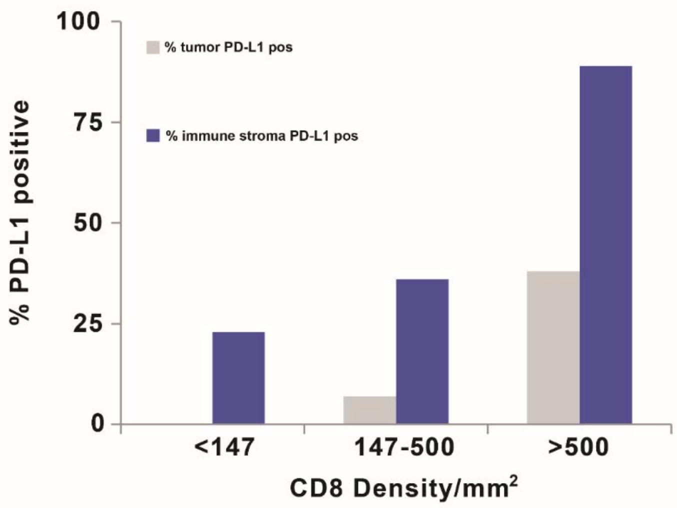

Figure 2: CD8 density within G/GEJ was determined by digital image analysis and densities were divided by quartiles into low (<147/mm^2), mid (147-500/mm^2) and high (>500/mm^2). Correlation between CD8 density and PD-L1 expression by location were determined using the exact version of the Cochran-Armitage trend test. PD-L1 expression by both tumor cells (p=0.0027) and immune stroma (p=0.005) increased with increasing CD8 density in each location. (Thompson, E.D. et al., *Patterns of PD-L1 expression and CD8 T cell infiltration in gastric adenocarcinomas and associated immune stroma.* Gut, 2016.)

Recent data from the CheckMate-032 study in metastatic esophageal and gastroesophageal cancers has demonstrated that PD-L1 is expressed in approximately 40% of advanced cancers and that nivolumab monotherapy has exciting activity in heavily pretreated patients.

A number of other investigators have determined that PD-L1 expression occurs in approximately 40% of gastric and GEJ cancers[12-14]. The recent KEYNOTE-012 phase 1b trial assessed pembrolizumab in patients with PD-L1 positive advanced gastric cancer as determined using the 22C3 antibody staining both tumor cells and mononuclear inflammatory cells. Out of the 162 patients screened, 65 (40%) were considered as PD-L1 positive tumors[9]

As outlined above we feel that investigating the efficacy of checkpoint inhibitors in the neo-adjuvant setting before surgery is an exciting prospect and is the basis for this investigator initiated proposal. Preoperative immunotherapy has the potential to improve pathological complete response rates in patients and allows a more comprehensive assessment of the immune response to checkpoint blockade in a tumor type with readily accessible tissue via the endoscope and at the time of surgical resection.

1.1.4 PD-L1 up-regulation after chemo-radiation in operable gastroesophageal cancer

Interestingly in unpublished work, Dr Anders from the department of pathology SKCCC evaluated 16 separate tumor samples from patients with stage II/III E/GEJ tumors both with baseline EGD and resected tumor samples and demonstrated that approximately 6% of resectable esophageal cancers have stromal PD-L1 expression at baseline endoscopic biopsy but this increases dramatically to 83% of tumors post chemo-radiation at the time of surgical resection (**Table 1 and Figure 3**). Interestingly we did not see high baseline PD-L1 expression indicating a weak endogenous antitumor immune response but following induction therapy there is a significant increase as previously predicted[15]. We have also determined the density and pattern of tumor infiltrating lymphocytes in GEJ/G adenocarcinoma patients. We compared the histologic features in resected gastroesophageal tumors in patients that received neoadjuvant chemo-radiation to those that did not. There was a consistent trend for neoadjuvant chemo-radiation to induce more tumor infiltrating lymphocytes (tumor lymphocytes), clustering of lymphocytes around tumor associated blood vessels (perivascular lymphocytes) and clusters of lymphocytes (tertiary lymphoid structures). The appearance of these microenvironment features after induction therapy suggest a tumor may respond favorable to immune based therapy and in particular to PD-1 based checkpoint blockade when combined with chemo-radiation.

Table 1: PD-L1 Expression in Esophageal and Gastro-esophageal Adenocarcinoma Patient Samples

SAMPLE TIMING	ADENOCARCINOMA CELL	TUMOR STROMAL CELLS
**PRE-CHEMO-RT N= 16**	0% (0/16)	6% (1/16)
**POST-CHEMO-RT N=12**	6% (1/16)	83% (10/12)

Figure 3: Stromal PDL1 expression is significantly enhanced post neo-adjuvant chemo-radiation in esophageal and gastro-esophageal cancers

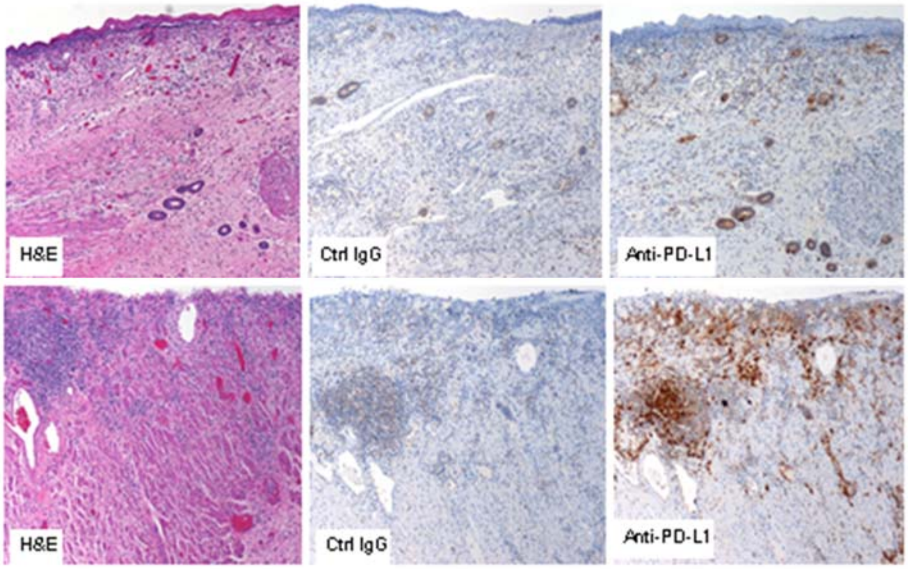

Figure 3: We determined PD-L1 expression in tissue samples obtained from 16 pre- and 12 post-neo adjuvant chemo-radiation therapy patients with distal esophageal / proximal gastric adenocarcinoma. PD-L1 was stained using our standard protocol [16] and scored for expression on malignant cells (tumor), tumor associated fibroblasts and lymphocytes (stroma). We found no expression of PD-L1 on the surface of malignant cells in either the pre- or post-treatment samples. However, there was significantly more expression of PD-L1 in the stromal compartment (83% 10/12) after chemo-radiation compared to virtually no expression before treatment (6% 1/16) the results are summarized in Table 1 with representative photomicrographs in Figure 3. (Robert Anders and Ronan Kelly, unpublished)

1.1.5 Previous studies of PD-1 or PD-1/CTLA-4 inhibition in gastroesophageal cancer

The efficacy of nivolumab either as single agent or combined with the CTLA-4 inhibitor ipilimumab has been assessed in gastroesophageal cancer. The CHECKMATE-032 study enrolled 160 patients with advanced/metastatic gastric or gastroesophageal junction cancer who had progressed on standard chemotherapy, most of whom received ≥ 2 prior regimens[17]. Patients were randomized to receive nivolumab 3 mg/kg every 2 weeks (N3) , nivolumab 1 mg/kg + ipilimumab 3 mg/kg (N1 + I3) or nivolumab 3 mg/kg + ipilimumab 1 mg/kg (N3+ I1) every 3 weeks for 4 cycles, followed by nivolumab 3 mg/kg every 2 weeks until disease progression or treatment was no longer tolerable. Treatment toxicities (any grade) were more common in the N1+I3 arm (84%) than the N3 (70%) or N3+I1 arms (75%). At the time of initial reporting, 96% (154) patients were evaluable for efficacy outcome reporting and the objective response rates in unselected patients was 14% (nivolumab alone), 26% (N1+I3) and 10% (N3+I1). Median overall survival was highest in the N1+I3 group (6.9 months, 95% CI 3.6 – not achieved), followed by N3 (5.0 months, 95% CI 3.4 – 12.4) and N3+I1 (4.8 months, 95% CI 3.0 – 9.1). In patients who were

PD-L1 positive defined as greater than 1% of cells staining positive, response rate for single agent nivolumab was 27% (4/15) and for the combination of the more active N1/I3 it was an impressive 44% (4/9). For PD-L1 negative patients <1% response rate for nivolumab was 12% (3/25) and for N1/I3 was 21% (6/29). These data are intriguing and demonstrate that although PD-L1 status should not be used to select for treatment, it may help us design future studies where it may be more appropriate for patients who are PD-L1$^-$ to be assigned to combination strategies. In addition it justifies an assessment of single agent and combination checkpoints inhibitors in earlier stage disease.

Table 2: Results from CheckMate 032 – clinical activity of nivolumab monotherapy and nivolumab plus ipilimumab combination therapy in subjects with previously treated metastatic GEJ/GC

	N3 mono (n=59)	N3 + I1 (n=52)	N1 + I3 (n=49)
**Confirmed ORR %**	13.6 (95% CI, 6-25)	10.2 (3.4-22.2)	26.1 (14.3-41.1)
**Dur of Resp (mos)**	7.1 (95% CI, 2.3-13.2)	NA (2.5 - NA)	5.6 (2.8 - NA)
**PFS rate 24 weeks %**	17.7 (95% CI, 9-28.7)	8.8 (2.8 – 19.1)	23.9 (12.4-37.5)
**Median PFS (mos)**	1.36 (95% CI, 1.25-1.51)	1.58 (1.38 - 2.6)	1.45 (1.25 – 3.94)
**1-year OS rate %**	36 (95% CI, 21 – 51)	NA	34 (19 – 50)
**Median OS (mos)**	5.03 (3.35 – 12.42)	4.83 (3.02 - 9.07)	6.87 (3.61 – NA)

Targeting PD-1 with nivolumab has also been shown to have promising efficacy in patients with advanced esophageal squamous cell cancer (SCC). In the ONO-4538 study, nivolumab (3 mg/kg every 2 weeks) was administered to 65 patients in a phase II, single arm, multi-center study in Japan and found that 17.2% % of patients achieved an objective response. The median overall survival was 12.1 months and serious adverse events occurred in 14% of patients but no treatment-related deaths occurred.

Given the potentially harmful effects of multiple chemotherapy treatment regimens on the immune system, it is quite possible that application of PD-1 pathway blockade earlier in E/GEJ cancer patients prior to receiving systemic doses of chemotherapy may significantly enhance its ability to induce immune-mediated cancer regression. In addition it is postulated that PD-L1 and other immune biomarkers may be induced by chemo-radiation therefore it is possible that combining checkpoint inhibitors with chemo-radiation in the non-metastatic setting may lead to enhanced efficacy over current standard of care treatments. Combination Nivolumab plus Relatlimab has been investigated in the ongoing FRACTION study which is open at Johns Hopkins and no toxicities to date have been seen with this combination in patients with metastatic gastroesophageal cancer. Preliminary efficacy appears promising.

1.1.6 Combining immunotherapy with radiation

There is growing evidence that radiation therapy targeted to a tumor can convert it into an in situ tumor vaccine by inducing release of antigens during cancer cell death in association with pro-inflammatory signals that trigger the innate immune system to activate tumor-specific T cells[18]. Radiation induces immunogenic cancer cell death in part by the upregulation of calreticulin to the surface of cancer cells together with the release of high-mobility group box-1 and adenosine triphosphate. This process promotes the uptake and cross-presentation of tumor antigens by dendritic cells to T cells in the draining lymph node[18]. Pro-immunogenic signaling is accompanied by interferon β production by the dendritic cells. To counteract this process a number of inhibitory immunosuppressive signals are induced including release of transforming growth factor β and colony stimulating factor 1 which result in enhanced infiltration of regulator T cells and myeloid-derived suppressor cells. This counterbalancing determines the extent of the development of an effective antitumor immune response.

In addition, radiation can have significant effects on the tumor microenvironment which can enhance infiltration of activated T cells and can overcome some of the barriers to tumor rejection. Radiotherapy can improve T-cell recruitment and infiltration into the tumor by reprogramming macrophages to secrete nitric oxide leading to vascular normalization. Enhanced tumor cell secretion of chemokines, such as CXCL10 and CXCL16, recruits CD8+ T cells (CTLs) to the tumor, and increased endothelial expression of vascular adhesion molecule-1 (VCAM-1) permits their extravasation[18]. Once inside the tumor, radiation-induced upregulation of major histocompatibility class I (MHC-I), ICAM-1, Fas, and natural-killer group 2, member D (NKG2D) ligands on the cancer cells improves their recognition and killing by cytotoxic T cells[18]. NKG2D ligand upregulation also improves NK cell-mediated killing of cancer cells that have lost MHC-I expression. Upregulation of programmed death ligand 1 (PD-L1) on cancer and/or immune cell infiltrating the tumor post radiation can be an obstacle to tumor rejection but it is hoped that by combining radiation with nivolumab this can be overcome.

A proof of principle study assessed the combination of GM-CSF with radiation in 41 patients with stable or progressing metastatic solid tumors[19]. Patients were treated with concurrent radiotherapy (35 Gy in ten fractions, over 2 weeks) to one metastatic site and granulocyte-macrophage colony-stimulating factor (125 μg/m(2) subcutaneously injected daily for 2 weeks, starting during the second week of radiotherapy). This course was repeated, targeting a second metastatic site. The primary endpoint was the proportion of patients with an abscopal response (defined as at least a 30% decrease in the longest diameter of the best responding abscopal lesion). Secondary endpoints were safety and survival. Abscopal responses occurred in 11 (26·8%, 95% CI 14·2-42·9) of 41 accrued patients (specifically in four patients with non-small-cell lung cancer, five with breast cancer, and two with thymic cancer). The most common grade 3-4 adverse events were fatigue (six patients) and haematological (ten patients). Additionally, a serious adverse event of grade 4 pulmonary embolism occurred in one patient. While additional research is required these data are promising and represent a first step in possibly establishing an in-situ anti-tumour vaccine.

Another recently published manuscript assessed the efficacy of CTLA-4 inhibition combined with radiation in malignant melanoma in patients and they reproduced the effect in a mouse model[20]. The investigators saw responses in both irradiated and un-irradiated tumors. Unbiased analyses of mice revealed that resistance was due to upregulation of PD-L1 on melanoma cells and associated

with T-cell exhaustion. Accordingly, the authors suggest that optimal response in melanoma and other cancer types requires radiation, anti-CTLA4 and anti-PD-L1/PD-1. Anti-CTLA4 predominantly inhibits T-regulatory cells (Treg cells), thereby increasing the CD8 T-cell to Treg (CD8/Treg) ratio. Radiation enhances the diversity of the T-cell receptor (TCR) repertoire of intratumoral T cells. Together, anti-CTLA4 promotes expansion of T cells, while radiation shapes the TCR repertoire of the expanded peripheral clones. Addition of PD-L1 blockade reverses T-cell exhaustion to mitigate depression in the CD8/Treg ratio and further encourages oligoclonal T-cell expansion. Similarly to results from mice, the patients treated on this clinical trial with melanoma showing high PD-L1 did not respond to radiation plus anti-CTLA4, demonstrated persistent T-cell exhaustion, and rapidly progressed. Thus, PD-L1 on melanoma cells allows tumors to escape anti-CTLA4-based therapy, and the combination of radiation, anti-CTLA4 and anti-PD-L1 promotes response and immunity through distinct mechanisms.

This proposed study will evaluate the safety and feasibility of preoperative administration of 2 cycles of induction nivolumab or nivolumab/relatlimab prior to concurrent chemo-radiation before surgical resection in patients with operable stage II/III distal esophageal cancers. Obtaining tissue at baseline EGD, after induction checkpoint inhibition and at the time of surgery will facilitate a comprehensive exploratory characterization of the tumor immune milieu and circulating immune cells and soluble factors in these patients. Data obtained in this safety study will provide valuable information for planning further prospective clinical trials of anti-PD-1 and other immunotherapies in E/GEJ cancers. Ultimately, it is highly desirable to discover prospective biomarkers of response and toxicity to allow patients with E/GEJ who are most likely to derive benefit to receive anti-PD-1 treatment or IO-IO combinations, and conversely to minimize the risk of toxicity and ineffective treatment for patients who are unlikely to benefit.

1.1.7 Analysis of Exploratory Features of Gut Microbiota that Correlate with Clinical Response

The population of microbes that inhabit humans (called the microbiota or microbiome) can influence both health and disease. The gastrointestinal tract or 'gut' microbiome is by far the most populous and complex. Studies in germ free mice have shown gut commensal bacteria shape the host immune system to maintain homeostasis and prevent the outgrowth of pathogens. Transfer of microbiota from diseased mice (e.g. metabolic syndrome, obesity) has been shown to transmit the disease to a healthy murine host suggesting the aggregate microbiota (or at least a consortium) encodes disease potential. In cancer, the gut microbiome has been linked to the development of gastric and colorectal malignancies, systemic tumor-promoting inflammation [21] and to anti-tumor immunity. The goal of the proposed study is to elucidate the biologic features of the gut microbiome and/or site-specific microbiomes of cancer patients receiving cancer therapies including checkpoint blockade or other immune-based treatments, radiation therapy and/or other cancer therapies (e.g., specific pathway inhibitors such as tyrosine kinase inhibitors). To accomplish this, we will aim to serially collect stool and/or other samples including tissue samples from cancer patients placed on cancer therapies. Initial samples, when feasible, will be collected at baseline prior to initiation of a therapy and then at clinically appropriate intervals during the therapeutic course. For those patients already on a cancer therapy, samples will be possibly collected in a cross-sectional design, at the point where declaration of therapy response or non-response is made by the treating physician. Potential

samples to be collected include, but are not limited to, stool and blood. Tissue sample acquisition for microbiome analyses will be determined by the treating physician, in consultation with the pathologist, and only excess tissue not required for clinical care will be analyzed for microbiome content. Samples will be analyzed for microbiome content (e.g., sequencing approaches such as 16S rRNA, metagenomic, RNASeq; proteomics; metabolomics; microbiology and/or other molecular methods) as well as, as appropriate, immune endpoints. Results will be correlated with clinical outcome and/or, for example, molecular markers indicating the status of the tumor. These studies will be complemented by experimental studies to test the hypothesis that the stools and/or other samples contain microbes that are beneficial or detrimental to the growth of implanted tumors in germ free mice. If this hypothesis is confirmed, individual microbes or consortia from the stools and/or other samples will be cultured and tested for an ability to alter the response to cancer therapies including immunotherapy and others (as above) in murine tumor models. A careful analysis of the samples from both responding and non-responding patients obtained before and after initiation of a therapeutic regimen, followed by confirmatory studies in germ free mice plus high-throughput sequencing, molecular and/or microbiologic culturing techniques [22], may provide a means to tailor the choice of therapy in cancer patients based on an analysis of their gut or other sample microbiota, or lead to the development of novel microbiome-based therapies.

1.2 Research Hypothesis

- Anti-PD-1 (nivolumab) or Anti-LAG3 (relatlimab) administration in the induction setting and nivolumab combined with chemoradiation will be safe and feasible in patients with resectable distal esophageal/gastroesophageal junction cancer.
- Nivolumab and the combination Nivolumab/ relatlimab, will change cellular and molecular characteristics of the tumor microenvironment that can be quantitatively measured.
- Failure to respond to anti-PD-1 or the combination of anti-PD-1/anti-LAG3 in esophageal cancer results from either pre-existing or compensatory (i.e. adaptive) up-regulation of additional immune "checkpoint" pathways in the tumor, draining lymph nodes, and/or peripheral blood that inhibit immune recognition and killing of tumor cells. Characterization of these pathways (i.e. ligands and receptors) in patients receiving preoperative checkpoint inhibition, and comparison with historical tumor samples where patients proceeded to surgical resection without preoperative anti-PD-1, will help us to understand the mechanisms of adaptation and immune resistance to directly guide future therapeutic development of anti-PD-1 as monotherapy and in combination with other immunomodulators in esophageal cancer.

1.3 Objectives

1.3.1 Primary Objective

To investigate the safety of induction nivolumab or nivolumab/ relatlimab administration prior to concurrent chemo-radiation in subject's with resectable stage II/III esophageal/gastro-esophageal junction cancer.

1.3.2 Secondary Objectives

- To investigate the feasibility of induction nivolumab or nivolumab/ relatlimab prior to concurrent chemo-radiation administration in subject's with stage II/III esophageal and gastro-esophageal junction cancer.

- To determine the pathological complete response rate in patients treated with induction checkpoint inhibition followed by chemo-radiation prior to surgical resection.

- To explore the association between nivolumab +/- relatlimab exposure and selected pharmacodynamics markers in the peripheral blood and in the tumor microenvironment, including measurement of PD-1 receptor occupancy on tumor infiltrating lymphocytes.

- To measure changes in expression of selected immune markers including changes in the quality and quantity of tumor infiltrating lymphocytes and the T effector to T-Reg ratio compared to baseline, in the blood, primary tumor tissue and draining lymph nodes

- To assess recurrence-free survival in patients receiving neoadjuvant checkpoint inhibitors.

- To assess overall survival in patients receiving neoadjuvant checkpoint inhibitors.

1.4 Product Development Background

1.4.1 Mechanism of Action

1.4.1.1 Nivolumab

Cancer immunotherapy rests on the premise that tumors can be recognized as foreign rather than as self and can be effectively attacked by an activated immune system. An effective immune response in this setting is thought to rely on immune surveillance of tumor antigens expressed on cancer cells that ultimately results in an adaptive immune response and cancer cell death. Meanwhile, tumor progression may depend upon acquisition of traits that allow cancer cells to evade immune-surveillance and escape effective innate and adaptive immune responses.

Current immunotherapy efforts attempt to break the apparent tolerance of the immune system to tumor cells and antigens by either introducing cancer antigens by therapeutic vaccination or by modulating regulatory checkpoints of the immune system. T-cell stimulation is a complex process involving the integration of numerous positive as well as negative co-stimulatory signals in addition to antigen recognition by the T-cell receptor. Collectively, these signals govern the balance between T-cell activation and tolerance.

PD-1 is a member of the CD28 family of T-cell co-stimulatory receptors that also includes

CD28, CTLA-4, ICOS, and BTLA. PD-1 signaling has been shown to inhibit CD-28-mediated Up-regulation of IL-2, IL-10, IL-13, interferon-γ (IFN-γ) and Bcl-xL. PD-1 expression also been noted to inhibit T-cell activation and expansion of previously activated cells. Evidence for a negative regulatory role of PD-1 comes from studies of PD-1 deficient mice, which develop a variety of autoimmune phenotypes. These results suggest that PD-1 blockade has the potential to activate anti-self T-cell responses, but these responses are variable and dependent upon various host genetic factors. Thus, PD-1 deficiency or inhibition is not accompanied by a universal loss of tolerance to self-antigens.

In vitro, nivolumab binds to PD-1 with high affinity (EC50: 0.39 - 2.62 nM), and inhibits the binding of PD-1 to its ligands PD-L1 and PD-L2 (IC50: ± 1 nM). Nivolumab binds specifically to PD-1 and not to related members of the CD28 family such as cytotoxic T-cell lymphocyte associated protein 4 (CTLA-4). Blockade of the PD-1 pathway by nivolumab results in a reproducible enhancement of both proliferation and IFN-γ release in the mixed lymphocyte reaction (MLR). Using a cytomegalovirus (CMV) re-stimulation assay with human peripheral blood mononuclear cells (PBMCs), the effect of nivolumab on antigen-specific recall response indicates that nivolumab augments IFN-γ secretion from CMV-specific memory T-cells in a dose-dependent manner versus isotype-matched control. In vivo blockade of PD-1 by a murine analog of nivolumab enhances the anti-tumor immune response and results in tumor rejection in several immunocompetent mouse tumor models (MC38, SA1/N, and PAN02).

1.4.1.2 Relatimab

Relatlimab - Anti-LAG3

Relatlimab (also referred to as BMS-986016, BMS-986016-01, and anti-lymphocyte activation gene 3 [LAG-3]) is a fully human LAG-3-specific antibody that was isolated following immunization of transgenic mice expressing human immunoglobulin (Ig) genes. It is expressed as an immunoglobulin G4 (IgG4) isotype antibody and includes a stabilizing hinge mutation (S228P). Relatlimab binds to the LAG-3 receptor with high affinity, and thus blocks LAG-3 interactions with its known ligand, major histocompatibility complex (MHC) Class II, which is the peptide antigen presentation molecule recognized by CD4+ T cells. Relatlimab binding inhibits the negative regulatory function of LAG-3 in vitro. By blocking the downregulatory pathway, relatlimab enhances the anti-tumor immune response and, thus, has the potential to inhibit the growth of multiple malignancies when administered as a single agent or in combination with other therapeutic immuno-oncology (IO) monoclonal antibodies (mAbs).

1.4.2 Summary of Clinical Pharmacology

1.4.2.1 Nivolumab

The PK of nivolumab were studied in subjects over a dose range of 0.1 to 10 mg/kg administered as a single dose or as multiple doses of nivolumab every 2 or 3 weeks. The geometric mean (%

CV%) clearance (CL) was 9.5 mL/h (49.7%), geometric mean volume of distribution at steady state (Vss) was 8.0 L (30.4%), and geometric mean elimination half-life (t1/2) was 26.7 days (101%). Steady-state concentrations of nivolumab were reached by 12 weeks when administered at 3 mg/kg Q2W, and systemic accumulation was approximately 3-fold. The exposure to nivolumab increased dose proportionally over the dose range of 0.1 to 10 mg/kg administered every 2 weeks. The clearance of nivolumab increased with increasing body weight. The PPK analysis suggested that the following factors had no clinically important effect on the CL of nivolumab: age (29 to 87 years), gender, race, baseline LDH, PD-L1.

Although ECOG status, baseline glomerular filtration rate (GFR), albumin and body weight had an effect on nivolumab CL, the effect was not clinically meaningful. When nivolumab is administered in combination with ipilimumab, the CL of nivolumab was increased by 24%, whereas there was no effect on the CL of ipilimumab. Additionally, PPK and exposure response analyses have been performed to support use of 240 mg Q2W dosing in addition to the 3 mg/kg Q2W regimen. Using the PPK model, exposure of nivolumab at 240 mg flat dose was identical to a dose of 3 mg/kg for subjects weighing 80 kg, which was the approximate median body weight in nivolumab clinical trials. Additional details are provided in the nivolumab investigator brochure (IB).

1.4.2.2 Relatlimab

An interim determination of relatlimab multiple dose PK was carried out using all available serum concentrations data from Studies CA224020 and CA224022. In general, the maximum observed concentration (Cmax) and area under the concentration versus time curve over the dosing interval (AUC[TAU]) values over the first dosing interval increased approximately proportional to the increment in the relatlimab dose. The PK of relatlimab and nivolumab was not altered when given in combination. Relatlimab population PK was best described by a 2-compartment model with parallel linear and non-linear clearance (CL). The linear portion represents the non-specific CL, and non-linear component represents target-mediated CL. The linear CL was 0.18 L/day, and the volume of distribution in the central compartment was 4.5 L. The maximum rate of non-linear elimination (Vmax) was 2.5 mg/day, and the concentration that achieved 50% of Vmax (Km) was 5.7 μg/mL. Currently available data suggest that relatlimab monotherapy exhibits a low level of immunogenicity, with 6 out of 42 subjects having at least 1 post-baseline positive ADA samples. There are limited data available in combination cohort to make inference on immunogenicity rate.

1.4.3 Safety Summary

Nivolumab has been studied in over 8,600 subjects and is widely approved in multiple indications. Extensive details on the safety profile of nivolumab, including results from other clinical studies, are available in the nivolumab IB, and will not be repeated herein. Overall, the safety profile of nivolumab alone or in combination with other therapeutic agents such as relatlimab is manageable and generally consistent across completed and ongoing clinical trials, with no MTD reached at any dose tested up to 10 mg/kg. Most AEs were low-grade (Grade 1 to 2) with relatively few related high-grade (Grade 3 to 4) AEs. There was no pattern in the incidence, severity, or causality of AEs with respect to nivolumab dose level. Results to date suggest that the safety profile of nivolumab/ relatlimab combination therapy is consistent with the mechanisms of action of nivolumab and

relatlimab. The nature of the AEs is similar to that observed with either agent used as monotherapy; however, both frequency and severity of most AEs are increased with the combination.

A pattern of immune-related AEs has been defined, for which management algorithms have been developed; these are provided in Appendix D. Most high-grade events were manageable with the use of corticosteroids or hormone replacement therapy (endocrinopathies) as instructed in these algorithms. For additional material, see the nivolumab IB.

Additional details on the safety profiles of nivolumab and relatlimab, including results from other clinical studies, are also available in the respective nivolumab and relatlimab IBs.

1.5 Overall Risk/Benefit Assessment

Subjects with stage II/III E/GEJ cancer represent a great unmet need with expected 5 year overall survival of only 20%. Preliminary results from the CheckMate 032 trial (the only IO-IO combination published to date albeit in the metastatic setting), in which nivolumab-plus-ipilimumab treatment was administered to heavily pretreated subjects with advanced/metastatic GC and GEJ cancers, demonstrated clinical activity in subjects whose tumors did or did not express PD-L1; better results were obtained in subjects with PD-L1 expressing (PD-L+) tumors. The toxicities seen with this combination are however well documented and new IO-IO combinations that are more tolerable are needed. The combination of nivolumab in combination with relatlimab is currently being investigated in the ongoing FRACTION study which is open at Johns Hopkins and no additional toxicities have been seen compared to nivolumab alone. This IO-IO combination is considered one of the most exciting in gastroesophageal cancer at the present time due to the high expression of LAG-3 in these tumors

This phase I safety study will enroll patients with earlier stage II/III disease and assess the safety of combining nivolumab with chemoradiation after induction nivolumab alone (arm A) or combination nivolumab/relatlimab (arm B). The safety profile of nivolumab when combined with ipilimumab in GEJ cancer, as measured by total and Grade 3/4 AEs, is comparable to that of a 1L platinum doublet but we do not have data on combining nivolumab with concurrent chemoradiation or combination nivolumab/relatlimab plus chemoradiation. In addition to this phase I safety study, the combination of single agent and combined checkpoint inhibitors is currently being assessed in other stages of gastroesophageal cancer (Table 3)

The safety profile of the nivolumab-plus-ipilimumab combination is characterized by immune-related toxicities, such as diarrhea, rash, pneumonitis, liver toxicity, and endocrinopathies. These events were mostly low grade and manageable with the use of corticosteroids. The toxicities with combination nivolumab plus relatlimab appear much less than nivolumab plus ipilimumab but studies are ongoing. We plan to only give 2 cycles of induction combination therapy prior to starting standard of care chemoradiation so we do not anticipate significant issues in the induction phase. Combining nivolumab +/- relatlimab with concurrent chemoradiation is untested.

Overall, the safety profile of nivolumab alone or in combination with other therapeutic agents such as relatlimab is manageable and generally consistent across completed and ongoing clinical trials, with no MTD reached at any dose tested up to 10 mg/kg. Most AEs were low-grade (Grade 1 to

2) with relatively few related high-grade (Grade 3 to 4) AEs. There was no pattern in the incidence, severity, or causality of AEs with respect to nivolumab dose level. Results to date suggest that the safety profile of nivolumab/ relatlimab combination therapy is consistent with the mechanisms of action of nivolumab and relatlimab. The nature of the AEs is similar to that observed with either agent used as monotherapy; however, both frequency and severity of most AEs are increased with the combination.

Table 3: Ongoing trials of interest or published data on nivolumab or nivolumab/ipilimumab (no published data for nivolumab plus relatlimab but ongoing FRACTION study is evaluating this combination and CheckMate 051 to open soon)

**Anti PD-1 or Anti -CTLA-4**	**Adjuvant or Neoadjuvant**	**1st line Metastatic**	**Refractory disease (Phase I/II trials)**
**Nivolumab**	**CheckMate 577 (PIII) adjuvant**<br><br>Nivo Vs placebo in resected stage II/III E/GEJ		**CheckMate 032 (PI/II)**<br><br>ORR 14% PD-L1 unselected<br><br>ORR 27% PD-L1 +ve (>1%)<br><br>**ONO-4538 (PII)**<br><br>ORR 17.2% (Asian pts, squamous cell esophageal)<br><br>ORR 23.8% (PD-L1 +ve)
**Nivolumab/Ipilimumab**		**CheckMate 649 (ongoing Phase III)**<br><br>Folfox or Xelox Vs Nivo/Ipi in previously untreated patients	**CheckMate 032 (PI/II)**<br><br>ORR 26% PD-L1 unselected<br><br>ORR 44% PD-L1 +ve (>1%)

**Nivolumab plus Relatlimab plus FOLFOX**		**CheckMate 051 (Phase III)**<br><br>**Nivolumab plus Relatlimab plus Folfox Vs Folfox in previously untreated metastatic patients**	

To ensure an ongoing favorable risk/benefit assessment for subjects enrolled in this study and to monitor for safety, patients will be seen weekly in the medical oncology clinic during the course of Chemorads/IO. During the induction phase they can be seen every 2 weeks in Arm A and in Arm B.

2. ETHICAL CONSIDERATIONS

2.1 Good Clinical Practice

This study will be conducted in accordance with Good Clinical Practice (GCP), as defined by the International Council on Harmonisation (ICH) and in accordance with the ethical principles underlying European Union Directive 2001/20/EC and the United States Code of Federal Regulations, Title 21, Part 50 (21CFR50) and applicable local requirements. The study will be conducted in compliance with the protocol. The protocol and any amendments and the subject informed consent form will receive Institutional Review Board/Independent Ethics Committee (IRB/IEC) approval/favorable opinion prior to initiation of the study. All potential serious breaches must be reported to the Sponsor or designee immediately. A serious breach is a breach of the conditions and principles of GCP in connection with the study or the protocol, which is likely to affect, to a significant degree, the safety or physical or mental integrity of the subjects of the study or the scientific value of the study. Personnel involved in conducting this study will be qualified by education, training, and experience to perform their respective tasks. This study will not use the services of study personnel where sanctions have been invoked or where there has been scientific misconduct or fraud (eg, loss of medical licensure, debarment).

2.2 Institutional Review Board/Independent Ethics Committee

Before study initiation, the investigator must have written and dated approval/favorable opinion from the IRB/IEC for the protocol, consent form, subject recruitment materials (e.g., advertisements), and any other written information to be provided to subjects. The investigator or BMS should also provide the IRB/IEC with a copy of the Investigator Brochure or product labeling information to be provided to subjects and any updates. The investigator, Sponsor or designee should provide the IRB/IEC with reports, updates and other information (eg, expedited safety reports, amendments, and administrative letters) according to regulatory requirements or institution procedures.

2.3 Informed Consent

Investigators must ensure that subjects are clearly and fully informed about the purpose, potential risks, and other critical issues regarding clinical studies in which they volunteer to participate.

In situations where consent cannot be given to subjects, their legally acceptable representatives are clearly and fully informed about the purpose, potential risks, and other critical issues regarding clinical studies in which the subject volunteers to participate. The principal investigator will provide an appropriate informed consent form which will include all elements required by ICH, GCP and applicable regulatory requirements. The informed consent form will adhere to the ethical principles that have their origin in the Declaration of Helsinki.

Investigators must:

- Provide a copy of the consent form and written information about the study in the language in which the subject is most proficient prior to clinical study participation. The language must be non-technical and easily understood.
- Allow time necessary for subject or subject's legally acceptable representative to inquire about the details of the study.
- Obtain an informed consent signed and personally dated by the subject or the subject's legally acceptable representative and by the person who conducted the informed consent discussion.
- Obtain the IRB/IEC’s written approval/favorable opinion of the written informed consent form and any other information to be provided to the subjects, prior to the beginning of the study, and after any revisions are completed for new information.
- If informed consent is initially given by a subject’s legally acceptable representative or legal guardian, and the subject subsequently becomes capable of making and communicating his or her informed consent during the study, consent must additionally be obtained from the subject.
- Revise the informed consent whenever important new information becomes available that is relevant to the subject's consent. The investigator, or a person designated by the investigator, should fully inform the subject or the subject's legally acceptable representative or legal guardian, of all pertinent aspects of the study and of any new information relevant to the subject's willingness to continue participation in the study. This communication should be documented.
- The confidentiality of records that could identify subjects must be protected, respecting the privacy and confidentiality rules applicable to regulatory requirements, the subjects' signed ICF and, in the US, the subjects’ signed HIPAA Authorization.
- The consent form must also include a statement that BMS and regulatory authorities have direct access to subject records.
- Subjects unable to give their written consent (e.g., stroke or subjects with or severe dementia) may only be enrolled in the study with the consent of a legally acceptable representative. The subject must also be informed about the nature of the study to the extent compatible with his or her understanding, and should this subject become capable, he or she should personally sign and date the consent form as soon as possible. The explicit wish of a subject who is unable to give his or her written consent, but who is capable of forming an opinion and assessing information to refuse participation in, or to be withdrawn from, the clinical study at any time should be considered by the investigator.
- The rights, safety, and well-being of the study subjects are the most important considerations and should prevail over interests of science and society.

3. INVESTIGATIONAL PLAN

3.1 Study Design and Treatment Plan

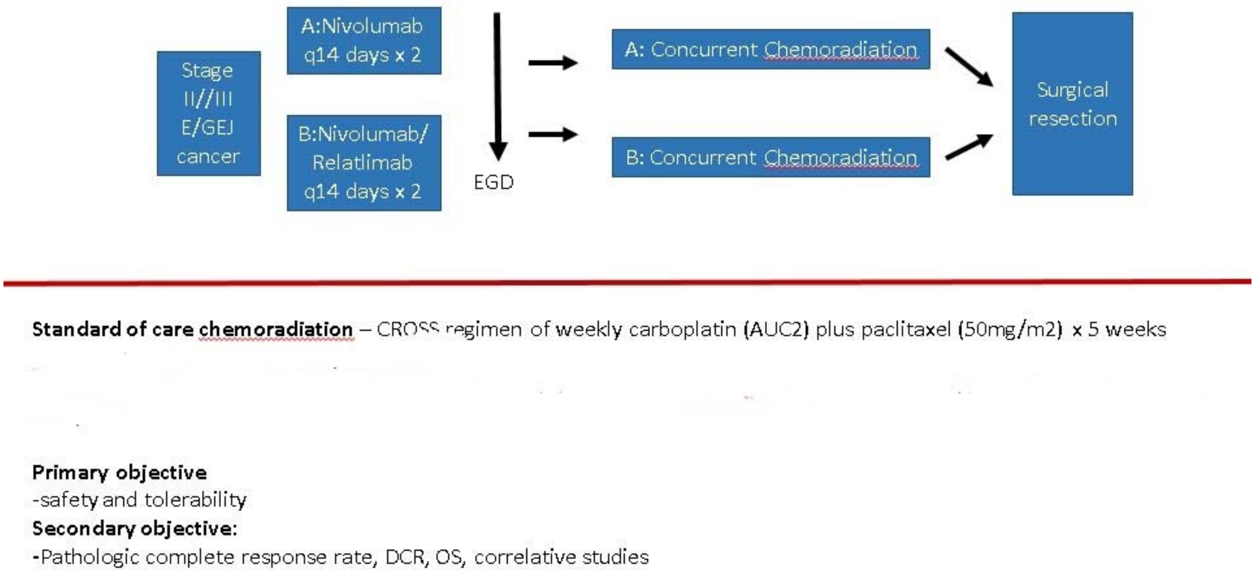

3.1.1 Recruitment

Patients will be recruited through the thoracic oncology clinics at Sidney Kimmel Comprehensive Cancer Center at Johns Hopkins (SKCCC), Charles A Sammons Cancer Center (Baylor), and at the Allegheny Health Network (AHN). Each enrolled subject will be assigned a participant sequential subject number at the time of consent e.g., 1-001, 1-002, etc.

3.1.2 Study Design and Toxicity Assessments

This is a phase I safety study that will be conducted at SKCCC, Baylor, and AHN.

3.1.3.1 Screening_- Eligible subjects will be consented to receive the investigational treatment (nivolumab or ipilimumab). The study sample size will consist of 32 patients. The staff at the treating center (SKCCC, Baylor, or AHN) will arrange drug supply and treatment. Nivolumab and Relatlimab will be supplied by Bristol-Myers Squibb Pharmaceuticals.

3.1.3.2 Treatment and collection of biological specimens-- 16 patients with resectable stage II or stage III distal esophageal or gastroesophageal junction tumors (AJCC staging version 7) will

receive induction nivolumab or nivolumab/ relatlimab before concurrent chemoradation prior to surgical resection. See section 6 for details on nivolumab or relatlimab administration.

Serial peripheral blood samples for exploratory analyses will be collected prior to each nivolumab dose, once within 3 days prior to surgery, and at 3-6 weeks after surgery. Preoperative core biopsy of the primary tumor will be obtained via EGD after induction therapy. Patients will have repeat PET/CT prior to surgery as per standard of care to re-assess tumor status and to assess for potential pulmonary inflammation following neoadjuvant treatment.

3.1.3.3 Toxicity assessments – Safety will be monitored continuously by the study investigators for the first sixteen patients through day 100 following the last dose of nivolumab or 30 days after surgery whichever is longer. Safety will be monitored on a continuous basis by the study investigators. A detailed statistical analysis plan for safety and feasibility is contained in section 12.0 of this protocol.

Dose Delays due to Toxicity – No dose delays due to toxicity will be permitted for patients enrolled on this study. Patients will continue with chemoradiation and immunotherapy can be re-introduced when immune related toxicities have resolved to ≤grade 1 and after consultation with the study PI.

General Management Algorithms for potential nivolumab and relatlimab related toxicities are contained in Appendices attached to this protocol and in the Investigators Brochure.

3.1.4 Diagnostic and surgical evaluation of participants

3.1.4.1 Diagnostic evaluation and pre-surgical workup

All patients enrolled on this protocol must be surgical candidates with clinical stage II/III esophageal cancer as per AJCC 7th edition (see appendix). Patients will have undergone EUS and radiographic evaluation indicating no evidence of distant disease and no evidence of un-resectable loco-regional tumor extension before surgical resection. Any further preoperative testing that is recommended by the surgeon or anesthesiologist will be performed as part of standard of care. Esophagogastrectomies for this study will be performed via a laparotomy and a right thoracotomy with en-bloc removal of perigastric, celiac, periesophageal and subcarinal lymph nodes. Esophagogastric reconstruction will be performed above the level of the azygo-caval junction using an EEA stapling device.

As per standard of care, patients should have a PET/CT scan performed prior to surgery to ensure there is no evidence of metastatic disease.

3.1.5 Tumor sample acquisition

Patients enrolled on this study will be required to have pretreatment primary tumor biopsy material available for diagnosis and exploratory immunological studies. This may consist of diagnostic EGD biopsies that have been previously performed, or biopsies conducted by the study team in the case of inadequate pre-existing material. Repeat EGD biopsies will be performed after 2 cycles of induction therapy, nivolumab (arm A) and nivolumab/relatlimab (arm B). Excisional biopsies, or at least 4 core needle biopsies (≤21 gauge diameter) of the primary tumor are required; fine needle aspirates will not be adequate. A minimum of ten 5-micron paraffin tissue sections is required. Biopsies that are formalin-fixed and paraffin embedded (FFPE) are required. Fresh frozen biopsy specimens may be analyzed in addition to, but not in place of, FFPE specimens.

Primary tumor, draining lymph nodes and normal esophageal specimens will be collected from patients who undergo surgical resection, after receiving neoadjuvant therapy. After removal of tissue necessary for clinical assessment, remaining tissue specimens for research purposes will be divided in surgical pathology into 1)fresh tissue that will be transported to the laboratory for viable cell isolation, 2)fixation and paraffin embedding (FFPE), and 3) flash frozen for DNA and RNA analysis. If additional tissue remains, frozen blocks in OCT (Optimal Cutting Temperature) compound will also be prepared.

Specific procedures for accessioning specimens are outlined in detail in the laboratory manual.

3.2 Postoperative treatment of participants

3.2.1 Adjuvant chemotherapy

Postoperative chemotherapy is controversial after trimodality therapy however this can administered at the discretion of the treating oncologist based on review of the surgical pathology and after a discussion with the principal investigator. Enrollment in the ongoing adjuvant nivolumab study CheckMate 577 is not allowed. Postoperative chemotherapy will start at a time based on the standard of care approach at the institution taking into account postoperative recovery time for the subject. Postoperative chemotherapy should not commence until nivolumab or relatlimab-related toxicity has resolved to <grade 1.

3.2.2 Postoperative radiation therapy

Postoperative radiation therapy will be not be administered as patients will have already received neoadjuvant radiation.

3.3 Evaluation of peri-operative safety

Trial subject's will be seen in clinic on a biweekly (induction phase) and then weekly (concurrent chemoradiation) basis from the start of therapy until after the last dose of

chemoradiation. Thereafter patients will be seen as per institutional standard of care prior to esophagectomy. Thereafter patients will be followed as per standard of care in the post-operative period. During the neoadjuvant phase toxicities will be reviewed at regular meetings (to take place once every 14 days) of study investigators and minutes of these meetings will be documented by the clinical research staff. In the event that a subject does not continue his or her peri-operative care at the institution, every attempt will be made to collect this information either by direct contact or through communication with the subjects outside physician(s).

4.0 Study Population

4.1 Inclusion criteria

4.1.1 Men and women aged ≥ 18 years old

4.1.2 Histologically proven (squamous cell or adenocarcinoma) esophageal or gastro- esophageal junction cancer (core biopsy required).

4.1.3 Stage II/III disease as per AJCC staging 7.0

4.1.4 Baseline imaging with standard of care FDG-PET scan and endoscopic ultrasound within 28 days prior to registration

4.1.5 ECOG performance status 0-1 (see Appendix B).

4.1.6 Adequate oral intake/nutritional status without the need for enteral or parenteral feeding during chemoradiation or preoperative period

4.1.7 Adequate organ function as follows:

- Leukocytes ≥ 2,000/mm^3
- Absolute neutrophil count (ANC) ≥ 1000/mm^3
- Platelet count ≥ 100,000/mm^3
- Hemoglobin ≥ 9 g/dL
- Creatinine ≤ 2.0 mg/dL
- Bilirubin (total) within normal institutional limits (except subjects with Gilbert Syndrome who must have total bilirubin < 3.0 mg/dL)
- AST(SGOT), ALT(SGPT), and alkaline phosphatase ≤ 2.5 times the upper limit of normal
- PT such that international normalized ratio (INR) is ≤ 1.5 (or an in-range INR, usually between 2 and 3, if a patient is on a stable dose of therapeutic warfarin and a PTT ≤ upper limit of normal

4.1.8 Adequate cardiac function as defined by: no evidence of PR prolongation or AV block on baseline electrocardiogram (ECG).

4.1.9 Radiation oncology consultation within 28 days to confirm that disease can be encompassed in the radiotherapy field and that normal tissue constraints can be met.

4.1.10 Subjects must have adequate lung function to permit surgical resection determined by pre-enrollment pulmonary function tests to include DLCO as follows:

- DLCO≥70% predicted **OR** DLCO<70% but ≥55% with a VO2 max ≥10L/min/kg (assessed by cardiopulmonary exercise testing) or 6 minute walk test ≥500 meters
- Subjects with a DLCO<55% are excluded from this study.
- Subjects must have a baseline O2 saturation by pulse oximetry that is ≥ 92% both at rest and while walking, off supplemental oxygen

4.1.11 Esophagogastrectomies will be performed via a laparotomy and a right thoracotomy with en-bloc removal of perigastric, celiac, periesophageal and subcarinal lymph nodes. Esophagogastric reconstruction will be performed above the level of the azygo-caval junction using an EEA stapling device.

4.1.12 Either a formalin fixed paraffin block or a minimum of ten 5-micron tissue section's (slides) of tumor biopsy sample must be available for biomarker evaluation from baseline and repeat EGD.

4.1.12 The effects of nivolumab or nivolumab/relatlimab, on the developing human fetus are unknown. For this reason women of child-bearing potential (WOCBP) and men must agree to use adequate contraception (hormonal or barrier method of birth control; abstinence) prior to study entry and for the duration of study participation and for 5 months after the last dose of nivolumab +/- relatlimab. Should a woman become pregnant or suspect she is pregnant while she or her partner is participating in this study, she should inform her treating physician immediately. Sexually active fertile men must use effective barrier birth control if their partners are WOCBP for 7 months after the last dose of nivolumab +/- relatlimab. WOCBP must have a negative serum or urine pregnancy test (minimum sensitivity 25 IU/L or equivalent units of HCG) within two weeks of registration.

4.1.13 Patient understands the study regimen, its requirements, risks and discomforts and is able and willing to sign the informed consent form. Voluntary signed and dated IRB/IEC approved written informed consent form in accordance with regulatory and institutional guidelines must be obtained before the performance of any protocol related procedures that are not part of normal patient care. Subjects must be competent to report AEs, understand the drug dosing schedule and use of medications to control AEs.

4.1.14 (Relatlimab arm only) LVEF (Left Ventricular Ejection Fraction) assessment with documented LVEF ≥ 50% by either TTE or MUGA (TTE preferred test) within 6 months from first study drug administration.

4.2 Exclusion Criteria

4.2.12 Patient has active, known or suspected autoimmune disease. Subjects with vitiligo, type I diabetes mellitus, residual hypothyroidism due to autoimmune thyroiditis only requiring hormone replacement, or conditions not expected to recur in the absence of an external trigger are permitted to enroll.

4.2.13 Esophageal tumors that are located in the mid esophagus or higher i.e. not involving distal esophagus or GE junction.
4.2.14 Tumors whose proximal end are higher that the level of the carina
4.2.15 Biopsy proven involvement of supraclavicular lymph nodes
4.2.16 Tumors extend 5cm or more into the stomach
4.2.17 Patient has a condition requiring systemic treatment with either corticosteroids (> 10 mg daily prednisone equivalent) or other immunosuppressive medications within 14 days of first dose. Inhaled or topical steroids and adrenal replacement steroid doses are permitted in the absence of active autoimmune disease.
4.2.18 Subjects with previous malignancies (except non-melanoma skin cancers, in situ bladder, gastric, breast, colon or cervical cancers/dysplasia) are excluded unless a complete remission was achieved at least 1 years prior to study entry and no additional therapy (other than adjuvant hormonal therapy for breast cancer) is required or anticipated to be required during the study period.
4.2.19 Subjects with known brain metastasis are excluded from this study. Patients with suspected brain metastasis must have brain imaging (either MRI brain or CT brain with contrast) prior to enrollment.
4.2.20 Subjects with a history of interstitial lung disease.
4.2.21 Active systemic infection requiring therapy, positive tests for Hepatitis B surface antigen or Hepatitis C ribonucleic acid (RNA).
4.2.22 Known positive history or positive test for Human Immunodeficiency Virus or Acquired ImmunoDeficiency Syndrome (AIDS).
4.2.23 History of allergy to study drug components.
4.2.24 Women who are pregnant or nursing.

4.2.25 WOCBP and Men with female partners (WOCBP) that are not willing to use contraception.

4.2.26 Prior therapy with an anti-PD-1, anti-PD-L1, anti-PDL-2, or anti-LAG-3 antibody (or any other antibody targeting T cell co-regulatory pathways).

4.2.27 Underlying medical conditions that, in the Investigator's opinion, will make the administration of study drug hazardous or obscure the interpretation of toxicity or adverse events.

4.2.28 Prisoners or subjects who are involuntarily incarcerated or compulsorily detained for treatment of either a psychiatric or physical (e.g. infectious disease) illness.

4.2.29 (Relatlimab arm only) Troponin T (TnT) or I (TnI) > 2 × institutional ULN. Subjects with TnT or TnI levels between > 1 to 2 × ULN will be permitted if repeat levels within

24 hours are ≤1 x ULN. If TnT or TnI levels are > 1 to 2 × ULN within 24 hours, the subject may undergo a cardiac evaluation and be considered for treatment, following a discussion with the BMS Medical Monitor or designee. When repeat levels within 24 hours are not available, a repeat test should be conducted as soon as possible. If TnT or TnI repeat levels beyond 24 hours are < 2 x ULN, the subject may undergo a cardiac evaluation and be considered for treatment, following a discussion with the BMS Medical Monitor or designee.

4.2.30 (Relatlimab arm only) Participants must not have a history of myocarditis

4.2.31 (Relatlimab arm only) Uncontrolled or significant cardiovascular disease including, but not limited to, any of the following:

- Myocardial infarction (MI) or stroke/transient ischemic attack (TIA) within the 6 months prior to consent
- Uncontrolled angina within the 3 months prior to consent
- Any history of clinically significant arrhythmias (such as ventricular tachycardia, poorly controlled atrial fibrillation, ventricular fibrillation, or torsades de pointes)
- QTc prolongation > 480 msec
- History of other clinically significant cardiovascular disease (i.e., cardiomyopathy, congestive heart failure with New York Heart Association [NYHA] functional classification III-IV, pericarditis, significant pericardial effusion, significant coronary stent occlusion, , poorly controlled venous thrombosis etc.)
- Cardiovascular disease-related requirement for daily supplemental oxygen
- History of two or more MIs OR two or more coronary revascularization procedures

Patients must meet all inclusion criteria and none of the exclusion criteria. Eligibility Waivers will not be granted.

4.3 Inclusion of Genders and Minorities

Individuals of all races and ethnic groups are eligible for this trial. There is no bias towards age, gender or race in the clinical trial outlined. This trial is open to the accrual of men and women who meet the inclusion/exclusion criteria outlined.

4.4 Concomitant Treatments

4.4.1 Prohibited or restricted treatments

The following medications are prohibited during the study (unless utilized to treat a drug related adverse event):

- Immunosuppressive agents
- Immunosuppressive doses of systemic corticosteroids

- Any concurrent anti-neoplastic therapy (ie, chemotherapy, hormonal therapy, immunotherapy, extensive, non-palliative radiation therapy other than on-study radiotherapy, or standard or investigational agents for treatment of GC/GEJ).

Subjects with a condition requiring systemic treatment with either corticosteroids (> 10 mg daily prednisone equivalent) or other immunosuppressive medications within 14 days of enrollment are excluded.

4.4.2 Permitted treatment

Subjects are permitted the use of topical, ocular, intra-articular, intranasal, and inhalational corticosteroids (with minimal systemic absorption). Adrenal replacement steroid doses > 10 mg daily prednisone are permitted. A brief (less than 3 weeks) course of corticosteroids for prophylaxis (eg, contrast dye allergy) or for treatment of non-autoimmune conditions (eg, delayed-type hypersensitivity reaction caused by a contact allergen) is permitted.

4.5 Discontinuation of Subjects following Treatment with Study Drug

Subjects MUST discontinue investigational product (and non-investigational product at the discretion of the investigator) for any of the following reasons:

- Subject's request to stop study treatment
- Any clinical adverse event (AE), laboratory abnormality or intercurrent illness which, in the opinion of the investigator, indicates that continued participation in the study is not in the best interest of the subject
- Loss of ability to freely provide consent through imprisonment or involuntarily incarceration for treatment of either a psychiatric or physical (e.g., infectious disease) illness
- Toxicity as specified in Section 6.4
- Disease progression assessed by RECIST 1.1 criteria, unless the subject meets criteria for treatment beyond progression.
- In the case of pregnancy, the investigator must immediately notify the Sponsor or designee of this event. In most cases, the study drug will be permanently discontinued in an appropriate manner (e.g., dose tapering if necessary for subject safety). Please contact the Sponsor or designee within 24 hours of awareness of the pregnancy. If the investigator determines a possible favorable benefit/risk ratio that warrants continuation of study drug, a discussion between the investigator and the Sponsor or designee must occur, if local regulations allow.

All subjects who discontinue study drug should comply with protocol specified follow-up procedures as outlined. The only exception to this requirement is when a subject withdraws consent for all study procedures including post-treatment study follow-up or loses the ability to consent freely (ie, is imprisoned or involuntarily incarcerated for the treatment of either a psychiatric or physical illness).

If study drug is discontinued prior to the subject's completion of the study, the reason for the discontinuation must be documented in the subject's medical records and entered on the appropriate case report form (CRF) page.

All subjects must be followed for safety for at least 100 days after the last dose of study therapy. Survival visits are every 3 months for the first year and thereafter follow standard NCCN guidelines.

4.6 Post-Study Drug Study Follow up

In this study, safety is the primary endpoint of the study. Post study follow-up is of critical importance and is essential to preserving subject safety and the integrity of the study. Subjects who discontinue study drug must continue to be followed for collection of outcome and/or survival follow-up data as required until death or the conclusion of the study.

4.7 Withdrawal of Consent

Subjects who request to discontinue study drug will remain in the study and must continue to be followed for protocol specified follow-up procedures. The only exception to this is when a subject specifically withdraws consent for any further contact with him/her or persons previously authorized by subject to provide this information. Subjects should notify the investigator of the decision to withdraw consent from future follow-up **in writing**, if possible. The withdrawal of consent should be explained in detail in the medical records by the investigator, as to whether the withdrawal is from further treatment with study drug only or also from study procedures and/or post treatment study follow-up, and entered on the appropriate CRF page. In the event that vital status (whether the subject is alive or dead) is being measured, publicly available information should be used to determine vital status only as appropriately directed in accordance with local law.

4.8 Lost to Follow-up

All reasonable efforts must be made to locate subjects to determine and report their ongoing status. This includes follow-up with persons authorized by the subject as noted above. Lost to follow-up is defined by the inability to reach the subject after a minimum of three documented phone calls, faxes, or emails as well as lack of response by subject to one registered mail letter.
All attempts should be documented in the subject's medical records. If it is determined that the subject has died, the site will use permissible local methods to obtain the date and cause of death. The investigator and representatives will consult publicly available sources, such as public health registries and databases, in order to obtain updated contact information. If after all attempts, the subject remains lost to follow-up, then the last known alive date as determined by the investigator should be reported and documented in the subject's medical records.

5. REGISTRATION PROCEDURES

5.1 General Guidelines

Eligible patients will be entered on study centrally at the Sidney Kimmel Comprehensive Cancer Center at the Johns Hopkins University by the Lead Study Coordinator. All sites should call/email

the coordinating center at crocc@jhmi.edu for registration. The Registration Form and Eligibility Worksheet will be supplied to each participating site.

Subjects who sign a consent form, but do not initiate protocol treatment for any reason (e.g., subjects who are screen failures), will be replaced and will not count towards our accrual goal. The Coordinating Center should be notified as soon as possible.

5.2 Registration Process

To register a patient, the following documents should be completed by the Research Nurse or Study Coordinator and emailed to crocc@jhmi.edu and the Study Coordinator to the Coordinating Center:

•Registration Form

•Signed patient consent form

•Eligibility Screening Checklist

•Copies of the following documents:

-Diagnostic pathology report(s)

-Baseline EGD/EUS

-LVEF by TTE or MUGA (Arm #B only)

-Standard of care CT or PET/CT scan report

Laboratory reports including:

-Complete blood count (CBC) with differential (including absolute lymphocyte count) and direct platelet count.

-Chemistry: Albumin, SGOT (AST), SGPT (ALT), Bilirubin (total) (Direct only if clinically indicated), Calcium, Creatinine, Glucose, Total protein, Urea nitrogen, (Uric Acid only if clinically indicated), Electrolytes (including sodium, potassium, chloride and bicarbonate), Troponin (Arm #B only).

-Baseline thyroid immune safety assay: Thyroid Stimulating Hormone (TSH). Abnormal endocrine results should be followed up per standard of care, and may require an endocrine consult and additional testing.

-Pulmonary function report and additional pulmonary testing if required according to eligibility criteria

-Other documents, if requested.

Confirmation of eligibility for patients at outside sites will require confirmation from the Protocol Chair (Vincent Lam) and a member of the Johns Hopkins Radiation Oncology team. This review and confirmation of eligibility may occur via email or a teleconference may be scheduled with the participating site and Johns Hopkins. Approval of subjects who meet the criteria for Variation Acceptable Upon Review by PI and Radiation Team will have confirmation of eligibility documented in a memo.

Study treatment cannot begin until the patient is registered.

The Research Nurse or Study Coordinator at the participating site will then e-mail (crocc@jhmi.edu and hschne12@jhmi.edu) the Coordinating Center to verify eligibility. To complete the registration process, the Coordinating Center will:

•Assign a patient study number

•Register the patient on the treatment portion of the study with the Sidney Kimmel Comprehensive Cancer Center's Clinical Research Office

•Fax or e-mail the patient study number to the participating site

6. Study Drugs - Pharmacology, safety and administration of nivolumab and relatlimab

6.1 Investigational Product

An investigational product, also known as investigational medicinal product in some regions, is defined a pharmaceutical form of an active substance or placebo being tested or used as a reference in a clinical study, including products already with a marketing authorization but used or assembled (formulated or packaged) differently than the authorized form, or used for an unauthorized indication, or when used to gain further information about the authorized form.

The investigational product should be stored in a secure area according to local regulations. It is the responsibility of the investigator to ensure that investigational product is only dispensed to study subjects. The investigational product must be dispensed only from official study sites by authorized personnel according to local regulations.

In this protocol, the investigational products are Nivolumab solution for injection and Relatlimab solution for injection.

6.2 Storage of Study Drug

The product storage manager should ensure that the study drug is stored in accordance with the environmental conditions (temperature, light, and humidity) as determined by BMS. If concerns regarding the quality or appearance of the study drug arise, the study drug should not be dispensed and BMS should be contacted immediately. Study drug not supplied by BMS will be stored in accordance with the package insert. Investigational product documentation (whether supplied by BMS or not) must be maintained that includes all processes required to ensure drug is accurately administered. This includes documentation of drug storage, administration and, as applicable, storage temperatures, reconstitution, and use of required processes (eg, required diluents, administration sets). Infusion-related supplies (eg, IV bags, in-line filters, 0.9% NaCL solution, or

pump) will not be supplied by the sponsor and should be purchased locally if permitted by local regulations. Please refer to the current versions of the IBs, SPC or package inserts, and/or pharmacy manual for complete storage, handling, dispensing, and infusion/dosing information for nivolumab, relatlimab, carboplatin, paclitaxel.

6.3 Nivolumab and Relatlimab Dose and Schedule

Subjects in Arm A will receive nivolumab 240mg administered IV over 30 minutes on day 1 of each treatment cycle every 2 weeks for 2 doses followed by initiation of concurrent chemo-radiation using weekly carboplatin and paclitaxel.

Subjects in Arm B will receive will receive nivolumab 240mg administered IV over 30 minutes followed by relatlimab 80mg administered IV over 60 minutes on Day 1 every 2 weeks for 2 doses followed by initiation of concurrent chemo-radiation using weekly carboplatin and paclitaxel.

For administration of single agent nivolumab or nivolumab/relatlimab– nivolumab +/- relatlimab may be administered up to 2 days before or 3 days after the scheduled date if necessary. Subjects may be dosed no less than 12 days between doses.

See Table 4 for details on the dosing schedule of nivolumab and relatlimab.

(**Refer to Relatlimab Investigational Brochure for additional information on drug preparation and administration**) Relatlimab injections are to be administered as an IV infusion through a 0.2/0.22-µm pore size, low-protein-binding polyethersulfone membrane in-line filter at the protocol-specified doses. It is not to be administered as an IV push or bolus injection. Relatlimab injection can be diluted with 0.9% sodium chloride injection (normal saline) or 5% dextrose injection (D5W) to protein concentrations no lower than 0.2 mg/mL. Detailed instructions for drug product dilution and administration are provided in the pharmacy manual for the clinical study.

Care must be taken to assure sterility of the prepared solution, as the product does not contain any anti-microbial preservative or bacteriostatic agent. No incompatibilities have been observed between relatlimab injection and ethylvinyl acetate (EVA), polyolefin (PO) or polyvinyl chloride (PVC) IV containers, di(2-ethylhexyl)phthalate (DEHP)-plasticized PVC IV sets, DEHP-free IV sets, or in-line filters with 0.2 µm or 1.2 µm polyethersulfone (PES), 0.2 µm nylon, or 0.2 µm polyvinylidene fluoride (PVDF) membranes.

Table 4: Schedule of drug administration

**Dose schedule of Nivolumab and Relatlimab**			
**Treatment group**	**Drug Name**	**Cycle 1**	**Cycle 2**
**Nivolumab**	**Nivolumab 240mg**	**Week 1**	**Week 3[a]**
**Nivolumab + Relatlimab**	**Nivolumab 240mg + Relatlimab 80mg**	**Week 1**	**Week 3a**

[a] Nivolumab +/- Relatlimab may be administered up to 2 days before or 3 days after the scheduled date if necessary. Subjects may be dosed no less than 12 days between doses..

Dosing calculations should be based on the body weight assessed at baseline. It is not necessary to re-calculate subsequent doses if the subject weight is within 10% of the weight used to calculate the previous dose. All doses should be rounded up to the nearest milligram per institutional standard.

Co-administration of Relatlimab Injection and Nivolumab

Relatlimab injections (10 mg/mL) can be coadministered with nivolumab injection (also referred to as BMS-936558 injection) as an IV infusion through a compatible low-protein-binding in-line filter at the protocol-specified doses. Relatlimab injection (10 mg/mL) may be mixed with nivolumab injection (10 mg/mL) as follows:

- Relatlimab injection (10 mg/mL) may be mixed with nivolumab injection (10 mg/mL) at a protein-mass ratio of 1:3 and the resultant drug product solution may be diluted with either NS or D5W to a total protein concentration no lower than 0.8 mg/mL (0.2 mg/mL of relatlimab and 0.6 mg/mL of nivolumab). total infusion volume of 160 mL. For patients weighing less than 40 kilograms (kg), the total volume of infusion must not exceed 4 mL per kg of patient weight. No incompatibilities have been observed between the combined drug product solutions and EVA, PO, or PVC IV containers, DEHP-plasticized PVC IV sets, DEHP free IV sets and in-line filters with 0.2 µm or 1.2 µm PES, 0.2 µm nylon, or 0.2 µm PVDF membranes
- Relatlimab injection (10 mg/mL) may be mixed with nivolumab injection (10 mg/mL) at a protein-mass ratio greater than 1:3 but less than or equal to a ratio of 1:1. The resultant drug product solutions may be diluted with NS to relatlimab concentrations ranging from 1.3 mg/mL to 4.0 mg/mL, with nivolumab concentration at 4.0 mg/mL. No incompatibilities have been observed between the combined drug product solutions and PO, or PVC IV containers, PVC IV sets, DEHP-free IV sets and in-line filters with 0.2 µm or 1.2 µm PES membranes. Detailed instructions for drug product dilution and administration are provided in the pharmacy manual for the clinical study.

Care must be taken to assure sterility of the prepared solution, as the products do not contain any anti-microbial preservative or bacteriostatic agent. No incompatibilities between the co-administered drug products and polyolefin or PVC bags, or non-DEHP or PVC infusion sets have been observed.

When study drugs (nivolumab and relatlimab) are to be administered on the same day, nivolumab is to be administered first. Nivolumab infusion must be promptly followed by a saline flush to clear the line of nivolumab before starting the relatlimab infusion. The second infusion will always be the relatlimab study drug and will start after the infusion line has been flushed, filters changed and the patient has been observed to ensure no infusion reaction has occurred. The time between infusions is expected to be approximately 30 minutes but may be more or less depending on the situation.

There will be no dose escalations or reductions of nivolumab or relatlimab allowed. In arm B subjects may be dosed no less than 18 days from the induction cycle of nivolumab plus relatlimab and during Q2W (nivolumab alone) cycles.

Pre-medications as per institutional standards are recommended for both nivolumab and nivolumab relatlimab. Subjects should be carefully monitored for infusion reactions during nivolumab or relatlimab administration. If an acute infusion reaction is noted, subjects should be managed according to Section 5.9.

Doses of nivolumab may be interrupted, delayed, or discontinued depending on how well the subject tolerates the treatment. For further information related to nivolumab and relatlimab preparation and dosing, please refer to the pharmacy manual.

Nivolumab 100 mg (10 mg/mL) will be packaged in an open-label fashion. Ten nivolumab 10 mL vials will be packaged within a carton. Relatlimab 200mg (5mg/ml) per 40 ml vial with 4 vials per pack.

6.4 Dose delay criteria for Nivolumab +/- Relatlimab Therapy

Nivolumab administration should be delayed for the following:

- Any Grade 2 non-skin, drug-related AE, with the following exception:
    - Grade 2 drug-related fatigue does not require a treatment delay.
- Grade 2 drug-related creatinine, AST, ALT or Total Bilirubin abnormalities
- Any Grade 3 skin, drug-related AE
- Any Grade 3 drug-related laboratory abnormality (excluding AST, ALT or Total Bilirubin), with the following exceptions for lymphopenia, and asymptomatic amylase or lipase abnormalities:
- Grade 3 lymphopenia does not require dose delay
- Any Grade 3 drug-related amylase or lipase abnormality that is not associated with symptoms or clinical manifestations of pancreatitis does not require dose delay.
- Any AE, laboratory abnormality, or intercurrent illness which, in the judgment of the investigator, warrants delaying the dose of study medication.
- Subjects receiving relatlimab in combination with nivolumab who have drug-related toxicities that meet the criteria for dose delay should have subsequent cycles of nivolumab delayed until retreatment criteria are met (exceptions apply to the retreatment criteria after dose delay following relatlimab and nivolumab for Grade 3 amylase and lipase

abnormalities that are not associated with symptoms or clinical manifestations of pancreatitis and that are attributed to relatlimab and/or nivolumab).

- Subjects who require delay of nivolumab should be re-evaluated weekly or more frequently if clinically indicated and resume nivolumab dosing when re-treatment criteria are met.
- Chemo-radiation will be commenced 2 weeks after 2nd cycle of induction Nivolumab (Arm A) or 2 weeks after two cycles of Nivolumab/Relatlimab (Arm B) but can be delayed if clinically indicated and commenced upon discussions between the PI and the Co-investigators.
- Any cardiovascular toxicities such as Heart failure or myocarditis should be discussed with the study PI and a decision can be made to delay or discontinue Relatlimab depending on the severity of the toxicity.

6.5 Dose Reductions for Nivolumab or RelatlimabTherapy

There will be no dose reductions for nivolumab or relatlimab.

6.6 Criteria for Resuming Treatment for Nivolumab or Relatlimab

Subjects may resume treatment with study drug when the drug-related AE(s) resolve to Grade 1 or baseline value, with the following exceptions:

- Subjects may resume treatment in the presence of Grade 2 fatigue.
- Subjects who have not experienced a Grade 3 drug-related skin AE may resume treatment in the presence of Grade 2 skin toxicity.
- For subjects with Grade 2 AST, ALT, or total bilirubin elevations, dosing may resume when laboratory values return to baseline and management with corticosteroids, if needed, is complete.
- Subjects with combined Grade 2 AST/ALT AND total bilirubin values meeting discontinuation parameters (Section 5.7) should have treatment permanently discontinued.
- Drug-related pulmonary toxicity, diarrhea, or colitis must have resolved to baseline before treatment is resumed. Subjects with persistent Grade 1 pneumonitis after completion of a steroid taper over at least 1 month may be eligible for retreatment if discussed with and approved by the principal investigator.
- Subjects with drug-related endocrinopathies adequately controlled with only physiologic hormone replacement may resume treatment. Adrenal insufficiency requires discontinuation regardless of control with hormone replacement.

If the criteria to resume treatment are met, the subject should restart treatment at the next scheduled time-point per protocol. However, if the treatment is delayed past the scheduled time-point per protocol, the scheduled study treatment administration will be delayed, but not skipped, until dosing resumes. In particular, this is to ensure that subjects will receive as many administrations of nivolumab +/- relatlimab treatments as legislated by the protocol.

If treatment is delayed > 8 weeks, the subject must be permanently discontinued from study therapy, and continue on standard of care treatment if possible.

6.7 Criteria for Nivolumab or Relatlimab Treatment Discontinuation

Treatment should be permanently discontinued for the following:

- Any Grade 2 drug-related uveitis, eye pain or blurred vision that does not respond to topical therapy and does not improve to Grade 1 severity within the re-treatment period OR requires systemic treatment
- Any Grade 3 non-skin, drug-related adverse event lasting > 7 days or recurs, with the Following exceptions: diarrhea, drug-related uveitis, pneumonitis, bronchospasm, hypersensitivity reactions, infusion reactions, and endocrinopathies:

    - Grade 3 drug-related diarrhea, uveitis, pneumonitis, bronchospasm, hypersensitivity reaction, or infusion reaction of any duration requires discontinuation
    - Grade 3 drug-related endocrinopathies adequately controlled with only physiologic hormone replacement do not require discontinuation. Adrenal insufficiency requires discontinuation regardless of control with hormone replacement
    - Grade 3 drug-related laboratory abnormalities do not require treatment discontinuation except:

        - Grade 3 drug-related thrombocytopenia > 7 days or associated with bleeding requires discontinuation
        - Any drug-related liver function test (LFT) abnormality that meets the following criteria require discontinuation:
            - Grade 3 drug-related AST, ALT or Total Bilirubin requires discontinuation*
            - Concurrent AST or ALT > 3 x ULN and total bilirubin > 2x ULN
            - In most cases of Grade 3 AST or ALT elevation, study drug(s) will be permanently discontinued. If the investigator determines a possible favorable benefit/risk ratio that warrants continuation of study drug(s), a discussion between the investigator and the principal investigator must occur.

- Any Grade 4 drug-related AE or laboratory abnormality (including but not limited to creatinine, AST, ALT, or total bilirubin), except for the following events which do not require discontinuation:
    - Grade 4 neutropenia ≤7 days
    - Grade 4 lymphopenia or leukopenia
    - Isolated Grade 4 amylase or lipase abnormalities that are not associated with symptoms or clinical manifestations of pancreatitis.
    - Isolated Grade 4 electrolyte imbalances/abnormalities that are not associated with clinical sequelae and are corrected with supplementation/appropriate management within 72 hours of their onset

  - Grade 4 drug-related endocrinopathy AEs, such as adrenal insufficiency, ACTH deficiency, hyper- or hypothyroidism, or glucose intolerance, which resolve or are adequately controlled with physiologic hormone replacement (corticosteroids, thyroid hormones) or glucose-controlling agents, respectively, may not require discontinuation after discussion with and approval from the principal investigator.

- Any event that leads to delay in dosing lasting > 8 weeks from the previous dose requires discontinuation, with the following exceptions:
  - Dosing delays to allow for prolonged steroid tapers to manage drug-related AEs are allowed.
  - Dosing delays lasting > 8 weeks from the previous dose that occur for non-drug-related reasons may be allowed if approved by the principal investigator.

- Any AE, laboratory abnormality, or intercurrent illness which, in the judgment of the Investigator, presents a substantial clinical risk to the subject with continued nivolumab Dosing.

- Any cardiovascular toxicities such as Heart failure or myocarditis should be discussed with the study PI and a decision can be made to delay or discontinue Relatlimab depending on the severity of the toxicity.

Prior to re-initiating treatment in a subject with a dosing delay lasting > 8 weeks from the previous dose, the principal investigator must be consulted. Patients will continue on standard of care chemoradiation if possible and will continue to have weekly study visits to assess safety and laboratory studies should also continue.

The assessment for discontinuation of relatlimab should be made separately from the assessment made for discontinuation of nivolumab if cardiovascular side-effects are the toxicity of concern. Although there is generally overlap among the discontinuation criteria, if discontinuation criteria are met for relatlimab but not for nivolumab (cardiovascular toxicities, treatment with nivolumab may continue in Arm B if approved by the study PI.

If a subject in the nivolumab/ relatlimab combination arm meets criteria for discontinuation and investigator is unable to determine whether the event is related to both or one study drug, the subject should discontinue both nivolumab and relatlimab and be continued on standard of care chemoradiation alone.

6.8 Management Algorithms for Immuno-oncology Agents

Immuno-oncology (I-O) agents are associated with AEs that can differ in severity and duration than AEs caused by other therapeutic classes. Nivolumab and relatlimab are considered I-O agents in this protocol. Early recognition and management of AEs associated with I-O agents may

mitigate severe toxicity. Management algorithms have been developed to assist investigators in assessing and managing the following groups of AEs:

- Gastrointestinal
- Renal
- Pulmonary
- Hepatic
- Endocrinopathy
- Skin
- Neurological

The above algorithms are found in the nivolumab IB and Appendix D of this protocol.

6.9 Treatment of Nivolumab- or Relatlimab-Related Infusion Reactions

Since nivolumab and relatlimab contain only human immunoglobulin protein sequences, they are unlikely to be immunogenic and induce infusion or hypersensitivity reactions. However, if such a reaction were to occur, it might manifest with fever, chills, rigors, headache, rash, pruritus, arthralgias, hypotension, hypertension, bronchospasm, or other allergic-like reactions.
All Grade 3 or 4 infusion reactions should be reported within 24 hours to the principal investigator and reported as an SAE if it meets the criteria. Infusion reactions should be graded according to NCI CTCAE version 4 guidelines. Treatment recommendations are provided below and may be modified based on local treatment standards and guidelines, as appropriate:

For Grade 1 symptoms: (mild reaction; infusion interruption not indicated; intervention not indicated):

- Remain at bedside and monitor subject until recovery from symptoms. The following prophylactic pre-medications are recommended for future infusions: diphenhydramine 50 mg (or equivalent) and/or acetaminophen/paracetamol 325 to 1000 mg at least 30 minutes before additional nivolumab or relatlimab administrations.

For Grade 2 symptoms: (moderate reaction required therapy or infusion interruption but responds promptly to symptomatic treatment (eg, antihistamines, non-steroidal anti-inflammatory drugs, narcotics, corticosteroids, bronchodilators, IV fluids); prophylactic medications indicated for 24 approx hours):

- Stop the nivolumab or relatlimab infusion, begin an IV infusion of normal saline, and treat the subject with diphenhydramine 50 mg IV (or equivalent) and/or acetaminophen/paracetamol 325 to 1000 mg; remain at bedside and monitor subject until resolution of symptoms. Corticosteroid and/or bronchodilator therapy may also be administered as appropriate. If the infusion is interrupted, then restart the infusion at 50% of the original infusion rate when symptoms resolve; if no further complications ensue after 30 minutes, the rate may be increased to 100% of the original infusion rate. Monitor subject

closely. If symptoms recur, then no further nivolumab or relatlimab will be administered at that visit.

- For future infusions, the following prophylactic premedications are recommended: diphenhydramine 50 mg (or equivalent) and/or acetaminophen/paracetamol 325 to 1000 mg should be administered at least 30 minutes before nivolumab or relatlimab infusions. If necessary, corticosteroids (up to 25 mg of hydrocortisone or equivalent) may be used.

For Grades 3 or 4 symptoms: (severe reaction, Grade 3: prolonged [ie, not rapidly responsive to symptomatic medication and/or brief interruption of infusion]; recurrence of symptoms following initial improvement; hospitalization indicated for other clinical sequelae (eg, renal impairment, pulmonary infiltrates). Grade 4: Life-threatening; pressor or ventilatory support indicated):

- Immediately discontinue infusion of nivolumab or relatlimab. Begin an IV infusion of normal saline and treat the subject as follows: Recommend bronchodilators, epinephrine 0.2 to 1 mg of a 1:1000 solution for subcutaneous administration or 0.1 to 0.25 mg of a 1:10,000 solution injected slowly for IV administration, and/or diphenhydramine 50 mg IV with methylprednisolone 100 mg IV (or equivalent), as needed. Subject should be monitored until the Investigator is comfortable that the symptoms will not recur. Nivolumab or relatlimab will be permanently discontinued. Investigators should follow their institutional guidelines for the treatment of anaphylaxis. Remain at bedside and monitor subject until recovery of the symptoms.

- In case of late-occurring hypersensitivity symptoms (eg, appearance of a localized or generalized pruritus within 1 week after treatment), symptomatic treatment may be given (eg, oral antihistamine or corticosteroids.

6.10 Chemotherapy Dosing - Carboplatin and Paclitaxel Dose and Schedule

All subjects will receive standard of care weekly carboplatin (AUC2) and paclitaxel (50mg/m2) for 5 weeks as per the CROSS regimen[1]. In the event of hematological toxicity or poor tolerance, chemotherapy can be held once discussed with the principal investigator. No dose modification of chemotherapy is allowed. If a week of chemotherapy is held it should not be made up at the end of radiation. Premedication and take home medications are as per standard of care.

6.11 Radiation Dosing and Schedule

- The prescription volumes is the planning target volume (PTV).
- The total dose will be 50.4 Gy in 28 fractions. A minimum of 95% of the PTV will receive the prescription dose.
- The GTVp is defined as the primary tumor in the esophagus. The GTVn is defined as any grossly involved regional lymph nodes. Target delineation is determined using EUS, PET and CT.

- The CTV is defined as the GTVp with a 3.5 cm expansion cranial/caudal and 1.0 cm radial expansion. CTV will include elective coverage of para-esophageal lymph nodes. For distal esophagus and gastroesophageal junction tumors, CTV will include celiac axis.
- The PTV will be added to the CTV with an expansion specified by the treating center. In most cases, 5-10mm expansion will be used.
- IMRT and VMAT technologies may be used, but attention to low dose exposure to the total lung is important . Specifically, normal tissue constraints for the V05 and V10 to the total lung will be strongly recommended, when feasible. It is recognized that some RT plans will not meet the constraints below and this is acceptable if necessary to achieve target coverage.

Table 5: Critical Structures and normal tissue toxicity:

Structure	Description	Metric	Per Protocol	Variation Acceptable	Variation Acceptable Upon Review by PI and Radiation Team**
Lung	Lungs- PTV	Max Dose (Gy, 0.93cc	≤110% Rx Dose	≤113%Rx Dose	>113% Rx Dose
		Mean Dose (Gy)	≤20 Gy	≤21 Gy	>21Gy
		V30	≤20%	≤35%	>25%
		V20	≤25%	≤30%	>30%
		V10	≤40%	≤50%	>50%
		V5	≤50%	≤55%	>55%
Heart 45	Heart & Pericardium	Max Dose (Gy 0.03cc)	≤50Gy	≤54GY	>52Gy
		Mean Dose (Gy)	≤30Gy	≤34Gy	>31Gy
		V40	≤50%	≤55%	>55%
Kidney	Combined Kidneys	Max Dose (Gy 0.03cc)	≤45Gy	≤50Gy	>50Gy
		V20	≤30%	≤40%	>40%

Spinal Cord	Spinal Cord	Max Dose (Gy, 0.03 cc)	≤45Gy	≤50Gy	>50Gy
Liver	Liver	Mean Dose (Gy)	≤21Gy	≤25Gy	>35Gy
		V3	≤30%	≤40%	≤40%

**Subjects who require radiation dosing as per Variation Acceptable Upon Review by the Principle Investigator and Radiation Team, must have their treatment plan and dosing reviewed prior to the initiation of radiation therapy. Documentation of this approval must be filed with the patient record.

(taken from RTOG 1010).

6.12 Treatment Compliance

Treatment compliance will be monitored by drug accountability as well as the subject's medical record and eCRF.

6.13 Destruction of Study Drug

The investigator will ensure that arrangements are made for the disposal of study drug according to applicable regulations, guidelines and institutional procedures, and appropriate records of the disposal have been documented.

7 Study Assessments and Procedures**

**Table 5: Screening procedural outline**		
**Procedure**	**Screening Visit[a]**	**Notes**
**Eligibility Assessments**		
Informed Consent	X	
Inclusion/Exclusion Criteria	X	
Medical History*	X	
Safety Assessments		
Physical Examination	X	
Smoking History	X	
Con-medication Review	X	
Vital Signs and O2 Saturation	X	O2 saturation by pulse oximetry should be ≥ 92% both at rest and while walking, off supplemental oxygen (oximetry should be done prior to dosing)
Assessment of Signs and Symptoms	X	
Laboratory Tests	X	CBC with differential, serum chemistry (BUN or serum urea level, serum creatinine, albumin, sodium, potassium, chloride, bicarbonate, and glucose levels), troponin (arm B only), AST,

		ALT, total bilirubin, Alk phosphatase, TSH, free T3 & T4, LDH, amylase, lipase, Hep B & C testing, PT/INR, PTT, and urinalysis.[d]
ECG	X	ECG
Echo/MUGA or TTE (Arm B only)	X	To determine baseline ejection fraction within 6 months of study drug adminstration
Pregnancy Test	X	This is only for WOCBP. Serum or Urine pregnancy test is required to be conducted within 2 weeks prior to registration
Physical measurements including ECOG status, Height and Weight	X	
Archived Tumor or Repeat Research Tumor Core Biopsy	X	This is mandatory for study entry. Sample should be received prior to first dose of nivolumab. Submit a copy of the original pathology report along with the sample.
*Must include radiologic or clinical stage at diagnosis.		

Procedure	Baseline	Day 1 (+/- 3 days)	Day 14 (+/- 3 days)	Concurrent chemoradiation	Preop Visit (As per Instiutional guidelines)	Post Surgery (Once during postoperative period day 21-42)	Follow up for recurrence-free and overall survival (Standard of Care Or every 3 Months)
**Clinical Assessments**							
Physical Exam	X	X	X	X	X	X	X
Vital Signs	X	X	X	X	X	X	X
ECOG PS	X	X	X	X	X	X	X
CBC with Diff	X	X	X	X	X	X	X
Chemistry/ TSH[b]	X	X[c]	X[c]	X[b, c]	X	X[b]	X
PT/INR, PTT					X		
Pregnancy Test (WOCBP Only)[a]	X[a]					X[a]	
**Treatment**							
Nivo (Arm A & B)		X	X				
Rela (Arm B Only)		X	X				
**Corre**	**Correlative Blood/ Tissue Samples**						
Serum, Plasma	X	X	X	X	X	X	
Tumor Biopsy/ Sample			X (repeat EGD post induction therapy and prior to chemoradiation)			X (i.e. Remember to send resection specimen)	
**Other assessments**							
PET Scan per SOC procedures	X				X (preoperative PET scan as per standard of care)		

Con Meds	X	X	X	X		X	X
Symptom/ Toxicity Assessment		X	X	X	X	X	X
Review of Medical Records, Telephone Contact for Recurrence-Free and Overall Survival							Up to 5 years

Stool Samples	X	X	X (Arm A Only)		X	X	X
Initial Sociodemographic Questionnaire	X						
Follow-up Sociodemographic questionnaire		X	X (Arm A Only)		X	X	X

WOCBP, women of child-bearing potential.
a = Repeat pregnancy test every 4 weeks (+/- 1 week) during treatment and 4-10 weeks post nivolumab for WOCBP.
b= free T3 and T4 should be collected every 6 weeks (every 3 infusions) for subjects receiving nivolumab and can be continued for 2 months in the post- operative period..
c= LDH, amylase, lipase prior to every immunotherapy dose (+/- 3 days)
d= On site/local complete blood count (CBC) w/differential, Chemistry panel including: LDH, AST, ALT, ALP, T-Bil, blood urea nitrogen (BUN) or serum urea level, creatinine, Na, K, Cl, total protein, glucose, albumin, amylase, lipase within 14 days prior to enrollment. Endocrine panel (TSH, Free T4, Free T3. Total T3/T4 are acceptable if free T3/T4 are not available), Hep B/C (HBV sAG, HCV antibody or HCV RNA), HIV (when required by local regulations), within 28 days prior to enrollment.

**In order to minimize the need for research-only in-person visits, telemedicine visits may be substituted for inperson clinical trial visits or portions of clinical trial visits where determined to be appropriate and where determined by the investigator not to increase the participants risks. Prior to initiating telemedicine for study visits the study team will explain to the participant, what a telemedicine visit entails and confirm that the study participant is in agreement and able to proceed with this method. Telemedicine acknowledgement will be obtained in accordance with the Guidance for Use of Telemedicine in Research. In the event telemedicine is not

deemed feasible, the study visit will proceed as an in-person visit. Telemedicine visits will be conducted using HIPAA compliant method approved by the Health System and within licensing restrictions.

8 Safety Assessments

8.1 Screening Safety Assessments

At screening, a medical history will be obtained to capture relevant underlying conditions. The screening examinations should include physical examination, weight, height, BMI, ECOG Performance Status, ECG, MUGA/TTE (Arm B only) and assessment of signs and symptoms. Screening assessments should be performed within 28 days prior to commencing on study unless otherwise specified. Vital signs (blood pressure [BP], heart rate and temperature) will be obtained at the screening visit and within 72 hours prior to first dose.

Screening local laboratory assessments should be done within 14 days prior to enrollment and are to include: CBC with differential, chemistry panel including LFTs (ALT, AST, total bilirubin, alkaline phosphatase), BUN or serum urea level, creatinine, Ca, Na, K, Cl, LDH, fasting glucose, albumin, amylase, Troponin (Arm B only) and lipase. The endocrine panel including TSH, free T3, and free T4. Hep B/C testing (HBV HBsAG, HCV antibody or HCV RNA) should be done within 28 days prior to enrollment.

Pregnancy tests for WOCBP must be performed at the screening visit and within 24 hours prior to the initial administration of study drug. Tumor tissue samples from baseline EGD must be available and if insufficient tissue the EGD should be repeated. This should be sent to the laboratory of Dr Drew Pardoll for PD-L1 status and biomarker analysis.

Serious AEs are to be collected as soon as the informed consent form is signed.

8.2 On-Treatment Safety Assessments

Subjects will be evaluated for safety if they have received any study drug. Adverse event (AEs and SAEs) assessments will be continuous during the treatment phase as well as during follow-up visits.

Adverse events and laboratory values will be graded according to the NCI-CTCAE version 4.

The start and stop time of the study therapy infusions and any interruptions or infusion rate reductions should be documented.

Physical examinations are to be performed as clinically indicated. If there are any new or worsening clinically significant changes since the last exam, report changes on the appropriate non-serious or serious adverse event page.

On-treatment local laboratory assessments are to be completed within 3 calendar days prior to dosing: CBC with differential, LFTs (ALT, AST, total bilirubin, alkaline phosphatase), BUN or serum urea level, creatinine, Ca, Na, K, Cl, LDH, glucose, amylase and lipase.

Thyroid function testing (TSH with reflexive fT3 and fT4) is to be done every 6 weeks (every 3 infusions) for subjects receiving nivolumab +/- relatlimab and can be continued for 2 months in the post- operative period.

On-treatment pregnancy tests should be performed as per the schedule in Table 6.

Additional measures, including non-study required laboratory tests, should be performed as clinically indicated or to comply with local regulations. Laboratory toxicities (eg, suspected drug induced liver enzyme evaluations) will be monitored during the follow-up phase via on site/local labs until all study drug related toxicities resolve, return to baseline, or are deemed irreversible.

If a subject shows pulmonary-related signs (hypoxia, fever) or symptoms (eg, dyspnea, cough, or fever) consistent with possible pulmonary AEs, the subject should be immediately evaluated to rule out pulmonary toxicity, according to the suspected pulmonary toxicity management algorithm in the BMS-936558 (nivolumab) IB.

Some of the assessments referred to in this section may not be captured as data in the eCRF. They are intended to be used as safety monitoring by the treating physician. Additional testing or assessments may be performed as clinically necessary or where required by institutional or local regulations.

8.3 Follow-up Safety Assessments

Adverse events will continue to be assessed and subsequent cancer therapy will be reviewed at each follow up clinic visit. Physical examination will also continue to be performed at follow-up visits. Laboratory tests including CBC and a comprehensive panel will be performed as per standard of care at each follow up clinic visit.

8.4 Imaging Assessment for the Study

Any incidental findings of potential clinical relevance that are not directly associated with the objectives of the protocol should be evaluated and handled by a study investigator as per standard medical/clinical judgment.

8.5 Efficacy Assessments

In this safety/feasibility study there will be no additional imaging performed outside of standard of care. Patients will require baseline PET/CT to stage the tumor and then prior to surgical resection patients will require additional PET/CT to ensure no evidence of metastatic spread. Both of these assessments are part of standard of care. In the adjuvant setting investigators will follow standard NCCN guidelines for imaging as part of standard of care follow up.

9 Exploratory immunologic studies

9.1 Immunologic correlates

All patients will undergo the same laboratory correlate studies on tumor biopsy, resection specimen and serum samples as subsequently enrolled patients.

9.2 Tumor Tissue Samples

9.2.1 Collection of pretreatment tumor and lymph node biopsies

Archived FFPE specimens from the original diagnostic EGD biopsy may be utilized. If they do not provide sufficient material for study, then new biopsies will be performed. At least 4 core needle biopsies of the primary tumor will be required at the time of diagnosis (prior to first dose of nivolumab). Where possible, and after a consent form has been signed, attempts will be made to coordinate diagnostic and study biopsies.

9.2.2 Pretreatment Biopsy Handling, Transportation, Storage, and Processing

Please see flow diagram in appendix C for overview of tissue collection and the laboratory manual for detailed procedures. The study staff will be notified when a biopsy is taking place. The following procedures will be followed:

If a core needle biopsy is being performed specifically for entry to the study, then at least four core-biopsy specimens will be obtained and suspended in 10% buffered formalin. After approximately 24 hours of suspension in formalin, the cores will be embedded in paraffin, sectioned into 5 µm-thick sections, and mounted onto slides. The slides will be stained for the appropriate studies listed below.

The study coordinator will keep a log with the study number, the patient's study number, the date and time, and a consecutive sample number; thus, the samples will be numbered serially and will not contain identifying information.

9.2.3 Operative specimens (tumor, normal esophagus, draining lymph nodes)

Tissue specimens obtained at the time of surgery will be dissociated enzymatically into single cell suspensions and will be viably cryopreserved according to a protocol provided in a companion laboratory manual. Additional specimens will fixed in formalin and embedded in paraffin blocks, for routine pathologic studies and immunohistochemistry. Tissue will also be flash-frozen at -80°C for subsequent RNA/DNA analysis. If there is additional tissue available, it will be embedded in OCT (Optimal Cutting Tissue) compound for analysis of frozen sections.

9.3 Blood Samples

Collection schedule

Blood samples will be drawn at the time points identified on the study calendar (**Table** 6).

Time points include:

- Prior to cycle 1 day 1 of nivolumab or nivolumab/ relatlimab administration): 100 ml whole blood or local protocol for collection accepted for peripheral blood mononuclear cell (PBMC) isolation, and 20 ml whole blood for serum isolation
- Prior to cycle 2 day 1 of nivolumab or nivolumab/relatlimab administration): 50 ml and 10 ml whole blood, for PBMCs and serum, respectively.
- Prior to cycle 3, 4 and 5 of with chemoradiation: 50 ml and 10 ml whole blood, for PBMCs and serum, respectively.
- Prior to surgery: 50 ml and 10 ml whole blood, as above.
- Once between Week 3-8 postoperatively (postoperative visit): 100 ml whole blood or local protocol for collection accepted for PBMC isolation, and 20 ml whole blood for serum preparation

9.4 Stool Samples

- Stool will be collected when possible by subjects using a kit that will be mailed or given to participants by the study team. Upon collection, subjects will be asked to store specimen in refrigerator or cool place until it can be brought with them to the clinic. Time points include:
- Once before initial research endoscopy
- Stools at the start of Nivolumab treatment (prior to Nivolumab +/- Relatlimab administration)
- Stools prior to second research endoscopy
- Stools prior to Chemoradiation
- Stools at end of Chemoradiation and prior to resection surgery
- Stool after resection
- Stools at routine follow-up visits every 3 months until evidence of recurrence
- Please collect questionnaires at baseline, every immunotherapy dose, and at followup if a sample is received.
- The sociodemographic questionnaire must be completed with each stool collection time-point. An initial questionnaire must be completed with the first sample collection and a follow-up questionnaire should be completed for each subsequent time-point. If a patient does not provide a sample as scheduled, this will be documented and no questionnaire completed.

9.5 Specimen handling, transportation, storage, processing (Appendix C)

Serum samples: Whole blood will be collected in serum separator tubes (Yellow Top Becton-Dickinson SST tube or equivalent), processed per manufacturer's instructions and stored at -70°C or below until transfer for analysis.

- PBMCs: Whole blood will be collected into purple tops tubes (Becton-Dickinson CPT tube or equivalent) and processed per manufacturer's instructions. Viable PBMCs will be stored in cryopreservation medium, at 5e6-1e7 per vial, in liquid nitrogen.

9.6 Methods of Analysis

9.6.1 Immunohistochemistry

Tumor and lymph node biopsies will be stained using commercially available and locally developed monoclonal antibodies. Analyses may include phosphorylated proteins of signaling pathways including but not limited to NF-kB, STAT3, RAS, MEK, and ERK; and phenotypes of infiltrating immune cell populations including but not limited to CD3, CD4, FoxP3, CD25, CD8, CD68, CD56, CD20, CD45RO and granzyme B. Peritumoral versus intratumoral infiltrates will be scored, since these staining patterns have been shown to correlate with clinical outcomes. Our pathologist Dr Thompson will assign an intratumoral and peritumoral immune cell infiltrate grade of (0) none, (1) rare lymphocytes (2) focal lymphohistocytic aggregates or (3) severe diffuse infiltration. Pathology will designate 3 representative fields to be evaluated by image analysis, which will allow for the data to be reported as a percentage of area with positive staining.

Immunohistochemical analysis of exploratory markers will focus on areas where the pathology co-investigators have established expertise, including but not limited to: the B7 family ligands PD-L1 (B7-H1), PD-L2 (B7-DC), B7-H3 and B7-H4, as well as inhibitory receptors on lymphocytes, including PD-1, 2B4, LAG-3, BTLA, and Tim-3; these cell surface molecules are candidates for therapeutic combinatorial antibody blockade. Expression of the ligands for Tim-3, BTLA and 2B4 (galectin 9, HVEM, and CD48, respectively) may also be evaluated as well as cytokine expression. These studies will provide a comprehensive view of cellular subsets and immune checkpoint molecule expression in tumors from untreated patients and how cellular subsets and key immune regulatory molecules are impacted intratumorally after treatment with anti-PD-1 and anti-CTLA-4. PD-L1 expression in FFPE specimens will be assessed with the mAb 5H1 and a manual staining technique, according to published methods and scoring criteria. PD-L1 testing with the BMS assay and JHU assay will be prioritized above other exploratory testing of the tissue specimens.

9.6.2 Amplified In Situ Hybridization (ISH)

ISH will be performed on FFPE sections using the RNAscope method from Advanced Cell Diagnostics. Genes to be probed include specific cytokines, such as IFN-g, IL-17, IL-10, IL-22, TGF-b, IL-4, TNF-a and certain chemokines. These studies will provide information on functional capacity of tumor infiltrating lymphocytes. Additionally, ISH will be performed for selected molecules, such as LAG-3, that are also being assessed by IHC. This will provide cross-validation for the two techniques.

9.6.3 Laser Capture Microdissection (LCM) and RNA Analysis

Complimentary to the ISH, LCM followed by RNA Analysis may be performed on FFPE sections. LCM will be performed by trained pathologists. RNA analysis will consist of qRT-PCR for selected immune genes, some of which are going to be analyzed in parallel by ISH, and also by whole genome microarray, using the DASL system. These analyses will provide a broader gene expression profile for broadly defined areas of the tumor (ie infiltrating tumor rests vs peri-tumoral vs surrounding stroma) and will complement ISH and IHC analyses.

9.6.4 Flow cytometric analysis of tumor and lymph nodes

Cryopreserved viable single cell suspensions will be thawed, and cells will be stained with specific monoclonal antibodies to assess coordinate expression of co-regulatory molecules by tumor infiltrating lymphocytes, draining lymph node cells and tumor cells. Multicolor flow cytometric analyses will be conducted. We will enumerate and characterize T cell subsets (e.g., CD4, CD8, CD25, HLA-DR, CD45RO, FoxP3, LAP, PD-1, PD-L1, PD-L2, LAG-3, ICOS, OX40, 41BB, central memory, effector memory), B cells (e.g., CD19, CD20, PD-1, PD-L2, ICOSL), dendritic cells and macrophages (e.g., CD68, CD83, CD1a, PD-L2, HLA-DR) and natural killer cells (CD56). Functional data and further demonstration of relevant T cell subsets will be gained from intracellular cytokine staining on T cells before and after non-specific CD3/28 activation (e.g., IFN-g, TNF-a, granzyme, IL-4, IL-10, and IL-17). The importance of these specific cytokines is that they mark distinct subsets of T cells with specific roles in pro- vs. anti-cancer immunity. In addition, blood samples will also be analyzed for the same markers, and for cytokines by multiplex assays.

9.6.5 PBMC analysis

Assessments of coordinate expression of co-regulatory molecules by PBMCs will be performed using multicolor flow cytometric analyses. T cell subsets (including CD4, CD8, and Treg with CD25 and Foxp3) will be analyzed as well as co-stimulatory and co-inhibitory molecule expression and markers for T cell activation state (e.g., CD25, HLA-DR, CD45RO, LAP, PD-1, PD-L1, LAG-3, ICOS, OX40, 41BB, central memory, effector memory). B cells (CD19, CD20, PD-1, PD-L1, PD-L2, ICOSL), dendritic cells and macrophages (CD68, CD83, CD1a, PD-L1, PD-L2, 4-1BB, 4-1BBL, ICOSL, HLA-DR) and natural killer cells (CD56) will be enumerated and characterized. Myeloid derived suppressor cells (MDSCs) will be enumerated by staining for CD14, CD11b, and HLA-DR expression. Further cytokines produced by T cells, will be analyzed by intracellular cytokine staining and multiplex assay. In certain cases, tetramer staining for populations of antigen-specific T cell populations may be performed.

9.6.6 Pharmacodynamic assessment of nivolumab

Approximate quantitation of infused nivolumab bound to PD-1 receptors on the surface of T cells in the peripheral blood and within the resected tumor and lymph node specimens will be performed in Dr. Pardolls laboratory, according to published procedures. This will provide information about tissue penetration of nivolumab, which has not been obtainable in prior studies.

9.6.7 Molecular pathway analysis

Genes and pathways that are significantly increased in post-therapy tumor tissues as compared to stage-matched untreated tumor tissues or pre-therapy tissues will be assessed by whole-genome analyses and confirmed by qRT-PCR as appropriate.

9.6.8 Serum analysis

Serum will be assessed for immunological factors which may include antibodies, cytokines and chemokines, as well as potentially for circulating tumor DNA. De-identified serum samples may also be provided to Dr. Glenn Dranoff's laboratory at the Dana Farber Cancer Institute, to study the potential role of antibodies to angiogenic cytokines in the therapeutic activity of nivolumab. This may include analysis of antibodies to angiopoietin-1/2, MIF, and VEGF-A with ELISAs, as previously reported. Patients that demonstrate high titer humoral reactions will then undergo detailed evaluation to isolate specific monoclonal antibodies.

9.7 Leftover Samples

Any leftover study blood and tissue samples will be stored in the Immunology Laboratories at Johns Hopkins for future research studies. These samples may be released for use in future studies after approval by the principal investigator and other regulatory bodies, as appropriate. Subjects will be asked to consent to the future use of samples in the consent document.

9.8 Additional Information

The laboratory investigators will be blinded to the subject identifiers and clinical data while generating the research data; additionally, the reported results will not disclose any unique patient identifiers.

Note: The correlative sample collection schedules outlined above are based on an ideal subject. The sample schedule should be followed as closely as is realistically possible;

however, the schedule may be modified due to problems such as scheduling delays or conflicts (e.g., clinic closure, poor weather conditions, vacations, etc.)

10 Adverse Events

10.1 General

This study will use the descriptions and grading scales found in the revised National Cancer Institute Common Terminology Criteria for Adverse Events (CTCAE) Version 4.0 for adverse event reporting that can be found at http://ctep.cancer.gov/reporting/ctc.html.

Information about all adverse events, whether volunteered by the subject, discovered by investigator questioning, or detected through physical examination, laboratory test or other means, will be collected, recorded, and followed as appropriate.

All adverse events experienced by subjects will be collected from the time of consent, throughout the study and until the final assessment (100 days after the last dose of nivolumab or 30 days after surgery whichever is longer) as outlined in the Study Calendar (table 6). Subjects continuing to experience toxicity after discontinuation of the study drug may be contacted for additional assessments until the toxicity has resolved or is deemed irreversible.

Any adverse event experienced during additional preoperative treatment or after the surgical procedure that the investigator feels is related to study treatment will be captured.

10.2 Definitions

10.2.1 Adverse Event (AE)

Defined as any new untoward medical occurrence or worsening of a pre-existing medical condition in a patient or clinical investigation subject administered an investigational (medicinal) product and that does not necessarily have a causal relationship with this treatment. An AE can therefore be any unfavorable and unintended sign (including an abnormal laboratory finding, for example), symptom, or disease temporally associated with the use of investigational product, whether or not considered related to the investigational product.

Adverse events can be spontaneously reported or elicited during open-ended questioning, examination, or evaluation of a subject. In order to prevent reporting bias, subjects should not be questioned regarding the specific occurrence of one or more AEs.
Medical conditions/diseases present before starting study treatment are only considered adverse events if they worsen after starting study treatment (any procedures specified in the protocol). Adverse events occurring before starting study treatment but after signing

the informed consent form will be recorded. Abnormal laboratory values or test results constitute adverse events only if they induce clinical signs or symptoms or require therapy.

10.2.2 Serious Adverse Event (SAE):

A serious AE (SAE) is any untoward medical occurrence that:
• results in death
• is life-threatening (defined as an event in which the subject was at risk of death at the time of the event; it does not refer to an event which hypothetically might have caused death if it were more severe)
• requires inpatient hospitalization or causes prolongation of existing hospitalization (see NOTE below)
• results in persistent or significant disability/incapacity
• is a congenital anomaly/birth defect
• is an important medical event (defined as a medical event(s) that may not be immediately life-threatening or result in death or hospitalization but, based upon appropriate medical and scientific judgment, may jeopardize the subject or may require intervention [i.e. medical, surgical] to prevent one of the other serious outcomes listed in the definition above.) Examples of such events include, but are not limited to, intensive treatment in an emergency room or at home for allergic bronchospasm; blood dyscrasias or convulsions that do not result in hospitalization.)

Potential drug induced liver injury (DILI) is also considered an important medical event. Wherever possible, timely confirmation of initial liver-related laboratory abnormalities should occur prior to the reporting of a potential DILI event. All occurrences of potential DILIs, meeting the defined criteria, must be reported as SAEs.

Potential drug induced liver injury is defined as:

1) AT (ALT or AST) elevation > 3 times upper limit of normal (ULN)

AND

2) Total bilirubin > 2 times ULN, without initial findings of cholestasis (elevated serum alkaline phosphatase),

AND

3) No other immediately apparent possible causes of AT elevation and hyperbilirubinemia, including, but not limited to, viral hepatitis, pre-existing chronic or acute liver disease, or the administration of other drug(s) known to be hepatotoxic.

Suspected transmission of an infectious agent (ie, any organism, virus or infectious particle, pathogenic or non-pathogenic) via the study drug is an SAE.

Although pregnancy, overdose, cancer, and potential drug induced liver injury (DILI) are not always serious by regulatory definition, these events must be handled as SAEs.

The following hospitalizations are not considered SAEs for the purposes of this study:

- a visit to the emergency room or other hospital department lasting < 24 hours, that does not result in admission (unless considered "important medical event" or event life threatening)
- elective surgery, planned prior to signing consent
- admissions as per protocol for a planned medical/surgical procedure
- routine health assessment requiring admission for baseline/trending of health status (ie, routine colonoscopy)
- medical/surgical admission for purpose other than remedying ill health state and was planned prior to entry into the study. Appropriate documentation is required in these cases.
- admission encountered for another life circumstance that carries no bearing on health status and requires no medical/surgical intervention (ie, lack of housing, economic inadequacy, care-giver respite, family circumstances, administrative).

10.2.3 Unexpected adverse event:

An adverse event, which varies in nature, intensity or frequency from information on the investigational drug/agent provided in the Investigator's Brochure, package insert or safety reports. Any adverse event that is not included in the informed consent is considered "unexpected". The Safety Information Section (5.6) of the Nivolumab IB and the Relatlimab IB should also be referenced.

10.2.4 Expected (known) adverse event:

An adverse event, which has been reported in the Investigator's Brochure. An adverse event is considered "expected", only if it is included in the informed consent document as a risk.

10.2.5 Relationship

The relationship of all adverse events and serious adverse events to study medication will be assessed by an investigator and assigned as follows:

Definitely: An adverse event which has a timely relationship to the administration of the investigational drug/agent, follows a known pattern of response, for which no alternative cause is present.

Probably: An adverse event, which has a timely relationship to the administration of the investigational drug/agent, follows a known pattern of response, but for which a potential alternative cause may be present.

Possibly: An adverse event, which has a timely relationship to the administration of the investigational drug/agent, follows no known pattern of response, but a potential alternative cause does not exist.
Unlikely: An adverse event which does not have a timely relationship to the administration of the investigational drug/agent, follows no known pattern of response, does not reappear or worsen after re-administration of the investigational drug/agent (if applicable), and for which there is evidence that it is related to a cause other than the investigational drug/agent.
Unrelated: An adverse event, for which there is evidence that it is definitely related to a cause other than the investigational drug/agent. In general, there is no timely relationship to the administration of the investigational drug/agent, or if there is a timely relationship, the event does not follow a known pattern of response, and there is an alternative cause.

10.3 Serious Adverse Event Collection and Reporting

Following the subject's written consent to participate in the study, all SAEs, whether related or not related to study drug, must be collected, including those thought to be associated with protocol-specified procedures.

General Management Algorithms for potential nivolumab/ relatlimab-related toxicities are contained in Appendix D of this protocol and in the Investigators Brochure.

All SAEs, must be collected after a study informed consent is signed that occur during the screening period and within 100 days of discontinuation of dosing for those subjects that receive study therapy (within 30 days of last visit for enrollment failure).The investigator should report any SAE occurring after these time periods that is believed to be related to study drug or protocol-specified procedure.

An SAE report should be completed for any event where doubt exists regarding its status of seriousness. If the investigator believes that an SAE is not related to study drug, but is potentially related to the conditions of the study (such as withdrawal of previous therapy, or a complication of a study procedure), the relationship should be specified in the narrative section of the SAE Report Form.

All AEs (both expected and unexpected) will be captured on the appropriate study-specific CRFs.

In addition, all SAEs, regardless of causality to study drug and/or administration device, will be reported promptly to the Coordinating Center (e-mail: crocc@jhmi.edu and hschne12@jhmi.edu), within 24 hours of recognition of the event. If this falls on a weekend or holiday, an email notification is acceptable but must be followed by an SAE reporting form on the next business day.

Coordinating Center

The Coordinating Center is the central location for the collection and maintenance of documentation of adverse events and is responsible for submitting adverse event reports to the Protocol Chair promptly. The Coordinating Center will maintain documentation of all adverse event reports for each participating site. Adverse event reports submitted to the Coordinating Center must be signed and dated by the participating site's Principal Investigator. The Coordinating Center will provide appropriate forms to be used by all participating sites for reporting adverse events. Information to be provided must include:

- Subject ID number, and initials
- Date of the event
- Description of the event
- Description of site's response to the event
- Assessment of the subject's condition
- Subject's status on the study (on study, off study, etc.)
- Attribution of event to study drug

Participating Sites

Participating sites are responsible for reporting adverse events to their IRB according to its specific requirements and to the Coordinating Center as follows:

Fatal Events whether anticipated or unanticipated, and whether or not related to the study must be reported to the Coordinating Center within 24 hours of the participating site Principal Investigator's learning of the event.

Serious and Unanticipated Adverse Events as defined above must be reported to the Coordinating Center within 24 hours of the participating site Principal Investigator's learning of the event.

Other Serious Adverse Events which may result in a change to the protocol, informed consent, or risk to subjects as specified in the protocol must be reported within three (3) working days of the participating site Principal Investigator's learning of the event.

Adverse Events which result in no change to protocol, informed consent, or risk to subjects must be reported to the Coordinating Center on a monthly basis.

Adverse event reports are to be emailed (use fax as a back-up) to the Coordinating Center at SKCCC. Follow-up reports are faxed, mailed, or sent electronically to the Coordinating Center as necessary.

The investigator must also report follow-up information about SAEs within the same time frames.

If a non-serious AE becomes serious, this and other relevant follow-up information must also be provided within the same time frames described above.

All SAEs must be collected whether or not they are considered causally related to the investigational product. Investigators and other site personnel are responsible for reporting all casually related SAEs to their IRB and the Protocol Chair.

All SAEs, whether related or unrelated to nivolumab or relatlimab and all pregnancies must be reported to the Coordinating Center within 24 hours.

The principal investigator will notify the appropriate regulatory agencies of any serious adverse event due to any cause during the course of this investigation. These include the Johns Hopkins Cancer Center Data and Safety Monitoring Committee, and the Johns Hopkins Medical Institutional Review Board (JHM-IRB) of The Johns Hopkins Medical Institutions. The required reporting time period is 3 days for fatal events, and 10 days for all other events.

For studies conducted under an Investigator IND, any event that is both serious and unexpected must be reported to the Food and Drug Administration (FDA) as soon as possible and **no later than 7 days** (for a death or life-threatening event) or **15 days** (for all other SAEs) **after the investigator's or institution's initial receipt of the information.** BMS will be provided with a simultaneous copy of all adverse events filed with the FDA.

SAEs should be reported on MedWatch Form 3500A or similar form. It MUST include the institutional **AND** BMS study ID [per study Agreement]

MedWatch SAE forms should be sent to the FDA at:
MEDWATCH
5600 Fishers Lane
Rockville, MD 20852-9787
Fax: 1-800-FDA-0178 (1-800-332-0178)
http://www.accessdata.fda.gov/scripts/medwatch/

The Coordinating center shall fax or e-mail the SAEs to BMS at:
Global Pharmacovigilance & Epidemiology
Bristol-Myers Squibb Company
Fax Number: 609-818-3804

SAE Email Address: Worldwide.Safety@BMS.com

The study period during which adverse events will be reported is from the initiation of study procedures to the end of the study treatment follow-up, defined as 100 days following the last administration of nivolumab treatment.

If only limited information is initially available, follow-up reports are required. (Note: Follow-up SAE reports should include the same investigator term(s) initially reported.) If an ongoing SAE changes in its intensity or relationship to study drug or if new information becomes available, a follow-up SAE report should be sent to BMS using the same procedure used for transmitting the initial SAE report.

In accordance with local regulations, BMS will notify investigators of all SAEs that are suspected (related to the investigational product) and unexpected (ie, not previously described in the Investigator Brochure). In the European Union (EU), an event meeting these criteria is termed a Suspected, Unexpected Serious Adverse Reaction (SUSAR). Investigator notification of these events will be in the form of an expedited safety report (ESR).

If only limited information is initially available, follow-up reports are required. (Note: Follow-up SAE reports should include the same investigator term(s) initially reported.) If an ongoing SAE changes in its intensity or relationship to study drug or if new information becomes available, a follow-up SAE report should be sent within 24 hours. All SAEs should be followed to resolution or stabilization.

The Sponsor will reconcile the clinical database AE cases (case level only) transmitted to BMS Global Pharmacovigilance (Worldwide.Safety@bms.com).

The Investigator will request from BMS GPV&E, aepbusinessprocess@bms.com the SAE reconciliation report and include the BMS protocol number every 3 months and prior to data base lock or final data summary.

GPV&E will send the investigator the report to verify and confirm all SAEs have been transmitted to BMS GPV&E.

The data elements listed on the GPV&E reconciliation report will be used for case identification purposes. If the Investigator determines a case was not transmitted to BMS GPV&E, the case should be sent immediately to BMS (Worldwide.Safety@bms.com).

In addition to the Sponsor Investigator's responsibility to report events to their local HA, suspected serious adverse reactions (whether expected or unexpected) shall be reported by BMS to the relevant competent health authorities in all concerned countries according to local regulations (either as expedited and/or in aggregate reports).

In accordance with local regulations, BMS will notify sponsor investigators of all reported SAEs that are suspected (related to the investigational product) and unexpected (ie, not previously described in the IB). An event meeting these criteria is termed a Suspected, Unexpected Serious Adverse Reaction (SUSAR). Sponsor investigator notification of these events will be in the form of either a SUSAR Report or a Semi-Annual SUSAR Report.
Other important findings which may be reported by BMS as an Expedited Safety Report (ESR) include: increased frequency of a clinically significant expected SAE, an SAE considered associated with study procedures that could modify the conduct of the study, lack of efficacy that poses significant hazard to study subjects, clinically significant safety finding from a nonclinical (eg, animal) study, important safety recommendations from a study data monitoring committee, or sponsor or BMS decision to end or temporarily halt a clinical study for safety reasons.

Upon receiving an ESR from BMS, the investigator must review and retain the ESR with the IB. Where required by local regulations or when there is a central IRB/IEC for the study, the sponsor will submit the ESR to the appropriate IRB/IEC. The investigator and IRB/IEC will determine if the informed consent requires revision. The investigator should also comply with the IRB/IEC procedures for reporting any other safety information.

10.4 Non-serious Adverse Events

A non-serious adverse event is an AE not classified as serious.

10.4.1 Non-serious Adverse Event Collection and Reporting

The collection of non-serious AE information should begin at initiation of study drug. Non-serious AE information should also be collected from the start of a lead-in period or other observational period intended to establish a baseline status for the subjects. All non-serious adverse events (not only those deemed to be treatment-related) should be collected continuously during the treatment period and for a minimum of 100days following the last dose of study treatment.

Non-serious AEs should be followed to resolution or stabilization, or reported as SAEs if they become serious. Follow-up is also required for non-serious AEs that cause interruption or discontinuation of study drug, or those that are present at the end of study treatment as appropriate.

Adverse Events that are routinely collected according to GCP shall be submitted to BMS every three (3) months by the last working day of the third month.

The Adverse Event information required to be sent to BMS is collected and sent to BMS via the mailbox:
MG-RD-GPVE-PHARMACOVIGILANCE@bms.com

When the file is submitted to BMS, it must be noted whether the file contains
1) all Non Serious Adverse Events (only adverse events not previously submitted to BMS within the 3 months).

All identified non-serious AEs must be recorded and described on the non-serious AE page of the CRF (paper or electronic).

Completion of supplemental CRFs may be requested for AEs and/or laboratory abnormalities that are reported/identified during the course of the study.

Serious AE CRF page or SAE Report Form (paper or electronic) as appropriate:
• Any laboratory test result that is clinically significant or meets the definition of an SAE
• Any laboratory abnormality that required the subject to have study drug discontinued
• Any laboratory abnormality that required the subject to receive specific corrective therapy.

It is expected that wherever possible, the clinical, rather than the laboratory term would be used by the reporting investigator (ie, anemia versus low hemoglobin value).

10.4.2 Pregnancy

If, following initiation of the investigational product, it is subsequently discovered that a study subject is pregnant or may have been pregnant at the time of investigational product exposure, including during at least 6 half-lives after product administration, the investigational product will be permanently discontinued in an appropriate manner. Protocol-required procedures for study discontinuation and follow-up must be performed on the subject unless contraindicated by pregnancy (ie, x-ray studies). Other appropriate pregnancy follow-up procedures should be considered if indicated.

The investigator must immediately notify the Johns Hopkins IRB of this event and complete and forward a Pregnancy Surveillance Form to BMS PVG within 24 hours and in accordance with SAE reporting procedures described in **Section 9.3**

Follow-up information regarding the course of the pregnancy, including perinatal and neonatal outcome and, where applicable, offspring information must be reported on the Pregnancy Surveillance Form.

Any pregnancy that occurs in a female partner of a male study participant should be reported to the sponsor. Information on this pregnancy will be collected on the Pregnancy Surveillance Form.

10.4.3 Overdose

An overdose is defined as the accidental or intentional administration of any dose of a product that is considered both excessive and medically important. All occurrences of overdose must be reported as SAEs (see Section 11.3 for reporting details).

10.4.4 Other Safety Considerations

Any significant worsening noted during interim or final physical examinations, electrocardiograms, x-rays, and any other potential safety assessments, whether or not these procedures are required by the protocol, should also be recorded as a non-serious or serious AE, as appropriate, and reported accordingly.

11 Data and Safety Monitoring

11.1 Data Management

Eligible patients will be entered on study centrally at the Sidney Kimmel Comprehensive Cancer Center at the Johns Hopkins University by the Lead Study Coordinator. All sites should call/email the coordinating center at crocc@jhmi.edu. The Registration Form, and Eligibility Worksheet will be supplied to each participating site.

If a patient does not receive protocol therapy following registration, the patient's registration on the study may be canceled. The Coordinating Center should be notified of cancellations as soon as possible.

At the time of registration:

- Registration Form
- Informed Consent Form (signed by the subject)
- Eligibility Checklist
- Source documents related to eligibility and enrollment

Within 2 weeks after registration:

- Baseline study case report forms
- Pertinent source documents

Within 2 weeks after final dose of study medication:

- On study case report forms
- Pertinent source documents

The Study Coordinator at the participating site will then e-mail (crocc@jhmi.edu and the Coordinating Center lead study coordinator) to verify eligibility. To complete the registration process, the Coordinating Center will:

- Assign a patient study number
- Register the patient on the treatment portion of the study with the Sidney
- Kimmel Comprehensive Cancer Center's Clinical Research Office
- Fax or e-mail the patient study number to the participating site
- Call or e-mail the research nurse or data manager at the participating site and
- Verbally confirm registration the last eligible start date for treatment.

The investigator will permit study-related monitoring, audits, and inspections by the IRB, government regulatory bodies, and University compliance and quality assurance groups of all study related documents. The investigator will ensure the capability for inspections of applicable study-related facilities (e.g. pharmacy, diagnostic laboratory, etc.).

The PI is responsible for internally monitoring the study and establishing additional external data & safety monitoring oversight, as required. The PI will also monitor the progress of the trial, review safety reports, and confirm that the safety outcomes and response assessments favor continuation of the study.

The SKCCC Compliance Monitoring Program will provide external monitoring for JHU-affiliated sites in accordance with SKCCC DSMP (Version 6.0, 02/21/2019). The SMC Subcommittee will determine the level of patient safety risk and level/frequency of monitoring. The protocol will be monitored internally (Johns Hopkins East Baltimore and Bayview Medical Center Campuses) at SKCCC by the Principal Investigator and externally by the SKCCC CRO in accordance with SKCCC guidelines. The Johns Hopkins SKCCC CRO Coordinating Center will be responsible for data monitor at AHN and Charles A Sammons Cancer Center.

11.2 Multicenter Guidelines

Protocol Chair

- The Protocol Chair is responsible for performing the following tasks:
- Coordinating, developing, submitting, and obtaining approval for the protocol
- as well as its subsequent amendments

- Assuring that all participating institutions are using the correct version of the protocol.
- Taking responsibility for the overall conduct of the study at all participating institutions and for monitoring the progress of the study.
- Reviewing and ensuring reporting of Serious Adverse Events (SAE)
- Reviewing data from all sites

Coordinating Center

The Coordinating Center is responsible for performing the following tasks:

- Ensuring that IRB approval has been obtained at each participating site prior to the first patient registration at that site, and maintaining copies of IRB approvals from each site.
- Managing central patient registration.
- Collecting and compiling data from each site.
- Establishing procedures for documentation, reporting, and submitting of AE's and SAE's to the Protocol Chair, and all applicable parties.
- Facilitating audits by securing selected source documents and research records from participating sites for audit, or by auditing at participating sites.

Participating Sites

Participating sites are responsible for performing the following tasks:

- Following the protocol as written, and the guidelines of Good Clinical Practice (GCP).
- Submitting data to the Coordinating Center.
- Registering all patients with the Coordinating Center by submitting patient registration form, and signed informed consent promptly.
- Providing sufficient experienced clinical and administrative staff and adequate facilities and equipment to conduct a collaborative trial according to the protocol.
- Maintaining regulatory binders on site and providing copies of all required documents to the Coordinating Center.

11.3 Meetings

Weekly to bi-weekly teleconferences of all investigators, research nurses and other study staff involved in the study will take place, starting once both sites have enrolled a subject. The following study team members involved with the conduct of the trial will be included as appropriate: study coordinators, data managers, research nurses, sub-investigators, collaborators (if applicable), and statistician.

During these meetings matters related to the following will be discussed: enrollment rate relative to expectation, characteristics of participants, retention of participants, adherence to protocol (potential or real protocol violations), validity and integrity of the data, toxicities, acquisition of serum samples and transfer to lab, and progress of data for objectives.

11.4 Monitoring

Evaluation of safety will be monitored continuously through day 100 following the last dose of nivolumab. The evaluations will be conducted under the direction of Dr. Vincent Lam and the study statistician; additional information may be found in the statistical section.

RedCap will be used for Data Analysis purposes by the Johns Hopkins Biostats team, but it will not contain CRFs.

12 Administrative Procedures

12.1 Protocol Amendments

Any changes to the protocol will be made in the form of an amendment and must be approved by the IRB before implementation. The Principal Investigator is responsible for the coordination and development of all protocol amendments.

12.2 Informed Consent

An investigator will explain to each subject the nature of the study, its purpose, procedures involved, expected duration, potential risks and benefits. Each subject will be informed that participation in the study is voluntary and that he/she may withdraw from the study at any time, and that withdrawal of consent will not affect his/her subsequent medical treatment. This informed consent will be given by means of a standard written statement and will be submitted for IRB approval prior to use. No patient will enter the study before his/her informed consent has been obtained. In accordance with the Health Information Portability and Accountability Act (HIPAA), the written informed consent document (or a separate document to be given in conjunction with the consent document) will include a subject authorization to release medical information to the study sponsor and supporting agencies and/or allow these bodies, a regulatory authority, or Institutional Review Board access to subjects' medical information that includes all hospital records relevant to the study, including subjects' medical history.

12.3 Ethics and Good Clinical Practice

This study must be carried out in compliance with the protocol and Good Clinical Practice, as described in:

1. ICH Harmonized Tripartite Guidelines for Good Clinical Practice 1996.
2. US 21 Code of Federal Regulations dealing with clinical studies (including parts 50 and 56 concerning informed consent and IRB regulations).
3. Declaration of Helsinki, concerning medical research in humans (Recommendations Guiding Physicians in Biomedical Research Involving Human Subjects, Helsinki 1964, amended Tokyo 1975, Venice 1983, Hong Kong 1989, Somerset West 1996).

The investigator agrees to adhere to the instructions and procedures described in it and thereby to adhere to the principles of Good Clinical Practice that it conforms to.

12.4 Regulatory Authorities

12.4.1 Institutional Review Board

Information regarding study conduct and progress will be reported to the Institutional Review Board (IRB) per the current institutional standards of each participating center**.**

12.4.2 Food and Drug Administration (FDA)

Principal Investigator Responsibilities

The Protocol Chair is responsible for performing the following tasks:

• Coordinating, developing, submitting, and obtaining approval for the protocol as well as its subsequent amendments.
• Assuring that the correct version of the protocol is used.
• Taking responsibility for the overall conduct of the study and for monitoring the progress of the study.
• Reviewing and ensuring reporting of Serious Adverse Events (SAE).
• Reviewing data from all sites.

13 Statistical Considerations

13.1 Study Design and Endpoints

This study is a phase 1b trial evaluating the safety and feasibility of up to two neoadjuvant immunotherapy regimens (Nivolumab alone or Nivolumab+relatlimab) for operable stage II/III esophageal/gastroesphageal junction cancer. Safety and feasibility will be monitored continuously throughout the study.

The primary endpoint is the safety of neoadjuvant immunotherapy administration. Toxicity will be assessed by CTCAE version 4.0. Safety is measured through the proportion of evaluable patients whose worst adverse events of interest occurred within 100 days after the last dose of Nivolumab (or Nivolumab+relatlimab) or within 30 days after surgery, whichever is longer. Adverse events of interest include any grade 3 or 4 treatment-related (definitely, probably or possibly) pneumonitis and acute respiratory failure. In addition, we will closely and continuously monitor any treatment-related grade 5 AE.

The secondary endpoint is the feasibility of neoadjuvant immunotherapy administration. Feasibility is assessed through the proportion of eligible patients who proceed to surgery without substantial delay (more than 60 days) due to treatment-related reasons.

The exploratory endpoints include:

- Pathological complete response
- Selected pharmacodynamics markers
- Selected immune markers
- Recurrence-free survival, defined as the time from treatment initiation to disease recurrence or death due to any cause, whichever occurs first.
- Overall survival, defined as the time from treatment initiation to death due to any cause.

13.2 Sample size and Monitoring Plans

For each neoadjuvant regimen, we aim to accrue 16 evaluable patients. Evaluable patients are those who receive at least one dose of neo-adjuvant nivolumab (or nivolumab+relatlimab) administration and have complete toxicity follow-up through 100days after the end of concurrent chemo-radiation.

13.2.1 Early Stopping Plan for Safety

Based on the data from CROSS trial and a literature search, we assume the rate of grade 3 or 4 treatment-related pneumonitis and acute respiratory failure in the regimen of chemo-radiation and surgery alone is about 9%. Therefore, to minimize the risks of adding nivolumab (or nivolumab+ relatlimab) as neo-adjuvant therapy, safety will be monitored by a Bayesian stopping rule for the rate of grade 3 or 4 treatment-related pneumonitis and acute respiratory failure greater than 30% (three times of baseline toxicity rate). Specifically, the Bayesian toxicity monitoring rule that suspends the accrual anytime if the posterior probability of grade 3 or 4 treatment-related pneumonitis and acute respiratory failure being larger than 30% is 70% or higher. We assume a priori that the experimental regimens has an average risk around 25% and there is about 34% chance that the risk will be 25% or higher. This corresponds to a Beta(1,3) prior distribution. Table 12.1 summarizes the continuous stopping rule for the 16 evaluable patients for each regimen. For example, if 3 patients out of the first 6 or 4 out of the first 7 evaluable patients experience grade 3 or 4 treatment-related pneumonitis and acute respiratory failure, we will stop accrual. At any time if the stopping criterion is met, accrual to the trial will be temporarily suspended and the principle investigators and study team will review the toxicity data and recommend either modification or termination of the trial.

Table 12.1 Stopping rule for safety

# patients with AE	2	3	4	5	6	7
Out of total # evaluable patients	2-3	4-6	7-9	10-12	13-15	16

Table 12.2 summarizes the operating characteristics based on 5,000 simulations with 16 evaluable patients in terms of how frequent the study would stop based on the stopping rule under different hypothetical toxicity rates, as well as the average sample sizes.

Table 12.2 Operating characteristics of the stopping rule for safety

Underlying risk	0.15	0.20	0.25	0.30	0.35	0.40	0.50
% of time study stops	7%	15.2%	27.5%	42.1%	58.5%	70.9%	89.6%
Expected sample size	15.4	14.7	13.8	12.6	11.2	10.2	8.1

All Grade 3 and Grade 4 immune-related toxicities, with some exceptions (e.g., easily correctable endocrinopathies) and for Grade 3 and Grade 4 hematologic and non-hematologic toxicities, will also be monitored closely and continuously by the study team and investigators. Excessive occurrences (qualitatively comparing to what would be expected with standard of care chemoradiation) may also lead to early stopping.

<u>13.2.2 Early Stopping Plan for Grade 5 AEs</u>

We will also evaluate the rate of treatment-related grade 5 AE if it's greater than 10%. Specifically, the Bayesian toxicity monitoring rule that suspends the accrual anytime if the posterior probability of treatment-related grade 5 AE being larger than 10% is 70% or higher. We assume a priori that the experimental regimens has an average risk 5% and there is about 14% chance that the risk will be 10% or higher. This corresponds to a Beta(0.1,1.9) prior distribution. Table 12.3 summarizes the continuous stopping rule for the 16 evaluable patients for each regimen.

Table 12.3 Stopping rule for safety

# patients with grade 5 AE	1	2	3
Out of total # evaluable patients	1-3	4-10	11-16

Table 12.4 summarizes the operating characteristics based on 5,000 simulations with 16 evaluable patients in terms of how frequent the study would stop based on the stopping rule under different hypothetical grade 5 AE rates, as well as the average sample sizes.

Table 12.4 Operating characteristics of the stopping rule for grade 5 AE

Underlying risk	0.04	0.08	0.10	0.12	0.16	0.20
% of time study stops	6.4%	21.5%	32.2%	38.8%	57.5%	72.4%

Expected sample size	15.5	14.4	13.6	13.1	11.6	10.4

13.2.3 Early Stopping Plan for Feasibility

The feasibility of neoadjuvant nivolumab (or nivolumab+ relatlimab) will be based on the proportion of patients proceeding to surgery without substantial treatment related delays. Recovery from standard of care chemoradiation is different for each patient. For safety reasons, it is standard of care for surgeons to evaluate patients on a case by case basis. Based on our Johns Hopkins institutional experience, patients undergo surgery (Ivor-Lewis esophagectomy) in a range of 7-11 weeks following chemoradiation with no mortality recorded within 90 days after surgery. Therefore, in this study a treatment related delay will be considered "substantial" if it is greater than 11 weeks following chemoradiation. We would consider the experimental regimen is "infeasible" if the probability of not proceeding to surgery as planned and delayed is more than .25 (with more than 80% posterior probability), i.e., the probability of proceeding to surgery as planned is less than 75%. We assume at most 10% of patients will have their surgery delayed and there is about 12% chance that the risk will be 25% or more. This corresponds to a Beta(0.5,4.5) prior distribution. Table 12.5 summarizes the continuous stopping rule for the 16 evaluable patients for each regimen. The feasibility stopping rule calls for the study to be paused for a review if the number of patients successfully proceeding to surgery is too low, starting from the 6th evaluable patient.

Table 12.5 Stopping rule for feasibility

# patients with surgery delayed	4	5	6	7
Out of total # evaluable patients	6-7	8-10	11-13	14-16

Table 12.6 summarizes the operating characteristics based on 5,000 simulations with 16 evaluable patients in terms of how frequent the study would stop based on the stopping rule under different hypothetical feasibility rates, as well as the average sample sizes.

Table 12.6 Operating characteristics of the stopping rule for feasibility

Underlying feasibility	0.65	0.7	0.75	0.8	0.85	0.9
% of time study stops	41%	26.5%	14.1%	6.1%	2%	0.5%
Expected sample size	13.2	14.3	15	15.6	15.8	16

13.3 Statistical Analysis Plans

To minimize the potential risks exposed to patients, the safety and feasibility related analyses for Arm A will be conducted prior to initiating accruals for Arm B.

Adverse events for each regimen will be tabulated by type, grade, and attribution of adverse event. In addition, the proportions of grade 3 or 4 treatment-related pneumonitis and acute respiratory failure, treatment-related grade 5 AE, and patients with surgery without substantial delays (more than 12 weeks) will be reported along with exact binomial 95% confidence intervals.

For all exploratory endpoints, descriptive analysis but no formal hypothesis testing will be performed given the nature of exploratory analysis. To preliminarily assess the efficacy of the experimental regimen, pathological complete response rate will be estimated among all evaluable patients, and 95% exact confidence interval will be provided. Recurrence-free survival is defined as the time from treatment initiation to disease recurrence or death due to any cause, whichever occurs first. Overall survival is defined as the time from treatment initiation to death due to any cause. Both recurrence-free survival and overall survival will be analyzed as time-to-event data, i.e., the respective rates at different time-points, e.g., every 6 months, will be estimated using Kaplan-Meier method, and the associated point-wise confidence interval will be calculated using Greenwood formula with log-log transformation. Parameters of pharmacodynamics markers and immune markers will be summarized with descriptive statistics. These summaries will be computed for each evaluable patient at multiple timepoints before and after neoadjuvant regimen is administered. Plots will be used to show the changes in immune response over time both for each individual. For each patient, comparisons in the pre and post-nivolumab responses will be compared using paired t-tests (or Wilcoxon signed rank tests if appropriate) for continuous variables and McNemar's test for dichotomous or categorical variables. Associations between immune responses will be explored graphically (e.g. scatterplots, boxplots) and numerically (e.g. correlations, $\chi 2$ tests). All secondary and exploratory endpoints will be analyzed at the same time or after the primary endpoint analysis.

Appendix A: TNM staging system for esophageal cancer (7th edition)

ESOPHAGUS STAGING FORM

CLINICAL<br>*Extent of disease before any treatment*	STAGE CATEGORY DEFINITIONS		PATHOLOGIC<br>*Extent of disease through completion of definitive surgery*
❒ y clinical– staging completed after neoadjuvant therapy but before subsequent surgery	TUMOR SIZE:__________	LATERALITY:<br>☐ left ☐ right ☐ bilateral	❒ y pathologic – staging completed after neoadjuvant therapy AND subsequent surgery
	**PRIMARY TUMOR (T)**		
❑ TX	Primary tumor cannot be assessed		❑ TX
❑ T0	No evidence of primary tumor		❑ T0
❑ Tis	High-grade dysplasia *		❑ Tis
❑ T1	Tumor invades lamina propria, muscularis mucosae, or submucosa		❑ T1
❑ T1a	Tumor invades lamina propria or muscularis mucosae		❑ T1a
❑ T1b	Tumor invades submucosa		❑ T1b
❑ T2	Tumor invades muscularis propria		❑ T2
❑ T3	Tumor invades adventitia		❑ T3
❑ T4	Tumor invades adjacent structures		❑ T4
❑ T4a	Resectable tumor invading pleura, pericardium, or diaphragm		❑ T4a
❑ T4b	Unresectable tumor invading other adjacent structures, such as aorta, vertebral body, trachea, etc.		❑ T4b
	*High-grade dysplasia includes all non-invasive neoplastic epithelium that was formerly called carcinoma *in situ*, a diagnosis that is no longer used for columnar mucosae anywhere in the gastrointestinal tract.		
	**REGIONAL LYMPH NODES (N)**		
❑ NX	Regional lymph nodes cannot be assessed		❑ NX
❑ N0	No regional lymph node metastasis		❑ N0
❑ N1	Regional lymph node metastases involving 1 to 2 nodes		❑ N1
❑ N2	Regional lymph node metastases involving 3 to 6 nodes		❑ N2
❑ N3	Regional lymph node metastases involving 7 or more nodes		❑ N3
	**DISTANT METASTASIS (M)**		
❑ M0	No distant metastasis (no pathologic M0; use clinical M to complete stage group)		
❑ M1	Distant metastasis		❑ M1

Table 2 AJCC 7th edition stage groupings

Stage	Adenocarcinoma				Squamous cell carcinoma				
	T	N	M	Grade	T	N	M	G	Location
0	is	0	0	1	is	0	0	1	Any
IA	1	0	0	1-2	1	0	0	1	Any
IB	1	0	0	3	1	0	0	2-3	Any
	2	0	0	1-2	2-3	0	0	1	Lower
IIA	2	0	0	3	2-3	0	0	1	Upper, middle
					2-3	0	0	2-3	Lower
IIB	3	0	0	Any	2-3	0	0	2-3	Upper, middle
	1-2	1	0	Any	1-2	1	0	Any	Any
IIIA	1-2	2	0	Any	1-2	2	0	Any	Any
	3	1	0	Any	3	1	0	Any	Any
	4a	0	0	Any	4a	0	0	Any	Any
IIIB	3	2	0	Any	3	2	0	Any	Any
IIIC	4a	1-2	0	Any	4a	1-2	0	Any	Any
	4b	Any	0	Any	4b	Any	0	Any	Any
	Any	3	0	Any	Any	3	0	Any	Any
IV	Any	Any	1	Any	Any	Any	1	Any	Any

Cancer location definitions: upper thoracic, 20-25 cm from incisors; middle thoracic, 25-30 cm from incisors; lower thoracic, 30-40 cm from incisors.

Appendix B: ECOG Performance Status Scale

Score	Definition
0	Asymptomatic
1	Symptomatic, fully ambulatory
2	Symptomatic, in bed less than 50% of day
3	Symptomatic, in bed more than 50% of day, but not bedridden
4	Bedridden

Appendix C: Guidelines for Tissue Banking Process to be modified from lung cancer to esophageal cancer.

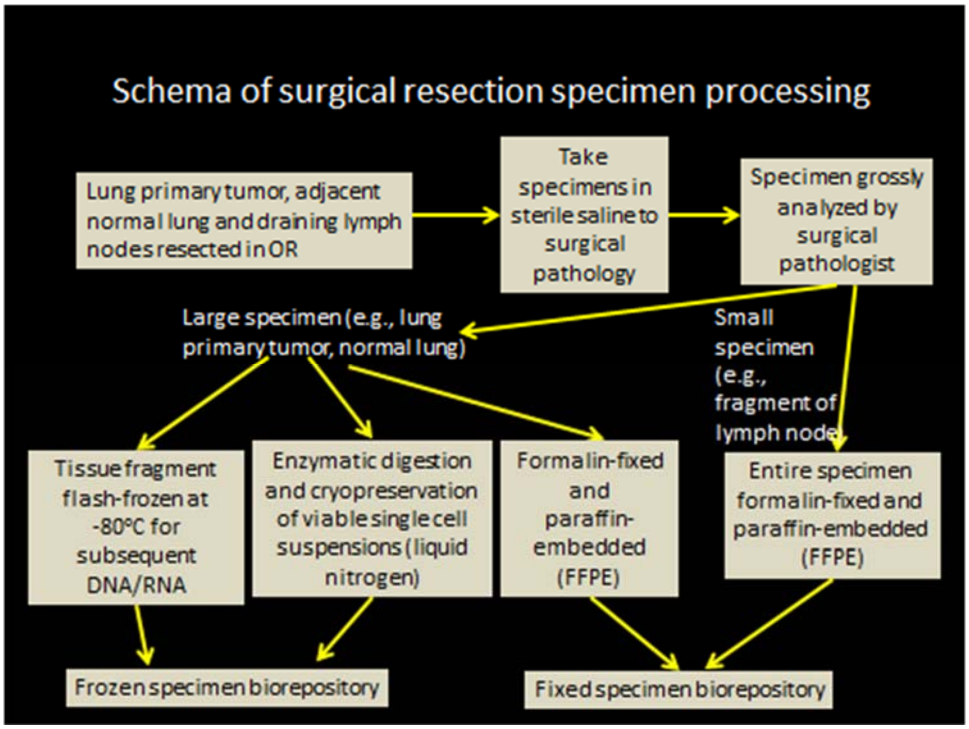

Note: Only tissue that is absolutely not needed for clinical diagnosis or staging should be collected for tissue banking**. If in doubt about this, do NOT submit specimens for banking.**

Banking of Frozen Tissue

1. Place a single tissue specimen flat in the plastic bag. A single tissue specimen's overall volume should be at least 1 cm3, and at most 3 – 4 cm3, with at least one dimension measuring 0.5 cm thick or less to facilitate quick freezing.

2. For a given case (patient), please collect sufficient **non-malignant and malignant** tissue. Tissue selected should be grossly viable, and grossly consistent with tumor or adjacent normal tissue (see #5 below for contraindications). Non-malignant (i.e. "adjacent normal") tissue should be collected at least **2 cm** from the primary tumor, subject to any limitations from the specimen's physical dimensions. Do not place tumor and non-malignant tissue in the same bag. For large tumors, do not place large pieces of tissue in a single bag. Rather, divide the tissue according to size guidelines in #1 above, and place each in an individual bag. Collect and separately identify both: 1) primary tumor and 2) metastatic lesions to lymph nodes or other tissues. Tissue will typically be taken by scalpel or dissection blade, though the use of 5 -7 mm skin punch biopsy tools could be considered in certain situations.

3. Immediately place the specimens for freezing in an isopentane or 2-methylbutane cryobath, or other effective liquid freezing agent. If no cryobath is available, then liquid N2 can be used as the freezing agent, in a properly insulated container and with sufficient safety precautions. The goal is to have bankable tissue immersed in the bath **within 30 minutes of the OR's procurement** from the patient. If more cryobath space is needed, move **already frozen** tissue to a -80C freezer in order to make sufficient room. Make sure to check periodically for cryobath problems (e.g. not maintaining temperature, refrigerant level low), and call for appropriate maintenance as needed. **Do not freeze tissue by placing it fresh directly in the -80C freezer.**

4. On receipt by the tissue bank laboratory, the frozen tissue is embedded in OCT (Optimal Cutting Temperature medium), and a frozen section is stained with H&E and the section evaluated by the tissue bank pathologist for quality assurance (QA) purposes. A report on the histopathologic findings is filed or communicated as needed. The frozen section evaluation can also count for adjacent pieces of tissue if they were taken as a "mirror image" section to the surface cut for the frozen section.

5. General contraindications to tissue banking

DON'T bank tissue from these specimen types or situations:

- small tumors and other cases where all or most of the lesional tissue is needed for diagnosis
- surgical margins of resection specimens where tumor and benign areas cannot be clearly delineated grossly grossly visible areas of primarily necrosis, hemorrhage, or fat
- specimens which are known to have been delayed significantly more than 30 minutes past their procurement time in the OR
- tissue previously freeze-thawed, or frozen slowly (e.g. in the cryostat or -80 freezer)
- areas of deepest invasion, tumor/normal interface, tumor/capsule interface, extranodal extension of tumor, and other key landmarks needed for surgical pathology evaluation and/or tumor staging
- chemotherapy- or radiation-treated tumors
- diagnostic biopsies where most or all tissue must be submitted for pathology evaluation…most lymph node, GI, bone marrow, and liver biopsies fall in this category
- tissue clearly marked as intended for a special study such as immunofluorescence

Appendix D: MANAGEMENT ALGORITHMS

These general guidelines constitute guidance to the Investigator and may be supplemented by discussions with the Principal Investigator. The guidance applies to all immuno-oncology agents and regimens.

A general principle is that differential diagnoses should be diligently evaluated according to standard medical practice. Non-inflammatory etiologies should be considered and appropriately treated.

Corticosteroids are a primary therapy for immuno-oncology drug-related adverse events. The oral equivalent of the recommended IV doses may be considered for ambulatory patients with low-grade toxicity. The lower bioavailability of oral corticosteroids should be taken into account when switching to the equivalent dose of oral corticosteroids.

Consultation with a medical or surgical specialist, especially prior to an invasive diagnostic or therapeutic procedure, is recommended.

The frequency and severity of the related adverse events covered by these algorithms will depend On the immuno-oncology agent or regimen being used.

GI Adverse Event Management Algorithm

Rule out non-inflammatory causes. If non-inflammatory cause is identified, treat accordingly and continue I-O therapy. Opiates/narcotics may mask symptoms of perforation. Infliximab should not be used in cases of perforation or sepsis.

Grade of Diarrhea/ Colitis (NCI CTCAE v4)	Management	Follow-up
**Grade 1**<br>Diarrhea: < 4 stools/day over baseline;<br>Colitis: asymptomatic	• Continue I-O therapy per protocol<br>• Symptomatic treatment	• Close monitoring for worsening symptoms.<br>• Educate patient to report worsening immediately<br>If worsens:<br>• Treat as Grade 2 or 3/4
**Grade 2**<br>Diarrhea: 4-6 stools per day over baseline; IV fluids indicated <24 hrs; not interfering with ADL<br>Colitis: abdominal pain; blood in stool	• Delay I-O therapy per protocol<br>• Symptomatic treatment	If improves to grade 1:<br>• Resume I-O therapy per protocol<br>If persists > 5-7 days or recur:<br>• 0.5-1.0 mg/kg/day methylprednisolone or oral equivalent<br>• When symptoms improve to grade 1, taper steroids over at least 1 month, consider prophylactic antibiotics for opportunistic infections, and resume I-O therapy per protocol.<br>If worsens or persists > 3-5 days with oral steroids:<br>• Treat as grade 3/4
**Grade 3-4**<br>Diarrhea (G3): ≥7 stools per day over baseline; incontinence; IV fluids ≥24 hrs; interfering with ADL<br>Colitis (G3): severe abdominal pain, medical intervention indicated, peritoneal signs<br>G4: life-threatening, perforation	• Discontinue I-O therapy per protocol<br>• 1.0 to 2.0 mg/kg/day methylprednisolone IV or IV equivalent<br>• Add prophylactic antibiotics for opportunistic infections<br>• Consider lower endoscopy	If improves:<br>• Continue steroids until grade 1, then taper over at least 1 month<br>If persists > 3-5 days, or recurs after improvement:<br>• Add infliximab 5 mg/kg (if no contraindication). Note: Infliximab should not be used in cases of perforation or sepsis

Patients on IV steroids may be switched to an equivalent dose of oral corticosteroids (e.g. prednisone) at start of tapering or earlier, once sustained clinical improvement is observed. Lower bioavailability of oral corticosteroids should be taken into account when switching to the equivalent dose of oral corticosteroids.

Renal Adverse Event Management Algorithm

Rule out non-inflammatory causes. If non-inflammatory cause, treat accordingly and continue I-O therapy

Grade of Creatinine Elevation (NCI CTCAE v4)	Management	Follow-up
**Grade 1**<br>Creatinine > ULN and > than baseline but ≤ 1.5x baseline	• Continue I-O therapy per protocol<br>• Monitor creatinine weekly	<u>If returns to baseline</u>:<br>•Resume routine creatinine monitoring per protocol<br><u>If worsens:</u><br>•Treat as Grade 2 or 3/4
**Grade 2-3**<br>Creatinine > 1.5x baseline to ≤ 6x ULN	• Delay I-O therapy per protocol<br>• Monitor creatinine every 2-3 days<br>• 0.5 to 1.0 mg/kg/day methylprednisolone IV or oral equivalent<br>• Consider renal biopsy with nephrology consult	<u>If returns to Grade 1:</u><br>•Taper steroids over at least 1 month, consider prophylactic antibiotics for opportunistic infections, and resume I-O therapy and routine creatinine monitoring per protocol<br><br><u>If elevations persist > 7 days or worsen:</u><br>•Treat as Grade 4
**Grade 4**<br>Creatinine > 6x ULN	• Discontinue I-O therapy per protocol<br>• Monitor creatinine daily<br>• 1.0-2.0 mg/kg/day methylprednisolone IV or IV equivalent<br>• Consult nephrologist<br>• Consider renal biopsy	<u>If returns to Grade 1</u>: Taper steroids over at least 1 month and add prophylactic antibiotics for opportunistic infections

Patients on IV steroids may be switched to an equivalent dose of oral corticosteroids (e.g. prednisone) at start of tapering or earlier, once sustained clinical improvement is observed. Lower bioavailability of oral corticosteroids should be taken into account when switching to the equivalent dose of oral corticosteroids.

Pulmonary Adverse Event Management Algorithm

Rule out non-inflammatory causes. If non-inflammatory cause, treat accordingly and continue I-O therapy. Evaluate with imaging and pulmonary consultation.

Grade of Pneumonitis (NCI CTCAE v4)	Management	Follow-up
**Grade 1**<br>Radiographic changes only	• Consider delay of I-O therapy<br>• Monitor for symptoms every 2-3 days<br>• Consider Pulmonary and ID consults	• Re-image at least every 3 weeks<br>__If worsens:__<br>• Treat as Grade 2 or 3-4
**Grade 2**<br>Mild to moderate new symptoms	• Delay I-O therapy per protocol<br>• Pulmonary and ID consults<br>• Monitor symptoms daily, consider hospitalization<br>• 1.0 mg/kg/day methyl-prednisolone IV or oral equivalent<br>• Consider bronchoscopy , lung biopsy	• Re-image every 1-3 days<br>__If improves:__<br>• When symptoms return to near baseline, taper steroids over at least 1 month and then resume I-O therapy per protocol and consider prophylactic antibiotics<br>__If not improving after 2 weeks or worsening:__<br>• Treat as Grade 3-4
**Grade 3-4**<br>Severe new symptoms; New/worsening hypoxia; Life-threatening	• Discontinue I-O therapy per protocol<br>• Hospitalize<br>• Pulmonary and ID consults<br>• 2-4 mg/kg/day methylprednisolone IV or IV equivalent<br>• Add prophylactic antibiotics for opportunistic infections<br>• Consider bronchoscopy, lung biopsy	__If improves to baseline:__<br>• Taper steroids over at least 6 weeks<br>__If not improving after 48 hours or worsening:__<br>• Add additional immunosuppression

Patients on IV steroids may be switched to an equivalent dose of oral corticosteroids (e.g. prednisone) at start of tapering or earlier, once sustained clinical improvement is observed. Lower bioavailability of oral corticosteroids should be taken into account when switching to the equivalent dose of oral corticosteroids.

Hepatic Adverse Event Management Algorithm

Rule out non-inflammatory causes. If non-inflammatory cause, treat accordingly and continue I-O therapy. Consider imaging for obstruction.

Grade of Liver Test Elevation (NCI CTCAE v4)	Management	Follow-up
**Grade 1**<br>AST or ALT > ULN to 3.0 x ULN and/or T. bili > ULN to 1.5 x ULN	• Continue I-O therapy per protocol	• Continue LFT monitoring per protocol<br>If worsens:<br>• Treat as Grade 2 or 3-4
**Grade 2**<br>AST or ALT > 3.0 to ≤ 5 x ULN and/or T. bili > 1.5 to ≤ 3 x ULN	• Delay I-O therapy per protocol<br>• Increase frequency of monitoring to every 3 days	If returns to baseline:<br>•Resume routine monitoring, resume I-O therapy per protocol<br>If elevations persist > 5-7 days or worsen :<br>• 0.5-1 mg/kg/day methylprednisolone or oral equivalent and when LFT returns to grade 1 or baseline, taper steroids over at least 1 month, consider prophylactic antibiotics for opportunistic infections, and resume I-O therapy per protocol
**Grade 3-4**<br>AST or ALT > 5 x ULN or T.bili >3 x ULN	• Discontinue I-O therapy*<br>• Increase frequency of monitoring to every 1-2 days<br>• 1.0 to 2.0 mg/kg/day methylprednisolone IV or IV equivalent**<br>• Add prophylactic antibiotics for opportunistic infections<br>• Consult gastroenterologist	If returns to grade 2:<br>• Taper steroids over at least 1 month<br>If does not improve in >3-5 days, worsens or rebounds:<br>• Add mycophenolate mofetil 1 g BID<br>• If no response within an additional 3-5 days, consider other immunosuppressants per local guidelines

Patients on IV steroids may be switched to an equivalent dose of oral corticosteroids (e.g. prednisone) at start of tapering or earlier, once sustained clinical improvement is observed. Lower bioavailability of oral corticosteroids should be taken into account when switching to the equivalent dose of oral corticosteroids.

***I-O therapy may be delayed rather than discontinued if AST/ALT ≤ 8 x ULN or T.bili ≤ 5 x ULN.**

**The recommended starting dose for grade 4 hepatitis is 2 mg/kg/day methylprednisolone IV.

Endocrinopathy Management Algorithm

Rule out non-inflammatory causes. If non-inflammatory cause, treat accordingly and continue I-O therapy. Consider visual field testing, endocrinology consultation, and imaging.

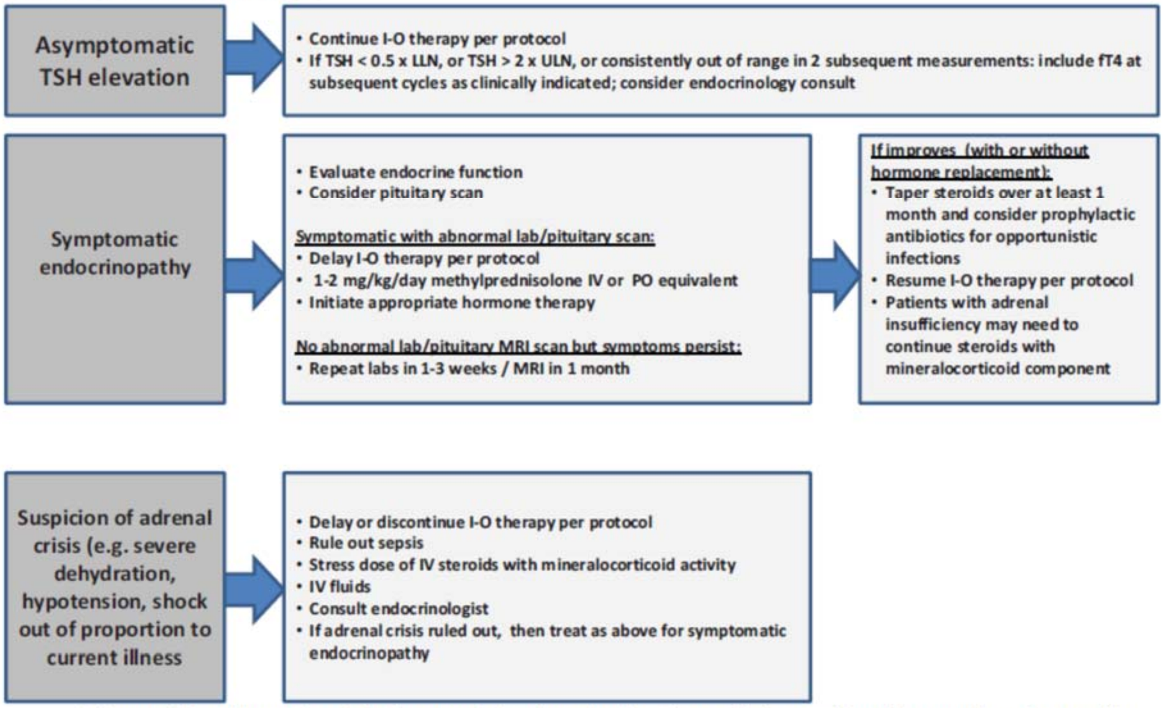

Patients on IV steroids may be switched to an equivalent dose of oral corticosteroids (e.g. prednisone) at start of tapering or earlier, once sustained clinical improvement is observed. Lower bioavailability of oral corticosteroids should be taken into account when switching to the equivalent dose of oral corticosteroids.

Skin Adverse Event Management Algorithm

Rule out non-inflammatory causes. If non-inflammatory cause, treat accordingly and continue I-O therapy.

Grade of Rash (NCI CTCAE v4)	Management	Follow-up
**Grade 1-2**<br>Covering ≤ 30% BSA*	• Symptomatic therapy (e.g. antihistamines, topical steroids)<br>• Continue I-O therapy per protocol	<u>If persists > 1-2 weeks or recurs:</u><br>• Consider skin biopsy<br>• Delay I-O therapy per protocol<br>• Consider 0.5-1.0 mg/kg/day methylprednisolone IV or oral equivalent. Once improving , taper steroids over at least 1 month, consider prophylactic antibiotics for opportunistic infections, and resume I-O therapy per protocol<br><u>If worsens:</u><br>• Treat as Grade 3-4
**Grade 3-4**<br>Covering >30% BSA; Life threatening consequences*^	• Delay or discontinue I-O therapy per protocol<br>• Consider skin biopsy<br>• Dermatology consult<br>• 1.0-2.0 mg/kg/day IV methylprednisolone IV or IV equivalent	<u>If improves to Grade 1:</u><br>• Taper steroids over at least 1 month and add prophylactic antibiotics for opportunistic infections<br>• Resume I-O therapy per protocol

Patients on IV steroids may be switched to an equivalent dose of oral corticosteroids (e.g. prednisone) at start of tapering or earlier, once sustained clinical improvement is observed. Lower bioavailability of oral corticosteroids should be taken into account when switching to the equivalent dose of oral corticosteroids.
*Refer to NCI CTCAE v4 for term-specific grading criteria.
^If SJS/TEN is suspected, withhold I-O therapy and refer patient for specialized care for assessment and treatment. If SJS or TEN is diagnosed, permanently discontinue I-O therapy.

Neurological Adverse Event Management Algorithm

Rule out non-inflammatory causes. If non-inflammatory cause, treat accordingly and continue I-O therapy.

Grade of Neurological Toxicity (NCI CTCAE v4)	Management	Follow-up
**Grade 1** Asymptomatic or mild symptoms; Intervention not indicated	• Continue I-O therapy per protocol	Continue to monitor the patient. <u>If worsens:</u> • Treat as Grade 2 or 3-4
**Grade 2** Moderate symptoms; Limiting instrumental ADL	• Delay I-O therapy per protocol • Treat symptoms per local guidelines • Consider 0.5 to 1.0 mg/kg/day methylprednisolone IV or PO equivalent	<u>If improves to baseline</u>: •Resume I-O therapy per protocol when improved to baseline <u>If worsens</u>: • Treat as Grade 3-4
**Grade 3-4** Severe symptoms; Limiting self-care ADL; Life-threatening	• Discontinue I-O therapy per protocol • Obtain neurology consult • Treat symptoms per local guidelines • 1.0-2.0 mg/kg/day IV methylprednisolone IV or IV equivalent • Add prophylactic antibiotics for opportunistic infections	<u>If improves to Grade 2</u>: • Taper steroids over at least 1 month <u>If worsens or atypical presentation:</u> • Consider IVIG or other immunosuppressive therapies per local guidelines

Patients on IV steroids may be switched to an equivalent dose of oral corticosteroids (e.g. prednisone) at start of tapering or earlier, once sustained clinical improvement is observed. Lower bioavailability of oral corticosteroids should be taken into account when switching to the equivalent dose of oral corticosteroids.

APPENDIX E: Initial Sociodemographic Characteristics Questionnaire

Date: Month_____ / Day ______ / Year______

Study ID Number: ______________________

INITIAL SOCIODEMOGRAPHIC CHARACTERISTICS QUESTIONNAIRE
FAMILY QUESTIONS

Both genetics and environment could be risk factors for the development of cancer. For this reason, it is important to determine your biological relationship with your family.

1. Are you adopted? □ Yes □ No

2. How many of each of the following family members do you have?

Brothers: __________Sisters: __________Sons: __________ Daughters: _________

SMOKING QUESTIONS

3. Do you smoke cigarettes? □ Yes □ No, never

□ Not currently but I have in the past

4. At what age did you start smoking? _________

5. When did you quit smoking cigarettes? _________

6. How many total years have you or did you regulary smoke cigarettes?

_____________ _________

7. During the time you usually smoked regularly, how many cigarettes do or did you usually smoke per day? _________

8. Do you smoke cigars? □ Yes □ No, never □ Not currently but I have in the Past

9. At what age did you start smoking cigars? ________

10. When did you quit smoking cigars? _________

11. How many years in total did you regularly smoke cigars? ___________

12 Do you use smokeless tobacco or other nicotine products? (i.e. chewing tobacco, snuff, e-cigarette, nicotine patch or gum) □ Yes □ No

If yes, please indicate type(s): ____

13. Were you exposed to asbestos, that you know of? □ Yes □ No, never

14. Were you exposed to any other potential harmful exposures to your lung? □ Yes □ No, never

ALCOHOL QUESTIONS

15. Have you ever drunk alcoholic beverages, such as beer, wine, or liquor regularly, that is at least once a month? □ Yes □ No

16. At what age did you start drinking alcoholic beverages regularly, i.e. at least once a month?

__________________years of age

17. Before the age of 40, how many drinks of beer (12 oz.), wine (5oz.), or liquor (1 oz.) did you usually drink per week?

More than one per week. Please indicate number_________________

Less than one per week_________________

Never drank before age 40 _________

18. After the age of 40, how many drinks of beer (12 oz.), wine (5 oz.), or liquor (1 oz.) did you usually drink per week?

More than one per week. Please indicate number ________________

Less than one per week ___________

Never drank after age 40 __________

Currently aged less than 40 years __________

MEDICAL QUESTIONS

19. Have you taken antibiotics in the last 3 months? ☐ Yes ☐ No

A list of antibiotics is attached for you to refer to. If no, skip to question 25.

20. If you know the name of the antibiotic(s), please write it here.

____

____

21. Have you had a bronchoscopy in the last 3 months? ☐ Yes ☐ No

22Have you taken oral corticosteroids in the last 2 weeks? Yes No

(Oral corticosteroids examples: prednisone, dexamethasone, methlyprednisone, hydrocortisone)

23. If you answered Yes to (26), please write the name and dose here and when you took these.

____

____

24. Have you taken inhaled corticosteroids in the last 2 weeks? Yes No

(Inhaled corticosteroids examples: budesonide, fluticasone, beclomethasone, ciclesonide)

25. If you answered Yes to (28), please write the name and dose here and when you took these.

____

____

26.Do you have sleep apnea? Yes No

27Do you have reflux disease? Yes No

28.Do you have any other chronic lung conditions? Yes No

29. If you answered Yes to (32), please write the name of the condition here

____

____

DENTAL HEALTH

30. About how often do you visit a dentist?

- ☐ **Less than every 6 months.**
- ☐ **Between every 6-12 months.**
- ☐ **Greater than every 12 months.**
- ☐ **I have never been to a dentist.**

31. Overall, how would you rate the health of your teeth and gums?
☐ **Excellent** ☐ **Good** ☐ **Fair**
☐ **Poor**

32. Have you ever had treatment for gum disease such as scaling and root planing, sometimes called deep cleaning?
☐ **Yes** ☐ **No**

33. Have you ever had any teeth become loose on their own, without an injury? (Not including baby teeth). ☐ **Yes**
☐ **No**

DIET QUESTIONS

34. Do you eat meat? Meat is defined as beef, chicken, pork, lamb or venison:

A. I do not eat meat. ☐

B. I have eaten meat in the last year. ☐

C. I have eaten meat one or more times a month during the last year. ☐

D. I have eaten meat one or more times a week during the last year. ☐

35. Do you eat Fish? Fish is defined as all fish and shellfish:

A. I do not eat fish. ☐

B. I have eaten fish in the last year. ☐

C. I have eaten fish one or more times a month during the last year. ☐

D. I have eaten fish one or more times a week during the last year ☐

36. Do you eat Eggs?

A. I do not eat eggs ☐

B. I have eaten eggs in the last year ☐

C. I have eaten eggs one or more times a month during the last year ☐

D. I have eaten eggs one or more times a week during the last year ☐

37. Do you eat Cheese? (This includes fresh, soft, aged or cottage cheese as well as sour cream):

A. I do not eat cheese ☐

B. I have eaten cheese in the last year ☐

C. I have eaten cheese one or more times a month during the last year ☐

D. I have eaten cheese one or more times a week during the last year ☐

38. Do you drink Milk? Milk is defined as milk from a cow, goat or sheep (not soy, coconut or almond milk, for example). If you put milk on your cereal, you should not answer (A).

A. I do not drink milk ☐

B. I drank milk in the last year ☐

C. I drank milk one or more times a month during the last year ☐

D. I drank milk one or more times a week during the last year ☐

39. Do you eat yogurt?

A. I do not eat yogurt ☐

B. I have eaten yogurt in the last year ☐

C. I have eaten yogurt one or more times a month during the last year ☐

D. I have eaten yogurt one or more times a week during the last year ☐

40. Do you take probiotics (live bacteria supplement)? ☐ **Yes** ☐ **No**

If yes (question 44) and if you know the name of the probiotic product(s), please write here:

____

____

41. Do you take vitamin supplements? ☐ **Yes** ☐ **No**

If yes (question 45) and if you know the name of the vitamin supplement(s), please write it/them here:

____

____

WORK AND PHYSICAL ACTIVITY

42. What is your current employment status?

□ **Employed/self-employed** □ **Unemployed** □ **Retired** □ **Disabled**

43. How would you categorize your physical activity on the job?

□ Mostly sedentary or light activity (e.g. mostly sitting, standing, lifting light objects of less than 3 kilos).

□ Mostly medium activity (e.g. much walking, climbing stairs).

□ Mostly intense activity (e.g. heavy construction work).

□ Unemployed/retired/disabled

44. What type of exercise (physical activity) do you do regularly (at least 3 times per week)?

□ Mostly moderate activity (slow walking, gardening, golfing etc.)

□ Mostly vigorous activity (running, swimming, bicycling, football etc.)

□ I do not exercise regularly

45. At age 20, what type of exercise did you do regularly (at least 3 times per week)?

□ Mostly moderate activity (slow walking, gardening, golfing etc.)

□ Mostly vigorous activity (running, swimming, bicycling, football etc.)

□ I did not exercise regularly

APPENDIX F: Follow-up Sociodemographic Characteristics Questionnaire

Visit Date: Month_____ / Day ______ / Year______

Study ID Number: ______________________

FOLLOW-UP SOCIODEMOGRAPHIC CHARACTERISTICS QUESTIONNAIRE

SMOKING QUESTIONS

1. Are you currently smoking? ☐ Yes ☐ No

If so, how many cigarettes/day? _________

ALCOHOL QUESTIONS

1. Are you currently consuming alcoholic beverages such as wine, beer or liquor regularly?

☐ Yes ☐ No

If yes, please indicate pattern:
More than 1 per week. ________ If checked, please indicate number_______
Less than 1 per week_________

MEDICAL QUESTIONS

1. Have you taken antibiotics since completing the last questionnaire? ☐ Yes ☐ No (Skip to question 3)

A list of antibiotics is attached for you to refer to.

2. If you know the name of the antibiotic(s), please write it here. ________________________

____

3. Have you had a bronchoscopy since completing the last questionnaire? ☐ Yes ☐ No

4. Have you taken oral corticosteroids since completing the last questionnaire? ☐ Yes ☐ No (Skip to question 6)

(Oral corticosteroids examples: prednisone, dexamethasone, methlyprednisone, hydrocortisone)

5. If you answered Yes to (4), please write the name and dose and when you took these.

____

____

6. Have you taken inhaled corticosteroids since completing the last questionnaire? ☐ Yes ☐ No (Skip to Part IV)
(Inhaled corticosteroids examples: budesonide, fluticasone, beclomethasone, ciclesonide)

7. If you answered Yes to (6), please write the name and dose and when you took these.

____

____

DIET QUESTIONS

1. Have you changed your diet since completing the last questionnaire?

☐ Yes ☐ No

If yes, please answer questions 2-9 below.

2. Do you eat meat? Meat is defined as beef, chicken, pork, lamb or venison:
   A. I do not eat meat. ☐
   B. I have eaten meat in the last year. ☐
   C. I have eaten meat one or more times a month during the last year. ☐
   D. I have eaten meat one or more times a week during the last year. ☐

3. Do you eat Fish? Fish is defined as all fish and shellfish:
   A. I do not eat fish. ☐
   B. I have eaten fish in the last year. ☐
   C. I have eaten fish one or more times a month during the last year. ☐
   D. I have eaten fish one or more times a week during the last year ☐

4. Do you eat Eggs?
   A. I do not eat eggs ☐
   B. I have eaten eggs in the last year ☐

C. I have eaten eggs one or more times a month during the last year ☐
D. I have eaten eggs one or more times a week during the last year ☐

5. Do you eat Cheese? (This includes fresh, soft, aged or cottage cheese as well as sour cream):
   A. I do not eat cheese ☐
   B. I have eaten cheese in the last year ☐
   C. I have eaten cheese one or more times a month during the last year ☐
   D. I have eaten cheese one or more times a week during the last year ☐

6. Do you drink Milk? Milk is defined as milk from a cow, goat or sheep (not soy, coconut or almond milk, for example). If you put milk on your cereal, you should not answer (A).
   A. I do not drink milk ☐
   B. I drank milk in the last year ☐
   C. I drank milk one or more times a month during the last year ☐
   D. I drank milk one or more times a week during the last year ☐

7. Do you eat yogurt?
   A. I do not eat yogurt ☐
   B. I have eaten yogurt in the last year ☐
   C. I have eaten yogurt one or more times a month during the last year ☐
   D. I have eaten yogurt one or more times a week during the last year ☐

8. Do you take probiotics (live bacteria supplement)? ☐ Yes ☐ No

If yes (question 8) and if you know the name of the probiotic product(s), please write it/them here:

9. Do you take vitamin supplements? ☐ Yes ☐ No

If yes (question 9) and if you know the name of the vitamin supplement(s), please write it/them here:

____

References

1. van Hagen, P., et al., *Preoperative chemoradiotherapy for esophageal or junctional cancer.* N Engl J Med, 2012. **366**(22): p. 2074-84.
2. Stahl, M., et al., *Phase III comparison of preoperative chemotherapy compared with chemoradiotherapy in patients with locally advanced adenocarcinoma of the esophagogastric junction.* J Clin Oncol, 2009. **27**(6): p. 851-6.
3. Lordick, F., et al., *PET to assess early metabolic response and to guide treatment of adenocarcinoma of the oesophagogastric junction: the MUNICON phase II trial.* Lancet Oncol, 2007. **8**(9): p. 797-805.
4. Moons, L.M., et al., *Barrett's oesophagus is characterized by a predominantly humoral inflammatory response.* J Pathol, 2005. **207**(3): p. 269-76.
5. Fitzgerald, R.C., et al., *Inflammatory gradient in Barrett's oesophagus: implications for disease complications.* Gut, 2002. **51**(3): p. 316-22.
6. Alexandrov, L.B., et al., *Signatures of mutational processes in human cancer.* Nature, 2013. **500**(7463): p. 415-21.
7. Le, D.T., et al., *PD-1 Blockade in Tumors with Mismatch-Repair Deficiency.* N Engl J Med, 2015. **372**(26): p. 2509-20.
8. Llosa, N.J., et al., *The vigorous immune microenvironment of microsatellite instable colon cancer is balanced by multiple counter-inhibitory checkpoints.* Cancer Discov, 2015. **5**(1): p. 43-51.
9. Muro, K., et al., *Pembrolizumab for patients with PD-L1-positive advanced gastric cancer (KEYNOTE-012): a multicentre, open-label, phase 1b trial.* Lancet Oncol, 2016. **17**(6): p. 717-26.
10. Zhang, X., et al., *Structural and functional analysis of the costimulatory receptor programmed death-1.* Immunity, 2004. **20**(3): p. 337-47.
11. Thompson, E.D., et al., *Patterns of PD-L1 expression and CD8 T cell infiltration in gastric adenocarcinomas and associated immune stroma.* Gut, 2016.
12. Wu, C., et al., *Immunohistochemical localization of programmed death-1 ligand-1 (PD-L1) in gastric carcinoma and its clinical significance.* Acta Histochem, 2006. **108**(1): p. 19-24.
13. Hou, J., et al., *Correlation between infiltration of FOXP3+ regulatory T cells and expression of B7-H1 in the tumor tissues of gastric cancer.* Exp Mol Pathol, 2014. **96**(3): p. 284-91.
14. Geng, Y., et al., *Expression of costimulatory molecules B7-H1, B7-H4 and Foxp3+ Tregs in gastric cancer and its clinical significance.* Int J Clin Oncol, 2015. **20**(2): p. 273-81.
15. Pardoll, D.M., *The blockade of immune checkpoints in cancer immunotherapy.* Nat Rev Cancer, 2012. **12**(4): p. 252-64.
16. Taube, J.M., et al., *Colocalization of inflammatory response with B7-h1 expression in human melanocytic lesions supports an adaptive resistance mechanism of immune escape.* Sci Transl Med, 2012. **4**(127): p. 127ra37.
17. Janjigian, Y.Y.B., J.C., ; Calvo, E.; Kim, J.W.; Ascierto, P.A.; Sharma, P.; Ott, P.A.; Bono, P.; Jaeger, D.; Evans, T.R.J.; De Braud, F.G.; Chau, I.; Tschaika, M.;Harbison, C.T.; Lin, C.S.; Le, D.T. , *CheckMate-032: Phase I/II, open-label study of safety and activity of nivolumab (nivo) alone or with ipilimumab (ipi) in advanced and metastatic (A/M) gastric cancer (GC).* 2016. **34**((suppl; abstr 4010)).
18. Demaria, S., E.B. Golden, and S.C. Formenti, *Role of Local Radiation Therapy in Cancer Immunotherapy.* JAMA Oncol, 2015. **1**(9): p. 1325-32.
19. Golden, E.B., et al., *Local radiotherapy and granulocyte-macrophage colony-stimulating factor to generate abscopal responses in patients with metastatic solid tumours: a proof-of-principle trial.* Lancet Oncol, 2015. **16**(7): p. 795-803.

20. Twyman-Saint Victor, C., et al., *Radiation and dual checkpoint blockade activate non-redundant immune mechanisms in cancer.* Nature, 2015. **520**(7547): p. 373-7.

21. Rutkowski, M. R. et al. *Microbially driven TLR5-dependent signaling governs distal malignant progression through tumor-promoting inflammation*. Cancer Cell, 2015. **27**: p. 27–40.

22. Goodman, A. L. et al. *Extensive personal human gut microbiota culture collections characterized and manipulated in gnotobiotic mice*. Proc. Natl. Acad. Sci. U.S.A., 2011. **108**: p. 6252–6257.
