# Supplementary material for: Neoadjuvant nivolumab or nivolumab plus LAG-3 inhibitor relatlimab in resectable esophageal/gastroesophageal junction cancer: a phase Ib trial and ctDNA analyses
Source: Nat Med. 2024 Mar 19;30(4):1023–34. doi: 10.1038/s41591-024-02877-z (PMC11031406; doi:10.1038/s41591-024-02877-z)
Supplement: Supplementary file 2 — Reporting Summary [file 41591_2024_2877_MOESM2_ESM.pdf]

# nature portfolio

Corresponding author(s): Valsamo Anagnostou

Last updated by author(s): Jan 24, 2024

# Reporting Summary

Nature Portfolio wishes to improve the reproducibility of the work that we publish. This form provides structure for consistency and transparency in reporting. For further information on Nature Portfolio policies, see our Editorial Policies and the Editorial Policy Checklist.

## Statistics

For all statistical analyses, confirm that the following items are present in the figure legend, table legend, main text, or Methods section.

| n/a | Confirmed | |
|---|---|---|
| ☐ | ☒ | The exact sample size (*n*) for each experimental group/condition, given as a discrete number and unit of measurement |
| ☐ | ☒ | A statement on whether measurements were taken from distinct samples or whether the same sample was measured repeatedly |
| ☐ | ☒ | The statistical test(s) used AND whether they are one- or two-sided<br>*Only common tests should be described solely by name; describe more complex techniques in the Methods section.* |
| ☐ | ☒ | A description of all covariates tested |
| ☐ | ☒ | A description of any assumptions or corrections, such as tests of normality and adjustment for multiple comparisons |
| ☐ | ☒ | A full description of the statistical parameters including central tendency (e.g. means) or other basic estimates (e.g. regression coefficient) AND variation (e.g. standard deviation) or associated estimates of uncertainty (e.g. confidence intervals) |
| ☐ | ☒ | For null hypothesis testing, the test statistic (e.g. *F*, *t*, *r*) with confidence intervals, effect sizes, degrees of freedom and *P* value noted<br>*Give P values as exact values whenever suitable.* |
| ☒ | ☐ | For Bayesian analysis, information on the choice of priors and Markov chain Monte Carlo settings |
| ☒ | ☐ | For hierarchical and complex designs, identification of the appropriate level for tests and full reporting of outcomes |
| ☒ | ☐ | Estimates of effect sizes (e.g. Cohen's *d*, Pearson's *r*), indicating how they were calculated |

*Our web collection on statistics for biologists contains articles on many of the points above.*

## Software and code

Policy information about availability of computer code

| | |
|---|---|
| Data collection | Data were collected, entered and managed by the clinical trial team at Johns Hopkins Sidney Kimmel Comprehensive Cancer Center in Baltimore, MD, Allegheny Health Network in Pittsburgh, PA and Baylor University Medical Center in Dallas, TX according to standard data management procedures. No software was used for data collection. |
| Data analysis | R versions 3.6.1 and 4.2.2 we used for statistical analyses. Strelka version 2.9 was used for somatic mutation calling from whole exome sequencing data. MANTIS version 1.0.4 was used for microsatellite status assessment from whole exome sequence data. Gene expression analyses were performed using the NanoString nSolver software version 4.0. Somatic variants in ctDNA were identified using VariantDx version 1.0 and annotated with OpenCRAVAT version 1.0. TCR sequence data files were analyzed in the publicly available MANAFEST analysis web application (http://www.stat-apps.onc.jhmi.edu/FEST) |

For manuscripts utilizing custom algorithms or software that are central to the research but not yet described in published literature, software must be made available to editors and reviewers. We strongly encourage code deposition in a community repository (e.g. GitHub). See the Nature Portfolio guidelines for submitting code & software for further information.

## Data

Policy information about availability of data

All manuscripts must include a data availability statement. This statement should provide the following information, where applicable:

- Accession codes, unique identifiers, or web links for publicly available datasets
- A description of any restrictions on data availability
- For clinical datasets or third party data, please ensure that the statement adheres to our policy

Next generation sequencing data from plasma and matched WBC DNA are deposited and can be retrieved from the European Genome-Phenome Archive (EGA accession number EGAS00001007299).

## Research involving human participants, their data, or biological material

Policy information about studies with human participants or human data. See also policy information about sex, gender (identity/presentation), and sexual orientation and race, ethnicity and racism.

| | |
|---|---|
| Reporting on sex and gender | Findings apply to both sexes. Sex was determined by self-reporting, both females and males were enrolled in the study and sex was not a stratification criterion for this clinical trial. |
| Reporting on race, ethnicity, or other socially relevant groupings | Findings apply to all race and ethnicity groups, as race, ethnicity or other socially relevant grouping was not an inclusion or exclusion criterion in this study. |
| Population characteristics | Patients predominantly had adenocarcinoma (87.5%), primary tumor located in the esophagus (81.3%), and nodal involvement (75.0%). Sixteen patients received nivolumab as induction for 2 cycles then in combination with chemoradiation for a total of 5 doses (Arm A). In Arm B, the first 9 of 16 patients received nivolumab and relatlimab following the same schedule as in Arm A, while the remaining 7 patients only received nivolumab and relatlimab as induction due to a protocol amendment for toxicity. The mean age of the whole cohort was 63 years old. 81.25% of patients were male and 18.75% were female. |
| Recruitment | Patients aged 18 years and older and with clinical stage II-III distal esophageal/GEJ adenocarcinoma or squamous cell carcinoma (American Joint Commission on Cancer (AJCC) 7th edition staging system) were eligible to enroll in the study. All patients had to have surgically resectable disease and be a candidate for standard of care chemoradiotherapy followed by surgery. Patients were recruited through the thoracic oncology clinics at Sidney Kimmel Comprehensive Cancer Center at Johns Hopkins (SKCCC), Charles A Sammons Cancer Center (Baylor), and at the Allegheny Health Network (AHN). There were no appreciable self-selection bias or other biases that affected recruitment. |
| Ethics oversight | The study protocol and all amendments were approved by the Institutional Review Board of Johns Hopkins University (Johns Hopkins Medicine Institutional Review Board #6) and local institutions (Allegheny Singer Research Institute and Baylor Scott & White Research Institute). Written informed consent was provided by all study participants; participants were not compensated. |

Note that full information on the approval of the study protocol must also be provided in the manuscript.

# Field-specific reporting

Please select the one below that is the best fit for your research. If you are not sure, read the appropriate sections before making your selection.

☒ Life sciences ☐ Behavioural & social sciences ☐ Ecological, evolutionary & environmental sciences

For a reference copy of the document with all sections, see nature.com/documents/nr-reporting-summary-flat.pdf

# Life sciences study design

All studies must disclose on these points even when the disclosure is negative.

| | |
|---|---|
| Sample size | Sample size was chosen such that the estimation precisions of the primary and secondary endpoints, such as safety and feasibility were consistent with the exploratory nature of the trial. |
| Data exclusions | 28 patients completed the full course of neoadjuvant therapy; four patients (one patient in Arm A and three patients in Arm B) required ICI discontinuation due to immunotherapy-related adverse events. |
| Replication | The analytical performance of the liquid biopsy next-generation sequencing assay used in this clinical trial has been previously validated with characteristics as follows: analytical sensitivity: 0.1-0.5% MAF, analytical specificity 99.998%. No template controls, that are specimens that do not contain DNA (molecular grade water only) were used during library preparation and capture steps. In addition, external controls that contained validated sequence mutations were used to ensure reproducibility across sequencing runs. |
| Randomization | This is not relevant based on the clinical trial design. |

Blinding | Laboratory investigators were blinded to the subject identifiers and clinical data while generating the research data.

# Reporting for specific materials, systems and methods

We require information from authors about some types of materials, experimental systems and methods used in many studies. Here, indicate whether each material, system or method listed is relevant to your study. If you are not sure if a list item applies to your research, read the appropriate section before selecting a response.

## Materials & experimental systems

| n/a | Involved in the study |
|---|---|
| ☐ | ☒ Antibodies |
| ☒ | ☐ Eukaryotic cell lines |
| ☒ | ☐ Palaeontology and archaeology |
| ☒ | ☐ Animals and other organisms |
| ☐ | ☒ Clinical data |
| ☒ | ☐ Dual use research of concern |
| ☒ | ☐ Plants |

## Methods

| n/a | Involved in the study |
|---|---|
| ☒ | ☐ ChIP-seq |
| ☒ | ☐ Flow cytometry |
| ☒ | ☐ MRI-based neuroimaging |

## Antibodies

Antibodies used | PD-L1 staining was performed using clone 22C3 (Agilent) and run on Roche/Ventana Benchmark Ultra with the Optiview detection kit. HER2 staining was performed using clone 4B5 (Roche) and run on Roche/Ventana Benchmark Ultra with the Ultraview detection kit. MLH1 (M1, Roche), MSH2 (G219-1129, Roche), and MSH6 (SP93, Roche) staining were performed on Roche/Ventana Benchmark Ultra with the Ultraview detection kit. PMS2 staining (A16-4 clone, Roche) was run on Roche/Ventana Benchmark Ultra with the Ultraview detection kit and Optiv amplification kit.

Validation | As per the manufacturers's websited, the antibodies used above are extensively validated.

## Clinical data

Policy information about clinical studies

All manuscripts should comply with the ICMJE guidelines for publication of clinical research and a completed CONSORT checklist must be included with all submissions.

Clinical trial registration | ClinicalTrials.gov identifier NCT03044613.

Study protocol | The full clinical trial protocol is provided as a separate attachment.

Data collection | From August 2017 to July 2021, 42 patients were screened and 32 patients were enrolled. The first and last patient were enrolled on the study on August 23, 2017 and July 1, 2021, respectively; data lock date was January 25, 2022. Patients enrolled at Johns Hopkins Sidney Kimmel Comprehensive Cancer Center in Baltimore, MD, Allegheny Health Network in Pittsburgh, PA and Baylor University Medical Center in Dallas, TX.

Outcomes | The primary endpoint of the study was safety and secondary endpoint was feasibility. Safety was measured through the proportion of evaluable patients whose worst adverse events of interest occurred within 100 days after the last dose of Nivolumab (or Nivolumab+relatlimab) or within 30 days after surgery, whichever is longer. Feasibility was assessed through the proportion of eligible patients who proceeded to surgery without substantial treatment-related delay; the latter defined as more than 11 weeks from completion of chemoradiation. RFS and OS were measured every 3 months prior to and after surgical resection. Exploratory endpoints included overall survival , recurrence-free survival , major pathological response (MPR) and pathological complete response (pCR) rates. Recurrence-free survival was defined as the time from treatment initiation to disease recurrence or death due to any cause, whichever occurred first. Overall survival was defined as the time from treatment initiation to death due to any cause. Patients were censored if no RFS or OS event occurred by the last follow-up. Pathological response was assessed semi-quantitatively using a modified Ryan scheme, as recommended by the College of American Pathologists; pCR signifies 0% residual tumor at the time of resection, while MPR signifies <10% residual tumor at the time of resection.

# Plants

| | |
|---|---|
| Seed stocks | Not applicable. |
| Novel plant genotypes | Not applicable. |
| Authentication | Not applicable. |
